# CD4^+^ T cells produce IFN-I to license cDC1s for induction of cytotoxic T-cell activity in human tumors

**DOI:** 10.1038/s41423-024-01133-1

**Published:** 2024-02-21

**Authors:** Xin Lei, Daniël C. de Groot, Marij J. P. Welters, Tom de Wit, Ellen Schrama, Hans van Eenennaam, Saskia J. Santegoets, Timo Oosenbrug, Annemarthe van der Veen, Joris L. Vos, Charlotte L. Zuur, Noel F. C. C. de Miranda, Heinz Jacobs, Sjoerd H. van der Burg, Jannie Borst, Yanling Xiao

**Affiliations:** 1 Department of Immunology, Leiden University Medical Center, Leiden, The Netherlands; 2 Oncode Institute, Leiden University Medical Center, Leiden, The Netherlands; 3 Department of Tumor Biology and Immunology, The Netherlands Cancer Institute, Amsterdam, The Netherlands; 4 Department of Medical Oncology, Oncode Institute, Leiden University Medical Center, Leiden, The Netherlands; 5 IMMIOS B.V., Oss, The Netherlands; 6 Division of Medical Oncology, The Netherlands Cancer Institute, Amsterdam, The Netherlands; 7 Department of Otorhinolaryngology Leiden University Medical Center, Leiden, The Netherlands; 8 Department of Pathology, Leiden University Medical Center, Leiden, The Netherlands

**Keywords:** CD4+ T-cell help, cDC1 licensing, IFN-I signaling, CD40 costimulation, (cross-)presentation, CTL priming, Tumor control, Tumour immunology, Dendritic cells, Cellular immunity

## Abstract

CD4^+^ T cells can "help” or "license” conventional type 1 dendritic cells (cDC1s) to induce CD8^+^ cytotoxic T lymphocyte (CTL) anticancer responses, as proven in mouse models. We recently identified cDC1s with a transcriptomic imprint of CD4^+^ T-cell help, specifically in T-cell-infiltrated human cancers, and these cells were associated with a good prognosis and response to PD-1-targeting immunotherapy. Here, we delineate the mechanism of cDC1 licensing by CD4^+^ T cells in humans. Activated CD4^+^ T cells produce IFNβ via the STING pathway, which promotes MHC-I antigen (cross-)presentation by cDC1s and thereby improves their ability to induce CTL anticancer responses. In cooperation with CD40 ligand (L), IFNβ also optimizes the costimulatory and other functions of cDC1s required for CTL response induction. IFN-I-producing CD4^+^ T cells are present in diverse T-cell-infiltrated cancers and likely deliver “help” signals to CTLs locally, according to their transcriptomic profile and colocalization with “helped/licensed” cDCs and tumor-reactive CD8^+^ T cells. In agreement with this scenario, the presence of IFN-I-producing CD4^+^ T cells in the TME is associated with overall survival and the response to PD-1 checkpoint blockade in cancer patients.

## Introduction

Cancer immunotherapies primarily aim to boost CD8^+^ T-cell responses because CTLs can directly kill tumor cells. However, CD4^+^ T cells are also essential for antitumor immunity because they provide “help” to innate immune cells, B cells and CD8^+^ T cells. CD4^+^ T cells help promote the clonal expansion and effector and effector/memory differentiation of CD8^+^ T cells [1–3]. As a result of CD4^+^ T-cell help, the migratory, invasive and cytotoxic capacities of effector CTLs that enable them to eliminate tumors are optimized [4]. CD4^+^ T-cell help is known to be relayed to CD8^+^ T cells via DCs via a process called “DC licensing” [1, 2], but only recently has the cDC1 subset been identified as the requisite intermediate in both mice [5] and humans [6]. In mouse studies, CD4^+^ T cells were found to license DCs via the CD40 ligand (L)-CD40 signaling pathway [5, 7, 8], which upregulates the expression of the costimulatory ligands CD80/86 and CD70 and increases DC survival [9–11]. The improved costimulatory status of cDC1s contributes to optimal CTL effector and memory differentiation of CD8^+^ T cells via CD28- and CD27 costimulation [4, 5]. Mouse studies have also shown that IL-12 production is an important feature of licensed DCs [2].

Our transcriptome data from human cDC1s revealed that antigen (cross-)presentation pathways are upregulated by CD4^+^ T-cell help, and functional studies confirmed that cDC1s but not other human DC subsets can optimally cross-prime CTL responses to cell-associated antigens in response to CD4^+^ T-cell help [6]. This finding aligns with the idea gleaned from mouse studies that cDC1s have a unique ability to cross-present cell-associated antigens [12, 13], which is key for CTL-based antitumor immunity. Among the biological processes most strongly upregulated in “helped/licensed” human cDC1s were cytokine-related pathways. CD4^+^ T cells amplify IL-15R/IL-15 expression in human cDC1s [6, 14], which promotes the expansion and survival of memory CD8^+^ T-cell pools [15–17]. In addition, CD4^+^ T-cell help results in upregulation of CXCL9/10/11 in “helped/licensed” cDC1s. These chemokines play essential roles in the spatial distribution and function of CXCR3^+^ effector (memory) T cells [18].

Importantly, we identified the transcriptomic signature of “helped/licensed” cDC1s [6] in the tumor microenvironment (TME) of many cancer types that presented a T-cell infiltrated phenotype according to a pancancer analysis by Luca et al. [19]. In that study, Luca et al. identified carcinoma ecotypes (CEs) by transcriptomic analysis and revealed that CE9 is correlated with good prognosis in a wide variety of cancers. According to this analysis, CE9 uniquely contained CD4^+^ T cells, CD8^+^ T cells and DCs, which we found to include “helped/licensed” cDC1s. Accordingly, our “helped” cDC1 signature [6] correlated with a good prognosis and response to PD-1-targeting checkpoint immunotherapy. On the basis of this work, we propose that CD4^+^ T-cell help to support the CTL response can occur not only during T-cell priming in lymph nodes but also in the TME.

Here, we report that in the human system, CD4^+^ T cells use not only CD40L but also IFNβ to license cDC1s for the induction of antitumor immunity. In general, myeloid cells, rather than lymphocytes, are thought to be the main producers of type I interferon (IFN-I) upon stimulation by pathogen/danger-associated molecular patterns (P/DAMPs) [20–22]. However, we found evidence for cDC1 licensing by IFN-I-producing CD4^+^ T cells in the TME of multiple cancer types with T-cell infiltration, which correlated with the response to PD-1 blockade and overall patient survival. Our data reveal how CD4^+^ T cells can optimize antigen cross-presentation by cDC1s within immune cell hubs/niches in the TME where P/DAMPs may be limited. In cooperation with CD4^+^ T-cell-derived CD40L signals, IFN-I signals thereby enable the induction of CTL-based antitumor immunity.

## Results

### CD3/CD28-stimulated CD4^+^ T cells produce IFNβ via STING pathway activation

**Fig. 1:**
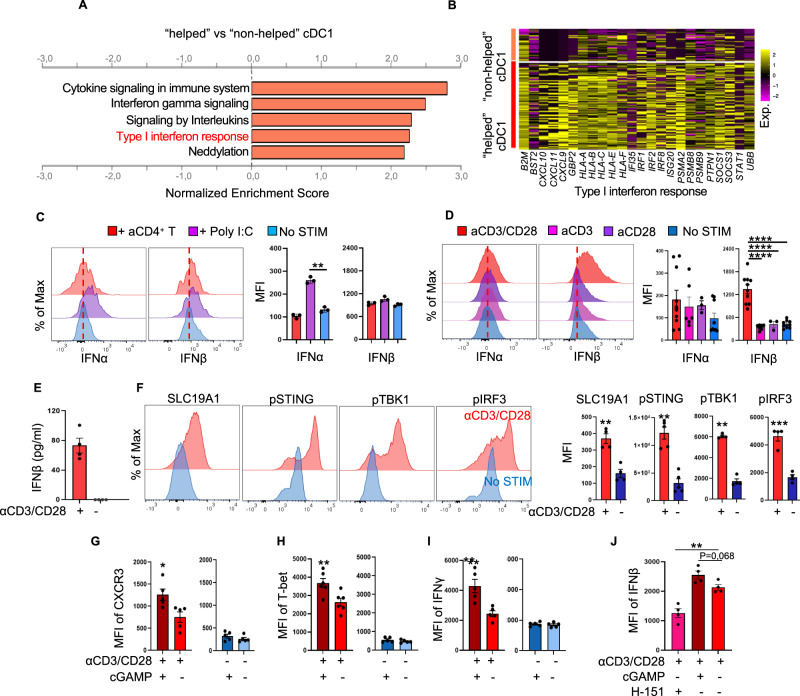
CD3/CD28-activated CD4^+^ T cells produce IFNβ via STING pathway activation, which induces an IFN-I response in “helped” cDC1s. **A**, **B** Purified cDC1s were cocultured with activated (“helped”) or naive (“non-helped”) CD4^+^ T cells and then subjected to 10X Genomics hashtag single-cell (sc) mRNA-seq analysis [6]. **A** Gene set enrichment analysis (GSEA) of the 577 DEGs in the cDC1 “help” signature denoting enrichment of the indicated pathways (FDR < 0.05) using the Reactome database. **B** Heatmap depicting scaled expression values of the genes involved in the “helped” cDC1-enriched IFN-I response pathway. **C**–**J** Purified cDC1s were stimulated with anti-CD3/CD28-activated CD4^+^ T cells or poly (I:C). Naive CD4^+^ T cells were stimulated with anti-CD3/CD28 antibodies alone or in combination as indicated for 48**–**72 h. A STING inhibitor (H-151) or STING agonist (2′3′-cGAMP) was added for the last 8 h of culture where indicated. The cells were analyzed by flow cytometry. Histograms and median fluorescence index (MFI) values of intracellular IFNα and IFNβ expression in cDC1s (**C**) and CD4^+^ T cells (**D**) under the indicated conditions. **E** The supernatant concentration of IFNβ protein produced by CD4^+^ T cells under the indicated conditions was measured via ELISA. **F** Histograms and MFI values of intracellular SLC19A1, phospho-STING, phospho-TBK1 and phospho-IRF3 protein levels in CD4^+^ T cells under the indicated conditions. MFI values of CXCR3 (**G**), T-bet (**H**), IFNγ (**I**) and IFNβ (**J**) in CD4^+^ T cells under the indicated conditions. The data were pooled from multiple independent experiments per donor (*n* = 3 in **C**; *n* = 4**–**7 in **D**; *n* = 4 in **E**, **F** and **G**; and *n* = 5 in **H–J**). *P* < 0.05*, *P* < 0.01**, *P* < 0.001***, *P* < 0.0001**** (one-way ANOVA for **C**, **D** and **G**; two-tailed Mann‒Whitney *U* test for **E**, **H–J**). The data are shown as the means ± standard errors of the mean (SEMs)

We identified the transcriptomic “helped/licensed” cDC1 signature by coculturing human cDC1s purified from peripheral blood with either naive ("non-helped”) or CD3/CD28-activated CD4^+^ T cells (“helped”). To determine the molecular mechanism of cDC1-licensing, we performed gene set enrichment analysis (GSEA) on the 577 differentially expressed genes (DEGs) (Supplementary Fig. 1A) identified in “helped” cDC1s versus “non-helped” cDC1s [6]. In “helped” cDC1s, in addition to cytokine/interleukin signaling, “IFN-I response” and “Interferon gamma (IFNγ) signaling”, which contained many overlapping genes, were upregulated (Fig. 1A; Supplementary Fig. 1B). The IFN-I response gene set included chemokines (*CXCL9/10/11*), MHC class I (MHC-I) molecules (*HLA-A/B/C/E/F* and *β2m*), proteasome subunits (*PSMA2* and *PSMB9*), IFN regulatory factors (*IRF1/2/8*), and IFN-induced molecules (*ISG20, IFI35*, and *STAT1*) (Fig. 1B; Supplementary Table 1). Because CD4^+^ T cells and cDC1s were the only two cell types in the cocultures used for scRNAseq, we hypothesized that CD4^+^ T cells were the source of the IFN-I, although in vivo, DCs [23] rather than CD4^+^ T cells are reported to produce IFN-I. To test this hypothesis, we purified cDC1s **(**Supplementary Fig. 1C) and naive CD4^+^ T cells **(**Supplementary Fig. 1D) from human blood and did or did not (No STIM) activate the T cells with agonistic antibodies against CD3 and CD28 to mimic T-cell receptor (TCR)/CD3- and CD28 signaling. CD4^+^ T cells were cultured in the presence of IL-2, IL-7 and IL-15, which maintained their survival (Supplementary Fig. 1E–H). cDC1s were stimulated with either activated CD4^+^ T cells or Poly (I:C), and the intracellular protein expression of IFNα and IFNβ was analyzed by flow cytometry (Supplementary Fig. 2A). No upregulation of IFNα or IFNβ was detected in cDC1s cocultured with activated CD4^+^ T cells, whereas significant upregulation of IFNα but not IFNβ was detected in cDC1s upon TLR3 stimulation by Poly (I:C) (Fig. 1C; Supplementary Fig. 2A). In contrast, IFNβ but not IFNα was significantly upregulated in CD4^+^ T cells stimulated with CD3/CD28 but not in those stimulated with CD3 or CD28 alone (Fig. 1D; Supplementary Fig. 2B). IFN-I production by activated CD4^+^ T cells was confirmed by ELISA measuring the IFNβ concentration in the culture medium (Fig. 1E). These findings are consistent with our hypothesis that activated CD4^+^ T cells are the source of the IFN-I that may have induced the response signature in cDC1s.

In myeloid cells, the STING pathway is an important inducer of IFN-I production [24], and one study in mice reported that T cells produce IFN-I [25]. Recently, cGAMP was identified as a soluble, extracellular immune transmitter derived from tumor cells [26] and antigen-presenting cells [27], and SLC19A1 [28] was identified as an importer of cGAMP for activation of STING in target cells. We therefore tested whether IFN-I production by activated CD4^+^ T cells is also mediated via the STING pathway. Increased SLC19A1, phospho-STING, phospho-TBK1, and phospho-IRF3 protein levels were detected in CD4^+^ T cells stimulated with CD3/CD28 (Supplementary Fig. 2C; Fig. 1F). We further investigated STING pathway activation and IFN-I production in CD3/CD28-stimulated CD4^+^ T cells over a time course of 72 h (Supplementary Fig. 3A, B). Increased levels of phospho-STING, phospho-TBK1 and phospho-IRF3 were detected beginning at 24 h (Supplementary Fig. 3C–E), and upregulation of IFNβ was detected beginning at 48 h (Supplementary Fig. 3F), a time point that coincided with T-cell blast formation, as indicated by the increased forward scatter and side scatter (Supplementary Fig. 3B). To further analyze the involvement of the STING pathway in IFN-I production by activated CD4^+^ T cells, a STING agonist (2′3′-cGAMP) and a STING inhibitor (H-151) were added under different stimulation conditions. The control samples were CD3/CD28-activated CD4^+^ T cells without STING agonist/inhibitor treatment (Supplementary Fig. 4A). The protein levels of IFNβ, phospho-STING and phospho-TBK1 were increased in CD3/CD28-activated CD4^+^ T cells upon STING stimulation by cGAMP (Fig. 1G; Supplementary Fig. 4B, C). Consistently, immunoblotting confirmed that signaling intermediates in the STING pathway (i.e., phospho-STING and phospho-TBK1) and IFN-I-stimulated gene products (i.e., IRF7 and ISG60) were induced in CD3/CD28-stimulated CD4^+^ T cells (Supplementary Fig. 4D). Taken together, these results indicate that human CD4^+^ T cells produce IFN-I via the STING pathway after CD3/CD28-mediated activation. cGAMP also induced the expression of the markers CXCR3, T-bet and IFNγ, which indicate T helper 1 (Th1) differentiation in CD3/CD28-activated CD4^+^ T cells (Fig. 1G–I; Supplementary Fig. 4E). Upon STING inhibition with H-151, the expression level of IFNβ was significantly decreased compared to that in the control cells (Fig. 1J). We conclude from these collective data that in CD3/CD28-stimulated CD4^+^ T cells, the STING pathway is activated beginning at 24 h, and its activation leads to IFNβ production.

### IFN-I receptor signaling in cDC1s confers key aspects of CD4^+^ T-cell help at the transcriptomic and protein levels

**Fig. 2:**
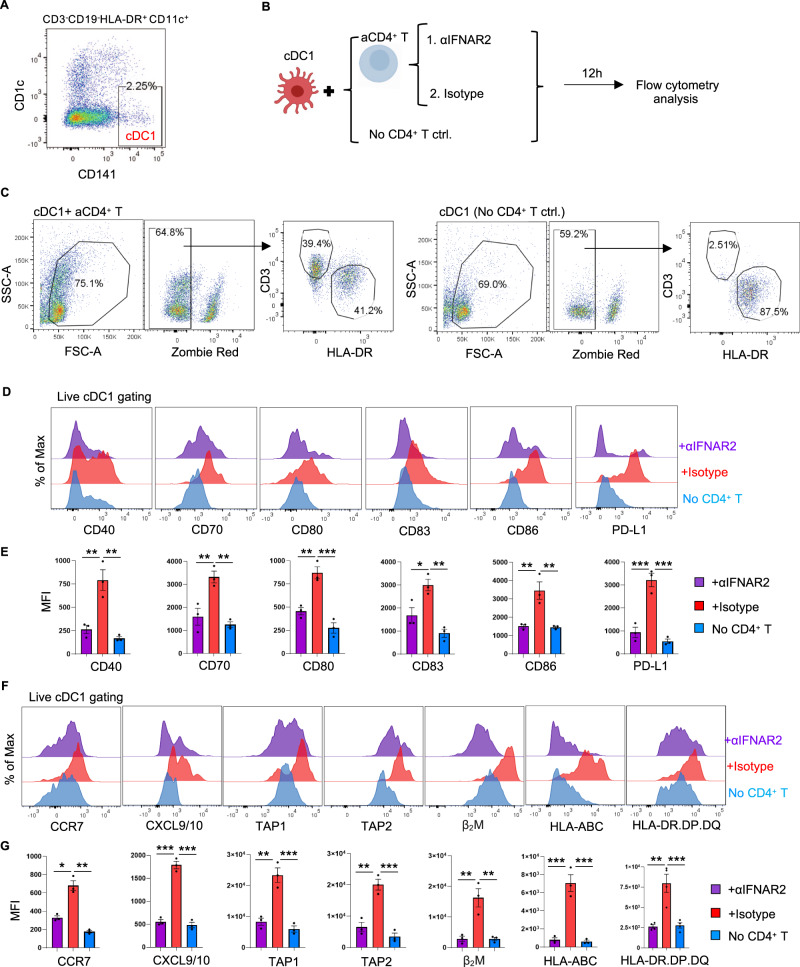
The response of cDC1s to CD4^+^ T-cell help is significantly compromised upon IFNAR2 blockade. Purified cDC1s were stimulated with or without CD3/CD28-activated CD4^+^ T cells. An anti-IFNAR2 blocking antibody (5 μg/ml) or IgG_2A_ isotype control was added as indicated. The expression of key molecules in the cDC1 “help” signature [6] was analyzed via flow cytometry. **A** Gating strategy for cDC1 sorting. **B** Schematic illustration of the cDC1-CD4^+^ T-cell coculture system. **C** Gating strategies for flow cytometric analysis of cDC1s after coculture with CD4^+^ T cells. **D**, **F** Histograms depicting the expression of the indicated cDC1 “help” signature markers under the indicated conditions. **E**, **G** MFI values for the expression of the indicated cDC1 “help” signature markers under the indicated conditions. The data were pooled from three (*n* = 3) independent experiments in **E**, **G**. *P* < 0.05*, *P* < 0.01**, *P* < 0.001*** (one-way ANOVA). The data are shown as the means ± SEMs

**Fig. 3:**
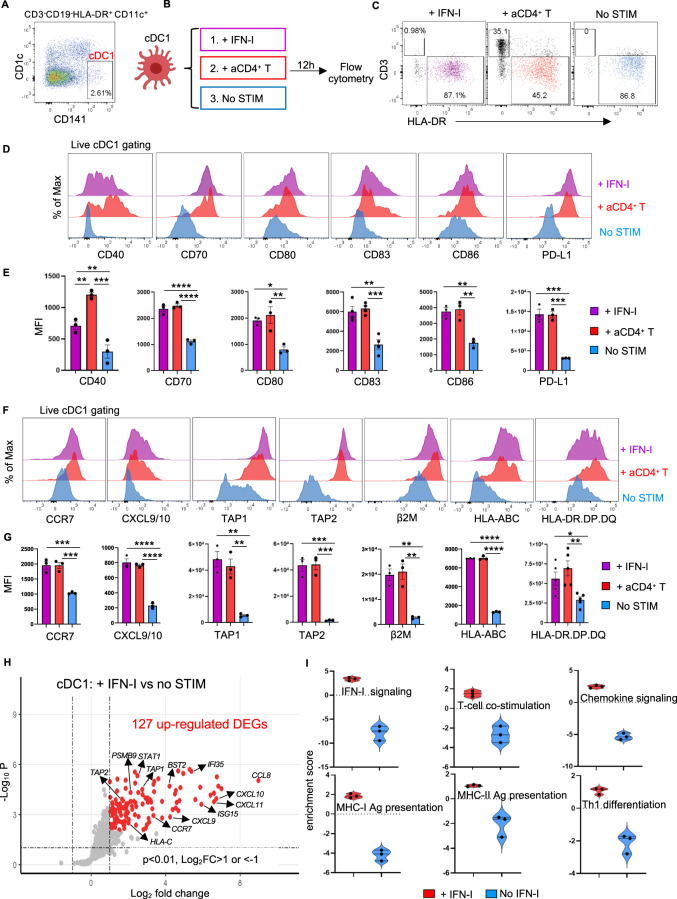
IFN-I-stimulated cDC1s share many features with “helped” cDC1s. **A–G** Purified cDC1s were stimulated with IFN-I (100 U/ml IFNα + 150 pg/ml IFNβ) or with CD3/CD28-activated CD4^+^ T cells. The expression of key molecules in the cDC1 “help” signature [6] was analyzed by flow cytometry. **A** Gating strategy for cDC1 sorting. **B** Schematic illustration of the experimental procedure. **C** Gating strategies for flow cytometric analysis of cDC1s after stimulation. **D**, **F** Histograms depicting the expression of the indicated cDC1 “help” signature markers under the indicated conditions. **E**, **G** MFI values for the expression of the indicated cDC1 “help” signature markers under the indicated conditions. **H**, **I** Purified cDC1s cultured with or without IFN-I were subjected to NanoString nCounter analysis. **H** Volcano plot depicting the DEGs between cDC1s with and without IFN-I stimulation. The genes indicated in red are significantly differentially expressed, with a *p* value < 0.01 and a log_2_-fold change (FC)>1. **I** Violin plots depicting pathway annotations of adaptive immune response genes enriched in IFN-I-stimulated cDC1s. The data were pooled from three (*n* = 3) independent experiments in (**E**, **G**). The data were obtained from three (*n* = 3) independent biological samples in (**H**, **I**). *P* < 0.05*, *P* < 0.01**, *P* < 0.001***, *P* < 0.0001**** (one-way ANOVA). The data are shown as the means ± SEMs

We next tested whether cDC1s indeed respond to IFN-I produced by CD4^+^ T cells at the protein level. For this purpose, purified cDC1s were cocultured with activated CD4^+^ T cells (Fig. 2A) in the presence or absence of an anti-IFNα/β receptor 2 (IFNAR2) blocking antibody, and the cDC1 phenotype was subsequently analyzed by flow cytometry (Fig. 2B, C). Coculture with activated CD4^+^ T cells increased the expression of the following proteins in cDC1s: the costimulatory/coinhibitory molecules CD40, CD70, CD80, CD83, CD86 and PD-L1 (Fig. 2D, E); the chemokine receptor CCR7; the chemokines CXCL9/10; and antigen presentation pathway components, including transporter associated with antigen presentation 1 and 2 (TAP1/2), β2-microglobulin (M), HLA-A/B/C and the MHC class II molecule HLA-DR.DP.DQ (Fig. 2F, G). Importantly, IFNAR2 blockade prevented these changes (Fig. 2F, G). In addition, the direct impact of IFN-I on cDC1s was investigated. Purified cDC1s were stimulated with or without IFN-I, and cDC1s cultured alone or cultured with activated CD4^+^ T cells were used as controls (Fig. 3A–C). The phenotype of cDC1s responding to IFN-I was similar to the phenotype of cDC1s responding to activated CD4^+^ T cells (Fig. 3D–G). Many of the genes identified by scRNA-seq in “helped” cDC1s were assigned to both the IFN-I and IFNγ signaling pathways (Fig. 1A, Supplementary Table 1). Therefore, we assessed the impact of CD4^+^ T cells on the “helped” cDC1 phenotype in the absence or presence of blocking antibodies against the receptors of IFN-I and IFNγ, i.e., IFNAR2 and IFNGR1, respectively (Supplementary Fig. 5A). Blocking either IFNAR2 or IFNGR1 prevented the upregulation of several “help” signature molecules in cDC1s to a similar extent (Supplementary Fig. 5B). However, the expression of the MHC class I antigen presentation pathway components TAP1/2, β2-M and HLA-A/B/C was more strongly reduced by IFNAR2 blockade than by IFNGR1 blockade (Supplementary Fig. 5B).

To gain broader insight into the impact of IFN-I on cDC1s, we performed NanoString analysis using a host immune response panel (Supplementary Fig. 5C). The results of this gene expression analysis indicated that key molecules and pathways upregulated in “helped” cDC1s [6], such as chemokine signaling, MHC class I and II antigen presentation and T-cell differentiation pathways, were also upregulated in IFN-I-stimulated cDC1s (Fig. 3H, I; Supplementary Fig. 5D; Supplementary Table 2). Additionally, when directly comparing the effects of IFN-I stimulation and IFNγ stimulation on cDC1s, NanoString analysis clearly indicated that both IFN-I and IFNγ partially upregulated distinct transcripts, including those of TAP1/2 and proteasome subunits, in cDC1s (Supplementary Fig. 5D, E; Supplementary Table 2). Our findings indicate that IFN-I and IFNγ produced by CD4^+^ T cells contribute to multiple aspects of cDC1 licensing, with a greater role for IFN-I in promoting features of MHC class I antigen (cross-)presentation.

### IFNβ produced by CD4^+^ T cells improves the cDC1-mediated CTL response to cell-associated tumor antigens

**Fig. 4:**
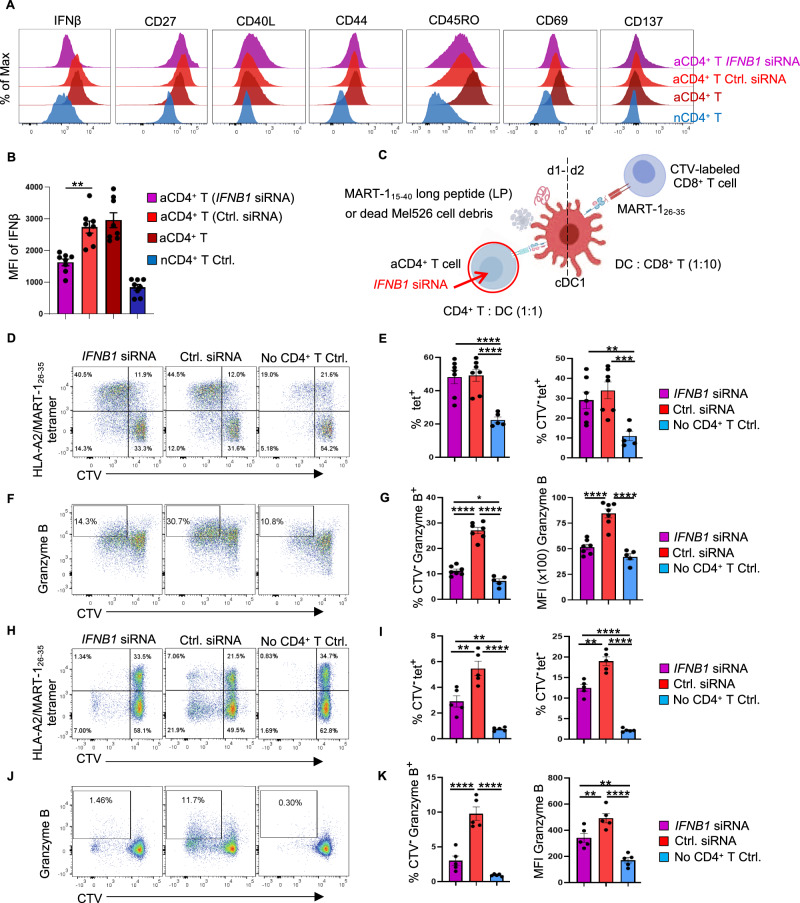
IFN-I produced by activated CD4^+^ T cells is important for the cDC1-mediated CTL response to cell-associated tumor antigens. A tumor antigen-specific CTL priming system [6] was used to investigate the impact of IFN-I produced by activated CD4^+^ T cells on the cross-presentation and cross-priming abilities of cDC1s. IFNβ expression was downregulated in CD4^+^ T cells by siRNA. **A** Flow cytometry histograms depicting intracellular IFNβ expression and the surface expression of the indicated markers identifying effector T cells under the indicated conditions. **B** MFI values for intracellular IFNβ expression under the indicated conditions. **C** Schematic illustration of the tumor antigen-specific CTL priming system. **D** CD8^+^ T-cell proliferation induced by MART-1_15**–**40_ long peptide (LP) based on CTV dilution. **E** Percentages of MART-1_26**–**35_/HLA-A2-specific (tet^postive^) cells among CD8^+^ T cells or CTV^negative^ CD8^+^ T cells in the MART-1_15**-**40_ LP setting. **F** CTL response to MART-1_15**–**40_ LP based on intracellular Granzyme B staining. **G** Percentages of Granzyme B^+^ cells among CTV^negative^ CD8^+^ T cells and MFI values of Granzyme B in the MART-1_15-40_ LP setting. **H** CD8^+^ T-cell proliferation in response to dead Mel526 cell debris based on CTV dilution. **I** Percentages of MART-1_26-35_/HLA-A2-specific (tet^positive^) cells and tet^negative^ cells among CTV^negative^ CD8^+^ T cells in the dead Mel526 cell debris setting. **J** CTL response to dead Mel526 cell debris based on intracellular Granzyme B staining. **K** Percentages of Granzyme B^+^ cells among CTV^negative^ CD8^+^ T cells and MFI values of Granzyme B in the dead Mel526 cell debris setting. The data were pooled from eight (*n* = 8 in **B**), seven (*n* = 7 in **E**, **G**) or five (n = 5 in **I**, **K**) independent experiments. *P* < 0.05*, *P* < 0.01**, *P* < 0.001***, *P* < 0.0001**** (one-way ANOVA). The data are shown as the means ± SEMs

Our finding that IFN-I signaling is involved in cDC1 licensing agrees with the findings of mouse studies [29–31]. To investigate whether IFNβ produced by CD4^+^ T cells plays a role in the cDC1-mediated CTL response in humans, we employed our in vitro tumor antigen-specific CTL priming platform [6]. Primary cDC1, CD4^+^ and CD8^+^ T cells were purified from human peripheral blood by flow cytometry. Transfection of CD4^+^ T cells with *IFNB1* siRNA (Supplementary Fig. 6A) significantly reduced IFNβ mRNA (Supplementary Fig. 6B) and IFNβ protein (Supplementary Fig. 6C; Fig. 4A, B) levels in CD3/CD28-activated CD4^+^ T cells. *IFNB1* siRNA did not alter the expression of the key activation/effector markers CD27, CD40L, CD44, CD45RO, CD69 or CD137 in CD3/CD28-activated CD4^+^ T cells (Fig. 4A; Supplementary Fig. 6D).

To perform the CTL priming assay, CD8^+^ T cells were retrovirally transduced to express a TCR specific for the MART-1_26-35_ peptide in the context of HLA-A2. According to our previous study [6], these retrovirally transduced CD8^+^ T cells exhibit a T-stem cell memory (T_SCM_) phenotype, which indicates that they have not yet undergone effector differentiation [32]. In addition, CD8^+^ T cells were labeled with the fluorescent dye CellTrace Violet (CTV) to monitor proliferation (Fig. 4C). cDC1s were loaded with MART-1_15-40_ long peptide (LP) or dead MART-1-expressing Mel526 cell debris to create antigen cross-presentation settings and were then exposed to activated CD4^+^ T cells treated with or without *IFNβ* siRNA. Upon cDC1-mediated priming with MART-1_15-40_ LP, the proliferation of MART-1_26-35_-specific (tet^+^) CD8^+^ T cells did not differ between the control siRNA- and *IFNB1* siRNA-transfected CD4^+^ T-cell settings (Fig. 4D, E; Supplementary Fig. 6E). However, Granzyme B production by proliferating CTV^-^CD8^+^ T cells was significantly reduced in the *IFNB1* siRNA-transfected CD4^+^ T-cell setting (Fig. 4F, G). Strikingly, in the tumor cell-associated antigen cross-presentation setting with Mel526 cell debris, both the proliferation and Granzyme B production of MART-1_26-35_-specific CD8^+^ T cells and non-MART-1_26-35_-specific CD8^+^ T cells were significantly compromised in the *IFNB1* siRNA-transfected CD4^+^ T-cell condition (Fig. 4H–K). In addition, the percentage of CTV^-^tet^-^ CD8^+^ T cells (likely specific for tumor antigens other than MART-1) was significantly decreased (Fig. 4H–K). These data indicate that IFNβ produced by activated CD4^+^ T cells increases the ability of cDC1s to cross-present antigens and cross-prime a CTL response, particularly in a setting where the antigen source for cDC1s is dead tumor cells.

### Human cDC1 licensing via IFNβ is CD40L independent and complementary to CD40 signaling in CTL cross-priming

**Fig. 5:**
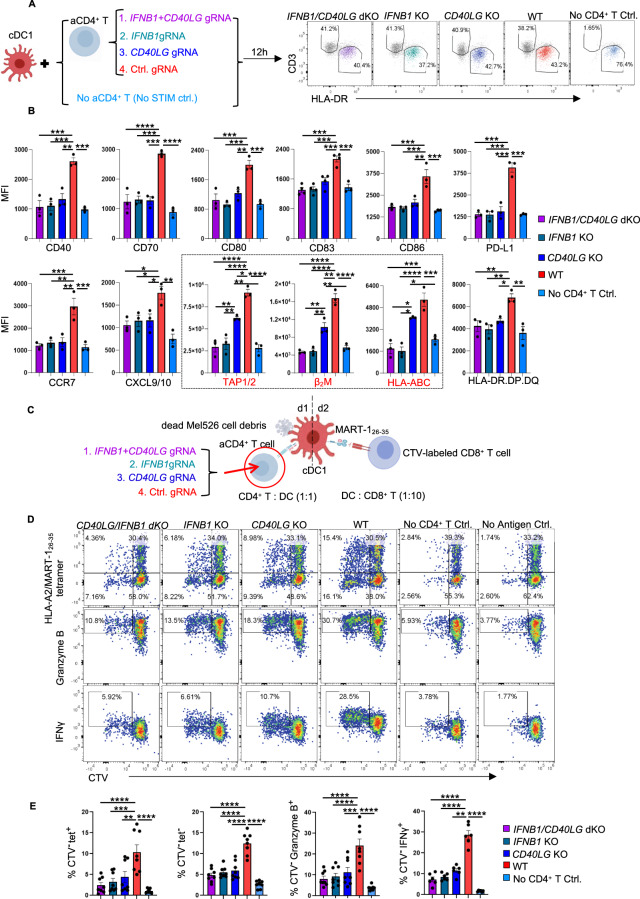
CD4^+^ T-cell help provided via IFNβ rather than via CD40 signaling promotes MHC-I antigen cross-presentation in cDC1s. Purified naive CD4^+^ T cells were transfected with Cas9/ctrl. gRNA, Cas9/*IFNB1* gRNA, Cas9/*CD40LG* gRNA or the Cas9/*IFNB1+CD40LG* gRNA ribonucleoprotein (RNP) complex were subsequently stimulated with anti-CD3/CD28 antibodies. **A** Schematic illustration of the cDC1-CD4^+^ T-cell coculture system and the gating strategies for flow cytometric analysis of the cDC1 response. **B** MFI values for the expression of the indicated cDC1 “help” signature markers under the indicated conditions. The black box highlights the molecules involved in MHC-I antigen presentation. **C**–**E** The tumor antigen-specific CTL priming system [6] was used to investigate the impact of IFN-I and CD40L produced by CD4^+^ T cells on cDC1-mediated CTL priming. Dead Mel526 cell debris was used as an antigen source. **C** Schematic illustration of the experimental procedures. (**D**) CD8^+^ T-cell proliferation based on CTV dilution and the CTL response based on intracellular Granzyme B and IFNγ staining. **E** Percentages of MART-1_26-35_/HLA-A2-specific (tet^+^) cells, tet^(-)^ cells, and Granzyme B^+^ or IFNγ^+^ cells among CTV^(-)^CD8^+^ T cells. The data were pooled from three (*n* = 3 in **B**) or 8 (*n* =  8 in **E**) independent experiments. *P* < 0.05*, *P* < 0.01**, *P* < 0.001*** (one-way ANOVA). The data are shown as the means ± SEMs

Upon activation, CD4^+^ T cells upregulate membrane-bound CD40L, which can trigger increased interactions with CD40 on cDC1s for cDC1 licensing [1, 2]. We investigated the relative contributions of IFNβ and CD40L produced by CD4^+^ T cells to the licensing of human cDC1s. CD4^+^ T cells were rendered genetically deficient in IFNβ and/or CD40L using CRISPR‒Cas9 gene editing, which did not significantly affect the expression of key activation markers other than IFNβ and/or CD40L (Supplementary Fig. 7A–C). We compared the impact of CD3/CD28-activated wild-type (WT) and IFNβ- and/or CD40L-deficient CD4^+^ T cells on cDC1 licensing by flow cytometry (Fig. 5A). Strikingly, the loss of either IFNβ or CD40L in activated CD4^+^ T cells reduced the protein levels of CD40, CD70, CD80, CD83, CD86, PD-L1, MHC class II, CCR7 and CXCL9/10 in cocultured cDC1s compared to unedited WT activated CD4^+^ T cells. The combined loss of IFNβ and CD40L did not further compromise the cDC1 response in these aspects, suggesting redundancy between IFNβ and CD40 signaling in cDC1s (Fig. 5B; Supplementary Fig. 7D). However, compared with CD40L-deficient CD4^+^ T cells, IFNβ-deficient CD4^+^ T cells had a reduced ability to upregulate key molecules involved in MHC class I antigen (cross-)presentation, such as TAP1/2, β2M and HLA-A/B/C (Fig. 5B; Supplementary Fig. 7D), in cDC1s. Consistently, blocking CD40 (Supplementary Fig. 8A) had a minimal impact on the upregulation of “help” signature proteins in IFN-I-stimulated cDC1s, as shown by flow cytometry (Supplementary Fig. 8B), and on the host response panel gene set, as determined by NanoString analysis (Supplementary Fig. 8C). These results indicate a CD40-independent role of IFN-I signaling in cDC1 licensing.

**Fig. 6:**
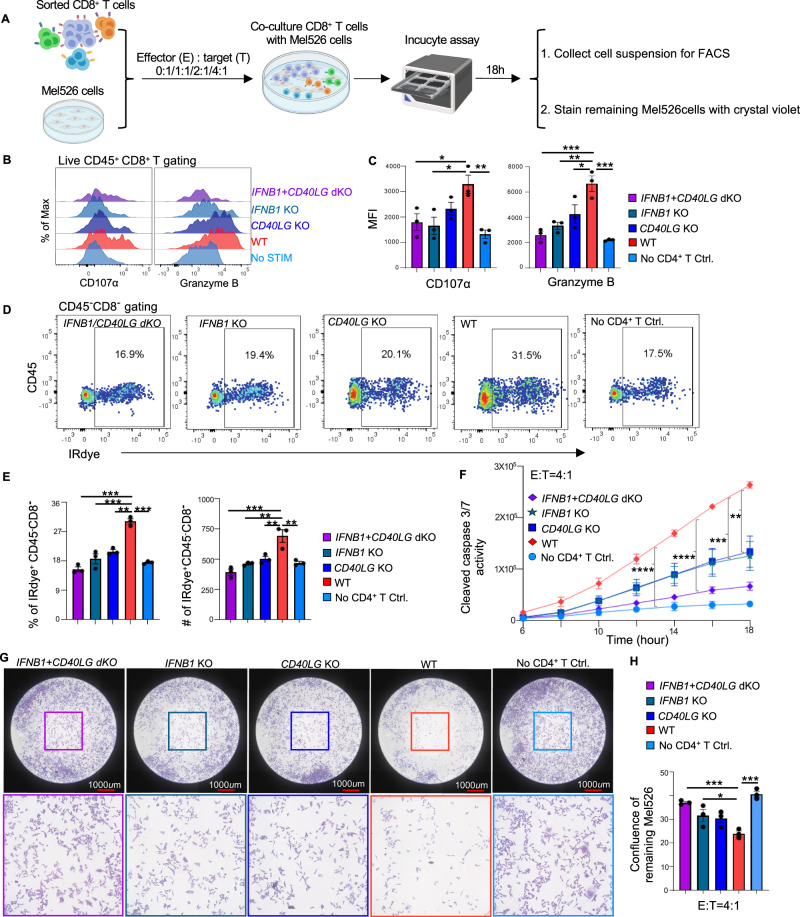
Loss of CD40L and IFNβ in CD4^+^ T cells significantly impairs the cDC1-mediated tumoricidal capacity of helped CTLs. A cytotoxicity assay was performed using live MART-1-specific CD8^+^ T cells purified from the CTL priming system and Mel526 tumor cells cultured at different effector/target (E/T) ratios for 18 h, and the data were analyzed by IncuCyte S3 software. At the end of the assay, the cell suspensions were pooled and analyzed via flow cytometry. The remaining Mel526 cells in each well were fixed and stained with crystal violet. **A** Schematic illustration of the cytotoxicity assay procedures. **B**, **C** Histograms and MFI values for the expression of the indicated markers in CD8^+^ T cells after the cytotoxicity assay. **D** Flow cytometric plots depicting total and dead Mel526 cells, as indicated by the CD45^−^CD8^−^ and IRdye^+^CD45^−^CD8^−^ populations, respectively, under the indicated conditions. **E** Percentage and number (#) of dead tumor cells in the cell suspension. **F** Caspase 3/7 activity in tumor cells during the cytotoxicity assay under the indicated conditions at an E/T ratio of 4/1. **G** Representative CCD micrographs depicting the Mel526 cells remaining in the plate 18 h post coculture under the indicated conditions at an E/T ratio of 4/1. **H** Confluence of the remaining Mel526 cells cultured under the indicated conditions at an E/T ratio of 4/1. The data were pooled from three (*n* = 3) independent experiments, each with technical duplicates. One-way ANOVA in (**C**, **E**, **H**). One-way repeated measures ANOVA in (**F**). *P* < 0.05*, *P* < 0.01**, *P* < 0.001***, *P* < 0.0001****. The data are shown as the means ± SEMs

Next, we evaluated the contributions of IFNβ and CD40L to the licensing of cDC1s for the induction of a CTL response to tumor cell-associated antigens (Fig. 5C; Supplementary Fig. 9A–C). Loss of either IFNβ or CD40L in activated CD4^+^ T cells significantly reduced the priming of antigen-specific CD8^+^ T cells in terms of both proliferation and CTL effector differentiation, as indicated by quantification of Granzyme B and IFNγ production **(**Fig. 5D, E; Supplementary Fig. 9C). We also investigated the killing capacity of CTLs primed by cDC1s under conditions of loss of IFNβ and/or CD40L (Fig. 5A, C). For this purpose, we used the IncuCyte platform, which allows real-time, automated monitoring of live/dead cells in acquired images and quantitative measurement of cell death [33] based on Caspase-3/7 activity (Fig. 6A). TCR-transduced HLA-A2/Mart-1-specific CD8^+^ T cells that had been primed with cDC1s for 6 days were isolated from the culture via FACS and added to Mel526 tumor cells at different effector (E):target (T) ratios. After 18 h of imaging, the suspended cells were analyzed via flow cytometry, and the Mel526 cells remaining in the plate were fixed and stained (Fig. 6A; Supplementary Fig. 9D–F). The IncuCyte assay revealed an E:T ratio-dependent increase in the number of apoptotic Mel526 cells over time (Supplementary Fig. 9G, H) when CD8^+^ T cells had been primed by “helped” cDC1s but not when they had been primed by “non-helped” cDC1s or had not encountered antigen (Supplementary Fig. 9I). We then evaluated the killing capacity of CD8^+^ T cells primed with “helped” cDC1s toward IFNβ- and/or CD40L-deficient CD4^+^ T cells cultured at a 4:1 E:T ratio. As before, Granzyme B production was significantly decreased in CD8^+^ T cells cultured with IFNβ- and/or CD40L-deficient CD4^+^ T cells compared with those cultured with WT CD4^+^ T cells. Additionally, surface expression of CD107α, which indicates the release of cytotoxic granules [34], was significantly decreased on CD8^+^ T cells cultured with IFNβ single-deficient and IFNβ/CD40L double-deficient CD4^+^ T cells (Fig. 6B, C). Importantly, MART-1-specific CD8^+^ T cells cultured with IFNβ/CD40L double-deficient CD4^+^ T cells were the least capable of killing Mel526 cells, as indicated by the numbers of total and dead Mel526 cells in suspension (Fig. 6D, E; Supplementary Fig. 9D, E), the quantification of Caspase 3/7 activity (Fig. 6F), and the confluence of the remaining Mel526 cells (Fig. 6G, H). Taken together, our findings demonstrated that the combined loss of IFNβ and CD40L in activated CD4^+^ T cells further inhibited cDC1-mediated CTL cross-priming. We concluded that in humans, cDC1 licensing for CTL priming via IFN-I signaling is CD40 independent and that both CD40 and IFN-I signaling are required for optimal cDC1-mediated CTL priming.

### Tumor-infiltrating Ki67^+^CXCL13^+^CD4^+^ T cells produce IFN-I in multiple tumor types

**Fig. 7:**
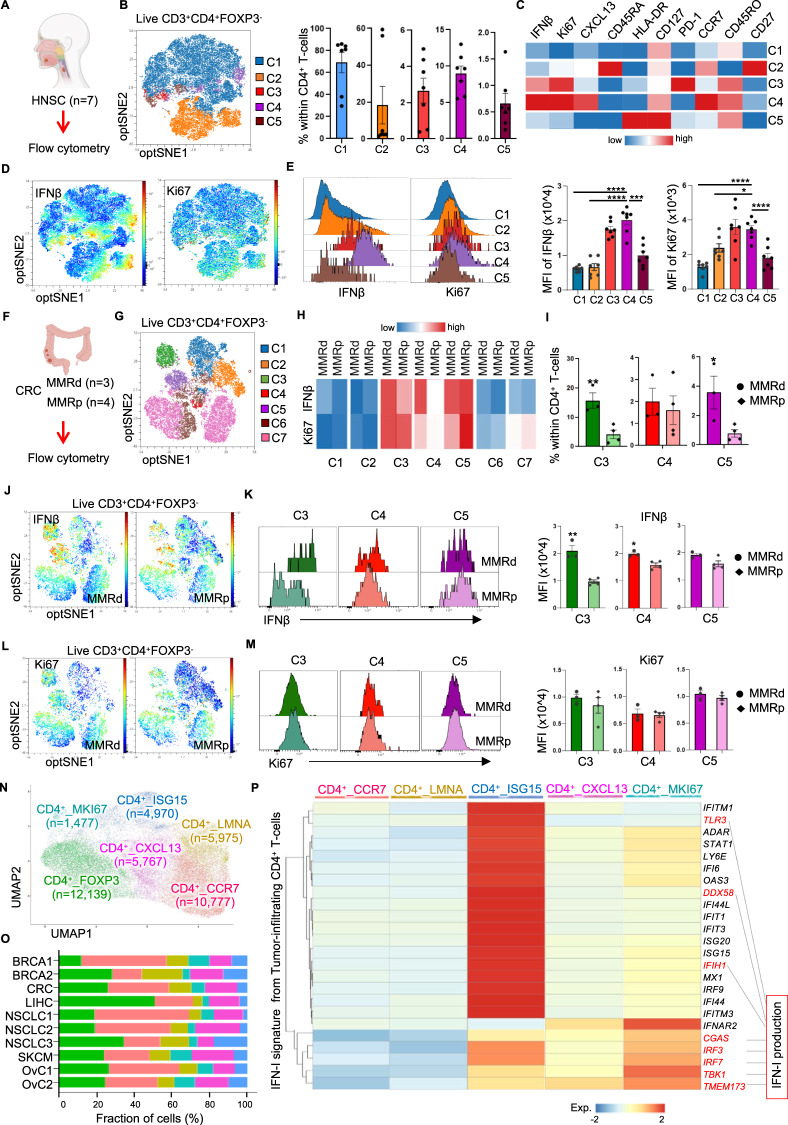
Tumor-infiltrating Ki67^+^CXCL13^+^CD4^+^ T cells express IFN-I across multiple tumor types. **A** Schematic illustration of the experimental procedure using head and neck cancer samples (HNSC, *n* = 7). **B** OptSNE plot of the five clusters identified and the percentage of each cluster among tumor-infiltrating FOXP3^−^CD4^+^ T cells. **C** Heatmap depicting the median expression values of the indicated markers in the five CD4^+^ T-cell clusters. **D** OptSNE plots, **E** histograms and MFI values for the expression of IFNβ and Ki67 in the five CD4^+^ T-cell clusters. **F** Schematic illustration of the experimental procedure using mismatch repair-deficient (MMRd, n = 3) and MMR-proficient (MMRp, *n* = 4) colorectal cancer (CRC) samples. **G** OptSNE plot of the seven clusters identified among tumor-infiltrating FOXP3^-^CD4^+^ T cells. **H** Heatmap depicting the median expression values of IFNβ and Ki67 in the seven CD4^+^ T-cell clusters. **I** Percentages of cluster 3-5 cells among CD4^+^ T-cells. OptSNE plots of (**J**) IFNβ and (**L**) Ki67 expression in FOXP3^-^CD4^+^ T cells. Histograms and MFI values for (**K**) IFNβ and (**M**) Ki67 expression in clusters 3-5. **N** UMAP plot of the tumor-infiltrating CD4^+^ T-cell clusters identified across ten scRNA-seq studies. **O** Compositions of the tumor-infiltrating CD4^+^ T-cell clusters identified in the ten scRNA-seq studies. The color scheme is shown in (**N**). **P** Heatmap of scRNA-seq data depicting the average expression values of the IFN-I signature [37] in CD4^+^ T cells across multiple tumor types. IFN-I production-related genes are indicated. *P* < 0.05*, *P* < 0.01**, *P* < 0.001***, *P* < 0.0001**** (one-way ANOVA for **E**; two-tailed Mann‒Whitney *U*-test for **I**, **K**, **M**). The data are shown as the means ± SEMs

We next explored the involvement of IFN-I-producing CD4^+^ T cells in cDC1 licensing in the TME. We first investigated whether tumor-infiltrating CD4^+^ T cells express IFN-I by multispectral flow cytometry on head and neck cancer (HNSC) (Fig. 7A) and colorectal cancer (CRC) (Fig. 7F, Supplementary Fig. 10A) tissue digests. Based on the expression of the optSNE-embedded markers, subclusters were identified and quantified within live CD3^+^ FOXP3^-^ CD4^+^ T cells (Fig. 7B, G; Supplementary Fig. 10B). IFNβ was detected in CD4^+^ T-cell clusters 3 and 4 from HNSC tissues (Fig. 7C–E) and in clusters 3, 4 and 5 in CRC tissues **(**Fig. 7H, J–M, Supplementary Fig. 10D), all of which coexpressed the proliferation marker Ki67. Notably, CXCL13 and PD-1 were also detected in some of the IFNβ-expressing CD4^+^ T-cell clusters in both tumor types (Fig. 7C; Supplementary Fig. 10D). The CD45RA^-^ CD45RO^+^ cluster 4 cells in HNSC tissues (Fig. 7C, Supplementary Fig. 10b) and clusters 3 and 5 in CRC tissues (Supplementary Fig. 10D, E) expressed CCR7, indicating a central memory T-cell phenotype. When mismatch repair-deficient (MMRd) and MMR-proficient (MMRp) CRC samples were compared, IFNβ expression in cluster 3 and cluster 4 CD4^+^ T cells (positive for CXCL13) was found to be significantly greater in the MMRd tumor samples (Fig. 7J, K and Supplementary Fig. 10D). Notably, MMRd cancers are notorious for increased immunogenicity as a result of their high mutation burden [35]. Interestingly, PD-1, CD27 and CD39 were detected at higher levels in cluster 3 cells (Supplementary Fig. 10D, E), suggesting that they are reactive to antigens in situ [36]. More tumor-infiltrating CD4^+^ T cells exhibited the cluster 3 and cluster 5 phenotypes in MMRd tumors than in MMRp tumors (Fig. 7I).

scRNAseq data generated from eight breast cancer patients by Azizi et al. [37] showed that CD4^+^ effector and central memory clusters exhibit an IFN-I gene signature in the TME. To better identify and characterize tumor-infiltrating IFNβ-producing CD4^+^ T-cell populations, we reanalyzed the single-cell data of Azizi et al. by comparing effector (memory) CD4^+^ T cells and naive CD4^+^ T cells (Supplementary Fig. 11A) isolated from primary tumor tissues. An enriched IFN-I signature, which included both IFN-I response-related and IFN-I production-related genes, was identified in tumor-infiltrating effector (memory) CD4^+^ T cells (Supplementary Fig. 11B) (Supplementary Table 3). Next, we compiled a cohort of ten scRNAseq datasets across six types of human cancer to confirm the presence of IFN-I-producing CD4^+^ T cells in the TME (Supplementary Fig. 11C). Six tumor-infiltrating CD4^+^ T-cell clusters (CD4^+^_FOXP3, CD4^+^_CCR7, CD4^+^_LMNA, CD4^+^_ISG15, CD4^+^_CXCL13 and CD4^+^_MKI67) were identified after data filtering, integration, unsupervised clustering and annotation (Fig. 7N, O; Supplementary Fig. 11D; Supplementary Table 4). Ordering of the identified CD4^+^ T-cell clusters by diffusion pseudotime [38] relative to CD4^+^_CCR7 (naive CD4^+^ T cells) indicated that the CD4^+^_LMNA and CD4^+^_CXCL13 clusters were in the middle of the differentiation trajectory and that the CD4^+^_FOXP3, CD4^+^_ISG15 and CD4^+^_MKI67 clusters were more differentiated populations (Supplementary Fig. 11E).

Meta-analysis of conventional (FOXP3^-^) CD4^+^ T-cell populations indicated that genes in the IFN-I signature (Supplementary Fig. 11B; Supplementary Table 3) were more highly expressed in the CD4^+^_ISG15, CD4^+^_CXCL13 and CD4^+^_MKI67 clusters than in the CD4^+^_CCR7 and CD4^+^_LMNA clusters (Fig. 7P). Specifically, the CD4^+^_ISG15 cluster had the highest expression of genes indicating an IFN-I-induced response, whereas the CD4^+^_MKI67 cluster had the highest expression of genes indicating IFN-I production (Fig. 7P). This finding was in agreement with the correlation between IFN-I and Ki67 expression detected in HNSC (Fig. 7C–E) and CRC tumors (Fig. 7H, J–M; Supplementary Fig. 10D). In addition, the CD4^+^_ISG15, CD4^+^_MKI67 and IFN-I signatures identified in tumor-infiltrating CD4^+^ T cells (Supplementary Table 3**)** showed enrichment in the same tissue region in a human breast cancer specimen subjected to spatial transcriptomic profiling (10x Visium) (Supplementary Fig. 11F and G). Importantly, a gene expression signature that indicates tumor reactivity in tumor-infiltrating lymphocytes (Supplementary Table 5), as identified by Lowery et al. [39], was enriched in the CD4^+^_CXCL13 and CD4^+^_MKI67 clusters (Supplementary Fig. 11H). This finding indicates that the CD4^+^ T cells with an IFN-I production profile identified here in the TME of six types of human cancer are likely specific for tumor antigens. The IFN-I gene signature identified in tumor-infiltrating CD4^+^ T cells was also enriched in in vitro-CD3/CD28-activated CD4^+^ T cells (Supplementary Fig. 11I). These results derived from both proteome and transcriptome datasets strongly suggest that tumor antigen-specific Ki67^+^CXCL13^+^CD4^+^ T cells [40, 41] in the TME produce IFN-I.

### Tumor-infiltrating Ki67^+^CXCL13^+^CD4^+^ T cells with enrichment of the IFN-I signature are associated with better clinical outcomes in cancer patients

**Fig. 8:**
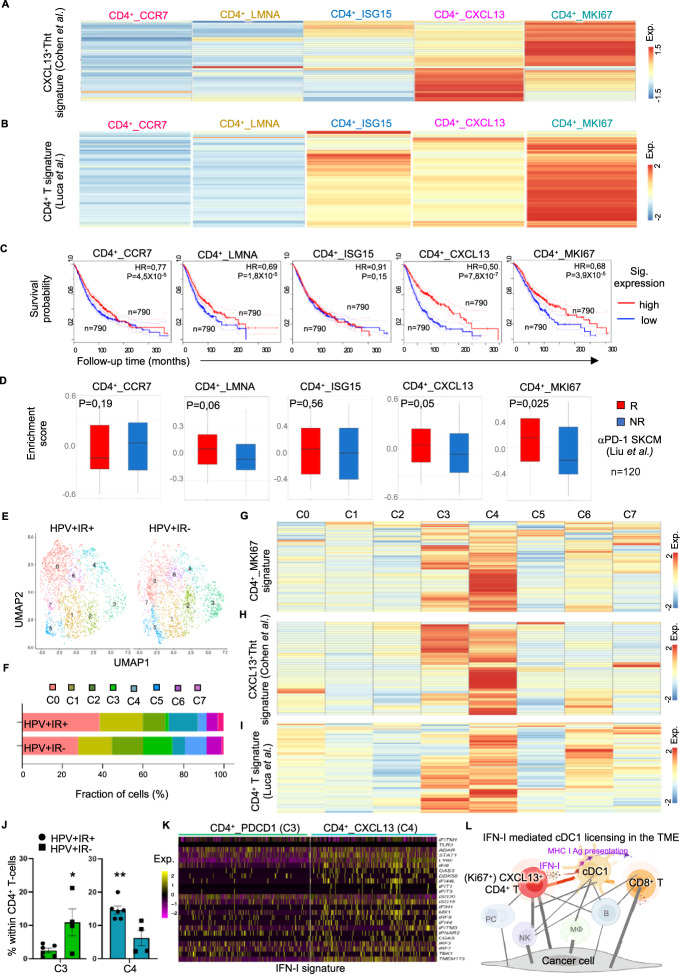
Tumor-infiltrating Ki67^+^CXCL13^+^CD4^+^ T cells enriched with the IFN-I signature are associated with better clinical outcomes in cancer patients. Tumor-infiltrating Ki67^+^CXCL13^+^CD4^+^ T cells enriched with the IFN-I signature are associated with better clinical outcomes in cancer patients. **A**, **B** Heatmaps depicting the average expression values of signature genes of the **A** CXCL13^+^ T-helper tumor-specific cell (Tht) signature [40] and the **B** tumor-infiltrating CD4^+^ T cells in CE9 [19] in the five tumor-infiltrating FOXP3^-^CD4^+^ T-cell clusters that we identified. **C** Kaplan–Meier curves revealing the prognostic value of the tumor-infiltrating CD4^+^ T-cell clusters that we identified for overall patient survival in the BRCA, COAD, LIHC, LUAD, OV, READ and SKCM cohorts from the TCGA database (*n* = 3156). The high and low metagene expression subgroups of patients were identified based on a threshold of the quartile expression level. **D** Box plots depicting the enrichment scores of tumor-infiltrating CD4^+^ T-cell clusters in responders (R) and nonresponders (NR) to anti-PD-1 immunotherapy [42]. **E**–**K** ScRNAseq data of tumor-infiltrating FOXP3^−^CD4^+^ T cells from HPV^+^ OPSCC patients (*n* = 43) were analyzed. The patients were stratified into the immune response-positive (IR^+^, *n* = 6) and immune response-negative (IR^−^, *n* = 4) groups based on the presence or absence, respectively, of tumor-specific T-cell infiltration. **E**, **F** UMAP plot and compositions of the eight clusters identified in the IR^+^ and IR^−^ groups. Heatmaps depicting the average expression values of **G** the tumor-infiltrating CD4^+^_MKI67 signature that we identified, **H** the CXCL13^+^ Tht signature [40] and (**I**) the tumor-infiltrating CD4^+^ T-cell signature in CE9 [19] in each cluster. **J** Percentages of cluster 3 and 4 cells among CD4^+^ T-cells from patients in the IR^+^ and IR^−^ groups. **K** Heatmaps depicting the expression of the IFN-I signature in tumor-infiltrating CD4^+^ T cells in clusters 3 and 4. **L** Schematic illustration depicting the mechanism of cDC1 licensing mediated by IFN-I-producing CXCL13^+^CD4^+^ T cells in the proinflammatory TME (CE9) [19]. *P* < 0.05*, *P* < 0.01** (log-rank test/Mantel–Cox test in C; Mann–Whitney *U*-test for **J**). The data are shown as the means ± SEM

Cohen et al. [40] recently reported a tumor-specific CXCL13^+^ CD4^+^ T-helper cell (Tht) population in the TME (Supplementary Table 5) that constitutes the major interacting hub with LAMP3^+^ DCs. We found that the gene expression signature of LAMP3^+^ DCs is part of the “helped” cDC1 signature [6], suggesting that the cell‒cell interaction described by Cohen et al. reflects a “help” scenario in the TME. We therefore investigated the relationship between the CXCL13^+^ Tht population identified by Cohen et al. [39] and the IFN-I-producing Ki67^+^CXCL13^+^CD4^+^ T cell population identified by us (Fig. 7P). Interestingly, the CXCL13^+^ Tht signature identified by Cohen et al. [40] was enriched in the CD4^+^_CXCL13 and CD4^+^_MKI67 clusters (Fig. 8A).

Luca et al. [19] defined three clinically favorable CEs among 16 tumor types. Among these, CE9 is characterized by the combined presence of DCs, effector CD4^+^ T cells and effector CD8^+^ T cells. We previously reported that the tumor-infiltrating DC signature in CE9 shares many features with the “helped” cDC1 signature [6]. To investigate the relationship between the CD4^+^ T cells present in CE9 (Supplementary Table 5) and the CD4^+^ T-cell populations we identified (Supplementary Fig. 11D, Supplementary Table 4), we performed signature cross-comparisons. The gene expression signature of the CD4^+^ T cells present in CE9 proved to be highly enriched in our identified CD4^+^_MKI67 population (Fig. 8B). Furthermore, the signatures of the CD4^+^_MKI67 population, the CXCL13^+^ Tht population identified by Cohen et al. [40] and the CD4^+^ T-cell population in CE9 identified by Luca et al. [19] were all enriched in the same region in a human breast cancer specimen according to analysis of an in situ spatially barcoded microarray (Supplementary Figs. 11F and 12A). The “helped/licensed” cDC1 [6] signature we previously identified was also enriched in the same tissue region (Supplementary Fig. 12A). We also analyzed CD8^+^ T-cell populations in the TME in the abovementioned ten scRNAseq datasets (Supplementary Fig. 13A, B). The tumor-reactive T-cell signature identified by Lowery et al. [39] (Supplementary Table 5) was enriched in CD8^+^_CXCL13 (cluster 0) and CD8^+^_MKI67 (cluster 8) (Supplementary Fig. 13C; Supplementary Table 6). The frequencies of the tumor-reactive CD8^+^_CXCL13 and CD8^+^_KI67 populations were significantly positively correlated with the frequency of the IFN-producing CD4^+^_MKI67 population (Supplementary Fig. 13D), supporting the concept of a tumor antigen-driven “help” scenario in the TME.

To assess the clinical relevance of our findings, we performed Kaplan‒Meier survival analysis of the BRCA, COAD, LIHC, LUAD, OV, READ and SKCM cohorts from the TCGA database. The prognostic value of the CD4^+^_CXCL13 (HR = 0.50) and CD4^+^_MKI67 signatures was significantly greater than that of the other CD4^+^ T-cell populations (HR = 0.68) (Fig. 8C). We also analyzed the clinical predictive value of the tumor-infiltrating CD4^+^ T-cell populations for the response to cancer therapies. For this purpose, we used a melanoma cohort receiving PD-1 blockade therapy [42] and an HPV^+^ oropharyngeal squamous cell carcinoma (OPSCC) cohort [43] receiving standard-of-care therapy consisting of surgery, radiotherapy, chemotherapy or combinations of these modalities. In the melanoma cohort, PD-1 blockade responders (R) had significantly higher enrichment scores for the CD4^+^_MKI67 signature (*p* = 0.025) and the CD4^+^_CXCL13 signature (*p* = 0.05) than nonresponders (NR) did (Fig. 8D), indicating the predictive value of this cell population. In the OPSCC cohort, patients were divided into the immune response-positive (IR^+^) and immune response-negative (IR^−^) groups based on the presence or absence, respectively, of tumor-specific T cells among the tumor-infiltrating lymphocytes, and IR^+^ patients had excellent survival during the >10 years of follow-up [43]. After standard scRNAseq data analysis, eight clusters were identified within tumor-infiltrating FOXP3^−^CD4^+^ T cells (Fig. 8E, F; Supplementary Fig. 12B; Supplementary Table 7), and seven clusters were identified within tumor-infiltrating CD8^+^ T cells (Supplementary Fig. 13E, F; Supplementary Table 7). The tumor-reactive T-cell signature identified by Lowery et al. [39] (Supplementary Table 5) was enriched in the CD8+_CXCL13 population (Supplementary Fig. 13G) and was significantly increased in patients in the IR^+^ group (Supplementary Fig. 13H). Among the eight clusters of tumor-infiltrating FOXP3^-^CD4^+^ T cells, the signatures of the tumor-infiltrating CD4^+^_MKI67 cells identified by us (Fig. 8G), the CXCL13^+^ Tht cells identified by Cohen et al. [39] (Fig. 8H) and the tumor-infiltrating CD4^+^ T cells in CE9 identified by Luca et al. [19] (Fig. 8I) were highly enriched in the CD4^+^_CXCL13 (cluster 4) population and, to a lesser extent, in the CD4^+^_PDCD1 (cluster 3) population (Fig. 8G–I, Supplementary Fig. 12B). Additionally, the CD4^+^_CXCL13 (cluster 4) population was significantly more represented in the IR^+^ group, and the CD4^+^_PDCD1 (cluster 3) population was more represented in the IR^-^ group (Fig. 8J), indicating that the CD4^+^_CXCL13 population was associated with a better response to treatments. The frequencies of other CD4^+^ T-cell populations were similar between the IR^+^ and IR^-^ groups (Supplementary Fig. 12C). Importantly, the CD4^+^_CXCL13 (cluster 4) population had higher expression of IFN-I signature genes than did the CD4^+^_PDCD1 population (Fig. 8K).

In summary, our findings suggest that IFN-I signature enrichment is a distinguishing feature of tumor-infiltrating, tumor-reactive Ki67^+^ CXCL13^+^CD4^+^ T cells that license cDC1s for the induction of antitumor CTL activity in the TME, which potentially leads to a favorable clinical outcome (Fig. 8L).

## Discussion

This study revealed that activated CD4^+^ T cells optimize cDC1s for CTL cross-priming to tumor cell-associated antigens by providing not only CD40L but also IFN-I, which act in a nonredundant manner. Our findings support and advance the observation from a recent study reporting the combined effect of IFNβ and CD40L in improving the functions of cDCs in antitumor immunity [44]. In general, IFN-I production is associated with myeloid cell types rather than T cells. In particular, pDCs produce massive amounts of IFN-I [45]. In cancer, IFN-I impacts tumor cells themselves and immune cell–tumor cell crosstalk, and the outcome depends on many factors [46, 47], including the source, niche and duration of IFN-I signaling. The reported sources of IFN-I are DCs [24] and tumor cells [48, 49], and STING pathway activation upon the sensing of DAMPs is the key pathway of IFN-I induction. IFN-I can have many positive effects on antitumor immunity, including increasing the presentation of tumor-associated antigens [50], suppressing the activity of protumorigenic Tregs and/or myeloid-derived suppressor cells [51], and improving effector CD8^+^ T-cell and Th1 function [29, 52].

IFN-I production by T cells has been associated with spontaneous resistance to HIV infection [53] and the antitumor activity of chimeric antigen receptor T-cell therapy [54], but lymphocytes are not generally considered a major source of IFN-I. A recent study revealed that the response to STING stimulation is more intricately controlled in T cells than in myeloid cells [55]. First, the NF-κB subunit RELA needs to be posttranslationally modified to respond to STING pathway activation, and IRF3 upregulation and DNA demethylation are additionally required to allow T cells to produce IFN-I at similar levels as myeloid cells. In the present study, all these events were accomplished by genetic and drug-based interventions; therefore, the exact regulatory mechanisms that restrict or enable STING-induced IFN-I production in T cells are not yet known. We detected STING pathway activation and IFNβ production in CD4^+^ T cells upon prolonged combined stimulation with CD3 and CD28 but not after stimulation with CD3 or CD28 alone. This condition mimics T-cell activation via the TCR/CD3 complex and CD28 costimulation, which occurs via dialog between an antigen-specific T-cell and an activated, antigen-presenting DC in either the lymph node or the tumor. CD3/CD28 stimulation can lead to mitochondrial stress [56] or to the enrichment of genomic DNA in the cytosol in T cells [57], events that may lead to the synthesis of cGAMP, which can activate the STING pathway or be imported into neighboring cells [58]. In addition, CD28 offers a specific, as-yet-unknown signal that contributes to RELA modification and/or other signaling aspects required for IFN-I production, as described in Jeremiah et al. [55]. The requirement for CD28 costimulation is in line with the proposition of Jeremiah et al. that IFN-I production by T cells is tightly controlled to avoid the accumulation of pathogenic amounts of IFN-I since T cells constitute a much larger proportion of immune cells than DCs. Our data suggest that IFN-I is delivered during antigen dependent, synaptic communication between a CD4^+^ T cell and a cDC1, wherein IFN-I would benefit only these two cell types and not spill into the environment. We have described the similar transient and synaptic delivery of CD70 by DCs to CD4^+^ T cells upon specific antigen recognition [59]. The provision of IFN-I by T cells in the context of cancer may be particularly important because STING signaling is suppressed in many tumors due to loss-of-function mutations or epigenetic silencing of the STING/cGAS promoter regions [60], and pDCs are reportedly defective in IFN-I production and physically colocalize with Tregs in the TME [61, 62].

DC maturation, characterized by the upregulation of costimulatory ligands, production of specific cytokines and optimized antigen presentation, can be achieved via PRR or CD40 signaling [1, 2]. CD4^+^ T cells express CD40L upon TCR-mediated activation, and DC-mediated CD4^+^ T-cell help to support the CD8^+^ T-cell response was shown in pioneering studies to be CD40 dependent [7, 8]. Recent work has shown that CD40 signaling into cDC1s is essential for the CTL response to implanted tumors in mice [5]. The effects of CD40 signaling are in part due to the induction of CD70, which leads to CD27 costimulation of CD8^+^ T cells [9, 10] and upregulation of Bcl-xL, ultimately leading to increased survival of cDC1s [11]. Here, we show that the key role of CD40L on activated CD4^+^ T cells in cDC1 licensing is conserved in humans. In a seemingly redundant manner, both IFN-I and CD40L in human CD4^+^ T cells upregulate costimulatory molecules, including CD70, the chemokine receptor CCR7 and the chemokines CXCL9/10, in cDC1s. We identify IFN-I signaling as a CD40L-independent pathway and demonstrate the complementarity of the two signals in the impact of licensed cDC1s on CTL priming. Gressier et al. [63] recently compared the impact of the “innate” stimulus IFN-I and the adaptive CD4^+^ T-cell-derived CD40L on gene expression in mouse bone marrow-derived DCs. The motivation of this investigation was that both signals may be independently delivered during viral infection. Gressier et al. demonstrated that CCL4, CCL5 and CXCL16 require both IFN-I and CD40 signaling for optimal cDC1 licensing and showed that activation of CD40 signaling is in part dependent on IFN-I stimulation, which exerts regulatory control over downstream molecules in the CD40L-CD40 axis, including p65, p38 and ERK [63]. The novelty of our study lies in revealing that the IFN-I signal originates from CD4^+^ T cells and is also a component of the cDC1 licensing program, in addition to the CD40 ligand signal. We showed that this complementarity is likely due, at least in part, to the unique impact of IFN-I on the optimization of the MHC class I antigen presentation pathway. Greyer et al. [14] also reported that the induction of IL-15 expression in DCs is not CD40 dependent but requires IFN-I stimulation. Zheng et al. [44] recently examined the effects of adenovirus-based delivery of IFN-I and CD40L on cDCs in vivo and reported complementary effects. They specifically noted the activation and migration properties of tumor-infiltrating cDC1s, which lead to potent induction of systemic CD8^+^ T-cell immunity and control of tumor growth. Specifically, induction of CCR7 expression on cDC1s endows them with the ability to migrate to (tumor-draining) lymph nodes (tdLNs), and this is the mechanism by which CD4^+^ T-cell help delivered in the TME can lead to the availability of “helped/licensed” cDC1s bearing tumor antigens in tdLNs, which can help perpetuate the cancer immunity cycle [64].

In our previous study, we found that cDC1s exhibit a “helped/licensed” phenotype in the TME of multiple tumor types according to the gene expression signature we defined in vitro [6]. The common characteristic of these tumors is that they have a CE9 constellation, as defined by Luca et al. [19]. In these T-cell-infiltrated tumors, CD4^+^ T cells, DCs (in the DC_S3 state) and CD8^+^ T cells coexist, and the CE9 constellation correlates with longer OS times and better responsiveness to immune checkpoint blockade (ICB) therapy. The DC_S3 state was proven to be enriched for the cDC1 “help” signature; accordingly, the cDC1 “help” signature was also correlated with longer OS times and better responsiveness to ICB therapy [6]. On the basis of these findings, we propose that in T-cell-infiltrated tumors, CD4^+^ T-cell help scenario plays out in the TME and is beneficial for CTL-based antitumor immunity. Here, across multiple tumor types, we identify IFNβ-expressing CD4^+^ T cells that have a Ki67^+^CXCL13^+^ and a partial PD-1^+^ phenotype. In a pancancer meta-analysis of 21 different tumor types, scRNAseq data [65] revealed intratumoral CD4^+^ T cells as two main populations: Tregs and conventional T cells that are of an undecided T follicular helper (Tfh)/Th1 phenotype [65]. The characteristics of Tfh cells include the expression of CXCL13, which is a B-cell attractant, and PD-1 [66]. Here, we find that the Ki67^+^ cells among the CXCL13^+^CD4^+^ T cells in the TME are the IFNβ-producing cells, are enriched in the tumor-reactive gene signature reported by Lowery et al. [39], and positively correlate with the presence of similarly defined tumor-reactive CXCL13^+^CD8^+^ T cells. The cell division activity and transcriptional profile of these cells suggest that they have been recently stimulated by antigen. This is plausible since CD4^+^ T cells of this phenotype have an oligoclonal repertoire and lie on a trajectory that culminates in CD39 expression, which is defined as a marker of tumor-antigen reactivity [65]. These data align with our concept that IFN-I is produced by CD4^+^ T cells as a result of antigen-specific interactions with cDC1s.

The IFN-I-producing CD4^+^ T-cell subset in the TME correlates with improvement in both OS and the response to PD-1-targeting ICB therapy. T-cell-infiltrated tumors do not spontaneously resolve; therefore, the cancer immunity cycle does not function optimally. Upon PD-1 blockade, CD28 costimulation is enabled in the dialog between cDC1s and T cells [67]. In mice, Cohen et al. [40] identified CD4^+^ T cells that physically interact with DCs in the TME and show a burst of IFN-I production (response) upon PD-1 blockade. In the treatment-naive lung cancer TME of PD-1 blockade responders, Chen et al. [68] identified spatially localized “stem-immunity hubs” that lack B cells, in contrast to TLSs, where cDC1-derived LAMP3^+^CCL19^+^IL12B^+^ DCs are most frequently adjacent to conventional CD4^+^ T cells and Tregs [68]. These data are in agreement with our proposed scenario wherein, in T-cell-infiltrated tumors, tumor antigen-specific CD4^+^ T cells and CD8^+^ T cells interact in a cognate manner with cDC1s and cDC1s are optimized to provide instructions to both cell types. Herein for CD4^+^ T cells to differentiate into Th1 cells and for CD8^+^ T cells to differentiate into optimal CTL effectors. It is known that tumors are populated with suboptimally primed CD8^+^ T cells that are in a TCF-1^+^PD-1^+^ stem-like state, and we propose that CD4^+^ T-cell help in the TME provides these cells with the required instructions for CTL effector differentiation. Possibly due to the co-presence of Tregs in the TME, this scenario does not play out optimally, and PD-1 blockade contributes to this process by further enabling it. Since tumors are complex and dynamic ecosystems [69], in-depth mapping of the cellular networks in the TME combined with spatial single-cell technology [70] will help to confirm these concepts.

## Materials and methods

### Human peripheral blood samples

Human PBMCs were obtained in accordance with the Declaration of Helsinki and the Dutch rules with respect to the use of human materials from volunteer donors. Buffy coats were obtained from healthy anonymized donors after their written informed consent, as approved by Sanquin’s internal ethics board. Human peripheral blood mononuclear cells (PBMCs) were isolated using Ficoll-Paque Plus (Cytiva) density gradient centrifugation (GE Healthcare), and the cells were cryopreserved until further use. DCs were isolated from HLA-A2^+^ donors, while the CD4^+^ and CD8^+^ T cells used in this study were used regardless of their HLA type and were not necessarily from the same donor.

### Patient samples

Seven head and neck squamous cell carcinoma (HNSC) patients [71] from the N16IMC trial (ClincalTrials.gov identifier: NCT03003637) along with 4 MMRp and 3 MMRd colorectal cancer (CRC) patients were included in this study. All patients were treatment naive when the samples were obtained (Supplemental Table 8). Tumor tissues from HNSC patients were cut into pieces of approximately 1–2 mm^3^ and cryopreserved until flow cytometric analysis. Tumor tissues from CRC patients were cut into small fragments in a Petri dish and enzymatically digested with 1 mg/mL collagenase D (Roche) and 100 μg/mL DNase I (Merck) in 5 mL of Iscove’s modified Dulbecco’s medium (IMDM; Gibco) supplemented with fetal bovine serum (FBS; Sigma) for 30 min at 37 °C in gentleMACS C tubes (Miltenyi Biotec). During and after incubation, the cell suspensions were mechanically dissociated in a gentleMACS Dissociator (Miltenyi Biotec). The cell suspensions were filtered through 70-μm cell strainers (Corning), washed in IMDM, and cryopreserved until further use. For flow cytometric analysis, tumor pieces from HNSC patients were first enzymatically digested with Liberase TL research grade (250 mg/ml; Merck) for 20 min at 37 °C, passed through 70-μm cell strainers (Falcon) and centrifuged at 400 × *g* for 5 min. After removal of the supernatant, the pelleted cells were resuspended in red blood cell lysis buffer and incubated at RT for 2 min. For samples from CRC patients, single-cell suspensions were quickly thawed and washed in RPMI-1640 medium + 20% FBS. Cells from HNSC and CRC patients were incubated with BD GolgiPlug (1:1000, BD Biosciences) for 5 h before staining. For staining, cells were first incubated in DNase I (0.1 mg/ml) at RT for 10 min, stained for surface markers, and fixed and permeabilized with a FOXP3 Staining Buffer Set (Thermo Fisher Scientific) according to the manufacturer’s protocol. Finally, the cells were stained for intracellular markers before harvesting. The collection of patient samples was approved by the Medical Research Ethics Committee of the Netherlands Cancer Institute-Antoni van Leeuwenhoek Hospital (file# NL57794.031.16) and Leiden University Medical Center (protocol P15.282), and all patients provided written informed consent. All specimens were anonymized and handled according to the ethical guidelines described in the Code for Proper Secondary Use of Human Tissue in the Netherlands of the Dutch Federation of Medical Scientific Societies.

### Fluorescence-activated cell sorting (FACS)

For in vitro DC-T-cell coculture and NanoString nCounter gene expression analysis, PBMCs were directly used for FACS. CD19^+^ cells were depleted before sorting using CD19 magnetic MicroBeads (Miltenyi Biotec) according to the manufacturer’s protocol. Staining was performed at 4 °C for 45 min in flow cytometry staining buffer (BD Biosciences). Antibodies specific for the following proteins were used: from BioLegend, CD1c (PE-Cy7, clone L161), CD3 (BV510, clone OKT3), CD4 (FITC, clone OKT4), CD8 (Percp-cy5.5, clone SK1), CD11c (PE/AF700, clone Bu15/3.9), CD14 (ef450, clone M5E2), CD19 (BV510, clone HIB19), CD25 (PE, clone BC96), CD45RA (AF700, clone HI100), CD141 (BV421, clone M80), and HLA-DR (BV605, clone L243); from BD Biosciences, HLA-DR (APC-Cy7, clone G46-6); and from Miltenyi Biotec, CD141 (APC, clone REA674). A Near-IR Dead Cell Stain Kit (Invitrogen), a Zombie Red Fixable Viability Kit (BioLegend) or 7-aminoactinomycin D (7-AAD, eBioscience) was used to exclude dead cells. To prevent clumping of dead cells, DNase I (0.1 mg/ml) was added before sorting. Cell sorting was performed on a BD FACSAria III (BD Biosciences).

### Flow cytometry

Cell surface staining: Staining was performed at 4 °C for 30 min in flow cytometry staining buffer. Antibodies specific for the following proteins were used at a 1:50 dilution unless mentioned otherwise: from BioLegend, CD3 (BV650, clone OKT3), CD4 (BV785/PE-Cy7, clone OKT4), CD8 (AF700/BUV805, clone SK1), CD11c (APC/Fire750, clone 3.9), CD11c (AF700, clone Bu15), CD14 (Spark Blue550, clone 63D3), CD19 (BV510, clone HIB19), CD25 (BV785, clone M-A251), CD27 (APC/Fire810, clone O323), CD40 (BV711, clone 5C3), CD40L (PE-Cy7, clone SA047C3), CD44 (PE, clone C44Mab-5), CD45 (Spark UV387, clone HI30), CD45RA (Spark NIR685, clone HI100), CD45RO (BV570, clone UCHL1), CD69 (BV510, clone FN50), CD70 (Percp-cy5.5, clone 113-16), CD80 (BV785, clone 2D10), CD83 (BV421, clone HB15e), CD86 (BV510, clone IT2.2), CD127 (PE/Fire700, clone A019D5, 1:30), CD137 (BV711, clone 4B4-1), CD141 (BV421, clone M80), CCR7 (BV711/Percp-cy5.5, clone G043H7), CXCR3 (PE-Cy7, clone G025H7), HLA-A2 (PE-Cy7, clone BB7.2), HLA-ABC (FITC, clone W6/32), HLA-DR (PE/Fire810, clone L243), HLA-DR (APC-Cy7, clone L243), HLA-DR.DP. DQ (PE-Cy7, clone Tu39), PD-L1 (BV711, clone 29E.2A3), and XCR1 (BV421, clone S15046E); from BD Biosciences, CD3 (BV480, clone UCHT1), CD27 (BV650, clone L128), CD45 (PE-CF594, clone HI30, 1:100), CD45RO (PE-CF594, clone UCHL1, 1:100), CD303 (BV650, clone V24-785), CD326/EPCAM (BUV737, clone EBA-1), PD-1 (PE-CF594, clone MIH4); from Miltenyi Biotec, CD141 (APC, clone REA674); and from ImmunoTools, CD8 (FITC, clone HIT8a). AF488- or APC-conjugated HLA-A2/MART-1_26-35_ tetramers were added together with cell surface staining antibodies. A Near-IR Dead Cell Stain Kit, a Zombie Red Fixable Viability Kit, a Zombie UV Fixable Viability Kit (BioLegend) or 7-aminoactinomycin D (7-AAD) were used to discriminate between live and dead cells.

For intracellular staining, a protein transport inhibitor (BD GolgiPlug) (1:1000) was added to the culture, which was incubated for 3 h before the cells were stained with cell surface markers. After surface staining, the cells were fixed and permeabilized using a BD Cytofix/Cytoperm Kit (BD Biosciences) or a FOXP3 Staining Buffer Set according to the manufacturer’s protocol. For phosphoprotein detection, cells were fixed with BD Cytofix™ Fixation Buffer (BD Biosciences) at room temperature for 20 min and permeabilized with BD™ Phosflow Perm Buffer III (BD Bioscience) at 4 °C for 30 min. Then, the cells were washed with flow cytometry staining buffer before intracellular staining. Antibodies specific for the following proteins were used at a 1:50 dilution unless mentioned otherwise: from BioLegend, CD107α (BV650, clone H4A3), β2m (PE, clone A17082A), Ki-67 (BV605, clone Ki-67, 1:30), Granzyme B (PE, clone QA16A02), CXCL9 (PE, clone J1015E10), CXCL10 (PE, clone J034D6), IFNγ (PE-Cy7, clone B27); from BD Biosciences, CD40L (PE-CF594, clone TRAP1, 1:100); from Miltenyi Biotec, pan-IFNα (APC, clone LT27:295); from R&D Systems, SLC19A1 (APC, clone mouse IgG_2A_ 890513, 1:30), ISG15 (PE, clone rat IgG_2A_); from Cell Signaling Technology, phospho-TBK1 (PE, clone D52C2, 1:100), phospho-IRF3 (AF647, clone E7J8G), phospho-STING (AF488, clone D8K6H); from Bioss, TAP1 and TAP2(rabbit polyclonal anti-human); and from Thermo Fisher Scientific, CXCL13 (APC, clone DSBCX13, 1:30), FOXP3 (PE-Cy5, clone PCH101), and IFNβ (AF488, clone A1), as well as goat anti-rabbit IgG(H+L) Alexa Fluor 488 (use 1:200) and goat anti-rabbit IgG(H+L) APC (use 1:300) as secondary antibodies. Specific staining was confirmed with the fluorescence minus one (FMO) control. The antibodies and dilutions used are listed in Supplementary Table 9. Flow cytometry was performed using a BD LSR Fortessa^TM^ (BD Biosciences) or Cytek Aurora spectral flow cytometer (Cytek Biosciences). The data were analyzed using FlowJo^TM^ software version 10.8.1 (BD Biosciences) or OMIQ software (Dotmatics).

### STING pathway stimulation/inhibition

Naive CD4^+^ T cells were flow sorted on the basis of the CD3^+^HLA-DR^-^CD4^+^CD25^-/low^CD45RA^+^ phenotype and cultured for 48-72 h after plating at 0.5 × 10^6^ cells/well in 96-well round bottom plates (BD Falcon) in RPMI-1640 medium supplemented with 10% FBS and Antibiotic–Antimycotic Solution (Sigma) in the presence of hIL-2, hIL-7 and hIL-15 (Miltenyi, each 10 ng/ml); additionally, monoclonal antibodies against CD3 and CD28 were added to activate CD4^+^ T cells. In addition, CD4^+^ T cells were treated with a STING inhibitor (H-151, 15 ng/ml; InvivoGen) or a STING agonist (2′3′-cGAMP, 15 μg/ml; InvivoGen) for the last 8 h of culture. Intracellular IFNβ and phosphoprotein detection was performed using flow cytometry.

### Enzyme-linked immunosorbent assay (ELISA)

Naive CD4^+^ T cells were plated at 0.5 × 10^6^ cells/well (200 μl/well) in a 96-well round bottom plate and cultured in medium containing 10 ng/ml hIL-2, hIL-7 and hIL-15 (Miltenyi). Additionally, monoclonal antibodies against CD3 (0.1 μg/ml, clone CLB-T3/4.E, Sanquin) and CD28 (0.2 μg/ml, clone CLB-CD28/1, Sanquin) were added to generate activated CD4^+^ T cells. The supernatant was collected after 48 h of culture and frozen at −20 °C until analysis. A Human IFN-Beta ELISA Kit with high sensitivity (PBL Assay Science) was used according to the manufacturer’s instructions.

### Immunoblotting

Naive CD4^+^ T cells were cultured in medium supplemented with 10 ng/ml hIL-2, hIL-7 and hIL-15 (Miltenyi). Additional monoclonal antibodies against CD3 (0.1 μg/ml, clone CLB-T3/4.E, Sanquin) and CD28 (0.2 μg/ml, clone CLB-CD28/1, Sanquin) were added to generate activated CD4^+^ T cells. During the last 8 h of culture, the CD4^+^ T cells were treated with a STING agonist (2′3′-cGAMP, 15 μg/ml). After 48 h of culture, T cells were collected, washed once with cold PBS and lysed on ice in RIPA lysis buffer (Pierce) supplemented with protease inhibitors (Roche), benzonase (Santa Cruz), and phosphatase inhibitor cocktail set V (Calbiochem). The lysates were cleared by centrifugation (15 min, 13,500 × *g* at 4 °C). THP-1 cells (ATCC, TIB-202), which were used as controls, were differentiated into macrophage-like cells by treatment with 150 nM phorbol 12-myristate 13-acetate (PMA; Sigma) for 24 h. Then, the PMA was removed by washing, and the cells were cultured for an additional 24 h before transfection. Next, THP-1-derived macrophages were plated at 0.3 × 10^6^ cells/well in 24-well plates (BD Falcon) and then transfected with G3-YSD (4 µg/well, InvivoGen) complexed with Lipofectamine 2000 (Thermo Fisher Scientific) at a 1:1 ratio for 6 h before being lysed in the same manner as described for T cells. Protein concentrations were assessed by a BCA Protein Assay Kit (Pierce, Thermo Fisher Scientific) and subsequently normalized. Proteins in the samples, along with Precision Plus Protein Dual Color Standards (Bio-Rad), were separated on 4–15% Mini-PROTEAN TGX precast gels (Bio-Rad) and transferred onto nitrocellulose membranes (Bio-Rad) by semidry transfer. The membranes were blocked in 10% sterile filtered BSA (Millipore) in TBS-T (20 mM Tris, pH 7.5; 150 mM NaCl; 0.1% Tween 20). The membranes were incubated with the relevant primary and secondary antibodies in Western BLoT Immuno Booster 1 or 2 solution (Takara Bio) and were then visualized on an Odyssey CLX-1391 imaging system (LI-COR Biosciences). The antibodies and dilutions used are listed in Supplementary Table 9.

### Ex vivo coculture of cDC1-CD4^+^ T cells

To generate activated CD4^+^ T cells, naive CD4^+^ T cells were first activated with monoclonal antibodies against CD3 (0.1 μg/ml, clone CLB-T3/4). E, Sanquin) and CD28 (0.2 μg/ml, clone CLB-CD28/1, Sanquin) for 48–72 h. Activated and nonactivated CD4^+^ T cells were cultured in the presence of 10 ng/ml hIL-2, hIL-7 and hIL-15 (Miltenyi). Ex vivo-generated cDC1s were purified by flow cytometry and were then cocultured with activated CD4^+^ T cells for 12 h. An anti-IFNAR2 monoclonal blocking antibody (clone MMHAR-3, 5 μg/ml; PBL Assay Science), an anti-IFNGR1 blocking antibody (5 μg/ml; R&D Systems), or mouse IgG2A isotype control (5 μg/ml; InvivoGen) were added accordingly. cDC1s cultured ex vivo without CD4^+^ T cells were used as controls.

### NanoString nCounter gene expression analysis

Ex vivo-cultured cDC1s were purified by flow cytometry and stimulated with universal IFNα (100 U/ml; PBL Assay Science) and IFNβ (150 pg/ml; R&D Systems), stimulated with IFNγ (10 ng/ml; InvivoGen) or left unstimulated for 12 h. An additional anti-CD40 blocking antibody (5 μg/ml; R&D Systems) was added 2 h before IFN stimulation as indicated. Then, the cells were lysed in buffer containing 1 volume of RLT buffer (QIAGEN) and 2 volumes of UltraPure™ DNase/RNase-free distilled water (Invitrogen) at a concentration of 2000 cells/μl buffer. The samples were analyzed on the NanoString nCounter® FLEX platform according to the manufacturer’s instructions. Briefly, 5 μl of lysate (10,000 cells) per condition from each donor was mixed with reporter probes, capture probes and hybridization buffer and subjected to hybridization at 65 °C for 20 h. Proteinase K (0.45 mg/ml; Thermo Fisher Scientific) was added during the hybridization step. The samples were subsequently processed on the NanoString Prep station, and the cartridges were read on the NanoString Digital Analyzer. The human host response panel (785 genes) was used. The RNA count data were normalized, scores for different pathways were calculated, and automated pathway analysis based on the expression of predefined genes was performed using nSolver software (advanced analysis module version 1.1.4). The log_2_-transformed output data were subsequently analyzed in R (version 4.1.2), and the ‘*EnhancedVolcano*’ and ‘*ggplot2*’ packages were used to generate the volcano plot. A heatmap of the significantly differentially expressed genes (DEGs) was generated based on the criteria of an adjusted *p* value of <0.05 and a log_2_-fold change (FC) of >1 or <−1 using Qlucore Omics Explorer (version 3.8).

### siRNA transfection

Naive CD4^+^ T cells were first activated with monoclonal antibodies against CD3 (0.1 μg/ml; clone CLB-T3/4). E, Sanquin) and CD28 (0.2 μg/ml, clone CLB-CD28/1, Sanquin) for 36–48 h, after which the cells were washed twice with phosphate-buffered saline (PBS) before transfection. Transfection was performed in a 24-well plate. For transfection of the cells in each well, the *IFNB1* siRNA complex (9 pmol, Thermo Fisher Scientific) or negative control siRNA complex (6 pmol, Thermo Fisher Scientific) was diluted in 100 μl of Opti-MEM (Gibco) supplemented with 1% FBS in the 24-well tissue culture plate (BD Falcon). Then, 1.5 μl of Lipofectamine^TM^ RNAiMAX (Thermo Fisher Scientific) was added to each well containing the diluted siRNA molecules, mixed gently and incubated for 20 min at room temperature. Next, 50,000 cells in 500 μl of complete culture medium were added to each well and incubated for 24**–**36 h before the gene knockdown efficiency was assessed by real-time quantitative PCR or flow cytometry.

### Cas9/gRNA ribonucleoprotein (RNP) nucleofection

This method was adapted from a previously described protocol [72]. gRNAs in the proprietary Alt-R format (Integrated DNA Technologies), i.e., a two-component gRNA composed of a crRNA (Hs.Cas9.CD40LG.1.AA and Hs.Cas9.IFNB1.1.AB) annealed to a transactivating (tracer) RNA were used. A nontargeting control gRNA in the same format was used as a control. The gRNA (100 pmol) was then incubated with recombinant Cas9 to form the Cas9 RNP complex used for T-cell transfection. Before electroporation, 150 μl of RPMI-1640 medium (Gibco) supplemented with 10% FBS and hIL-7/hIL-15 (both 10 ng/ml; Miltenyi Biotec) was dispensed into each well of a 96-well round bottom plate, and the plate was incubated at 37 °C to warm the medium. Purified naive CD4^+^ T cells were washed with PBS twice, counted and resuspended at 1 × 10^6^ cells per 20 μl of P3 Primary Cell 4D-Nucleofector Buffer (Lonza) with Alt-R Cas9 Electroporation Enhancer (2 μM). Then, 5 μl of the RNP complex was added to the cell suspension and incubated for 2 min at room temperature. Next, the cells were transferred to Nucleocuvette strips (Lonza) and electroporated with pulse code FI115 in a 4D-Nucleofector Unit (Lonza). After nucleofection, the cells were immediately transferred to prewarmed medium at a density of 1 × 10^6^ cells/well. After 24 h, the T cells were activated with monoclonal antibodies against CD3 (0.1 μg/ml; clone CLB-T3/4). E, Sanquin) and CD28 (0.2 μg/ml, clone CLB-CD28/1, Sanquin), and 48–72 h later, the gene knockout efficiency was assessed via flow cytometry.

### Real-time quantitative PCR

Total RNA was extracted using TRIzol (Invitrogen) according to the manufacturer’s instructions. Five hundred nanograms of RNA was subsequently treated with ezDNase (Invitrogen) and reverse transcribed using SuperScript IV VILO Master Mix (Thermo Fisher Scientific) according to the manufacturer’s instructions. cDNA was diluted in nuclease-free water, and gene expression was measured with technical duplicates using PowerUp SYBR Green Master Mix (Thermo Fisher Scientific) on a QuantStudio 3 system (Thermo Fisher Scientific). The gene-specific primers used were as follows: *IFNB1* forward, AGTAGGCGACACTGTTCGTG; reverse, GTCTCATTCCAGCCAGTGCT. *ACTB*: forward: CACTCTTCCAGCCTTCCTTC, reverse: TACAGGTCTTTGCGGATGTC.

### Retroviral transduction of CD8^+^ T cells with MART-1_26-35_/HLA-A2-specific TCRs

This method was adapted from a previously described protocol [73]. Non-tissue culture-treated 24-well plates (BD Falcon) were coated with 10 ng/ml RetroNectin (Takara Bio) at 4 °C for 24 h, blocked with 2% bovine serum albumin (BSA; Sigma) for 30 min at room temperature (RT), and then washed with PBS twice. CD8^+^ T cells were cultured in RPMI-1640 medium supplemented with 10% FBS in the presence of human (h)IL-2, hIL-7 and hIL-15 (each 10 ng/ml) and human T-Activator CD3/CD28 Dynabeads (Thermo Fisher Scientific; 2 cells:1 bead) for 2–3 days before transduction. For transduction, CD8^+^ T cells were spun down and resuspended in retrovirus-containing medium from packaging cells supplemented with 10 ng/ml hIL-2/hIL-7/hIL-15 and plated at 0.5 × 10^6^ cells per well. The plates were centrifuged at 800 × *g* for 90 min at RT in a table-top centrifuge with an acceleration setting of 3 and a deceleration setting of 0. The cells were cultured for 24 h before the virus-containing supernatant was removed and were then expanded in medium supplemented with the cytokine cocktail and CD3/CD28 Dynabeads for 7 days. Next, the Dynabeads were removed, and the cells were incubated in medium supplemented with the cytokine cocktail for 3 days before being used in CTL priming experiments.

### Tumor antigen-specific CTL priming platform

This method was adapted from a protocol described in our previous publication [6]. To create conditions for antigen cross-presentation, MART-1_15-40_ long peptide (KGHGHSYTTAEELAGIGILTV) or dead Mel526 cells were used. Mel526 cells were treated with 100 ng/ml tumor necrosis factor-related apoptosis-inducing ligand (TRAIL; Sigma) and 10 ng/ml Fas Ligand (FASL; AdipoGen) to induce apoptotic cell death. Flow sorted ex vivo HLA-A2^+^ cDC1s were incubated with or without activated CD4^+^ T cells for 2 h in IMDM supplemented with 1% FBS. Then, MART-1_15-40_ long peptide (20 mg/ml) or dead Mel526 cells were added. After 12–16 h, the cell supernatant was removed by washing. Then, MART-1_26-35_/HLA-A2-specific TCR-transduced CD8^+^ T cells were added to the culture at a ratio of 1 DC per 5-10 CD8^+^ T cells and cultured for 6-7 days in RPMI-1640 medium supplemented with 10% FBS and 0.2 ng/ml hIL-7/hIL-15. To trace proliferation, CD8^+^ T cells were labeled with CTV before being added to the CTL priming platform. Then, 50 ng/ml PMA, 1 mg/ml ionomycin (InvivoGen) and a protein transport inhibitor (BD GolgiPlug, 1:1000) were added to the culture, which was incubated for 3 h before the cells were harvested and analyzed via flow cytometry.

### IncuCyte T-cell cytotoxicity assay

One day before the cytotoxicity assay, live Mel526 cells were plated in 96-well flat bottom black wall plates (Greiner) at a density of 5000 cells/well. The Mel526 cells were passed through G21 needles (BD Biosciences) before plating to prevent aggregation. The following day, the growth medium was removed from the Mel526 cells, and 100 μl of IncuCyte® Caspase-3/7 Green Apoptosis Reagent (20 μM, Sartorius) was added. Then, CD8^+^ T cells isolated from the tumor antigen-specific CTL priming platform via flow sorting were seeded in 100 μl of medium into the appropriate wells at effector:target ratios of 0:1, 1:1, 2:1 and 4:1. The assay plate was allowed to settle on a level surface at ambient temperature for 30 min before being placed into the IncuCyte live-cell analysis system (IncuCyte ZOOM^®^, Sartorius). The plates were scanned every 2 h for 18 h. Afterward, the cell suspensions were collected and analyzed by flow cytometry, and the remaining Mel526 cells were fixed with 4% PFA (Sigma), stained with crystal violet and imaged with a Zeiss Axio Imager Z1 charge-coupled device (CCD) microscope. The IncuCyte data were analyzed with IncuCyte® S3 software (version 2018B). One-way repeated measures ANOVA in SPSS (IBM) was used to determine the significance of differences. A *P* value of <0.05 was considered to indicate statistical significance.

### Meta-analysis of single-cell (sc) mRNA sequencing data

(1) Preprocessed scRNA-seq data of immune cells from 8 primary breast tumors[37] were downloaded from the Gene Expression Omnibus (GEO). After importing the data into R (version 4.1.2) using the *‘Seurat’* package (version 4.0.0), variable features that exhibited high cell-to-cell variation in the dataset were identified using the *FindVariableFeatures* function. Scaling of the variable features was performed prior to dimensionality reduction using PCA. The clusters were identified using the *FindCluster* function in *Seurat*, and the nonlinear dimensionality reduction technique UMAP was used to visualize the cells in two-dimensional space. After clustering, effector-memory CD4^+^ T cells (CD4^+^ T__E(M)_) and naive CD4^+^ T cells (CD4^+^ T_naive) were selected for further analysis. The *FindAllMarkers* function in *Seurat* was used to identify DEGs. The genes that were detected in 25% of the cells of in the cluster and had a log_2_FC of > 0.5 between two groups of cells, with an adjusted P value of < 0.05, were considered to be significant DEGs. Gene set enrichment analysis (GSEA) was performed, and a tumor-infiltrating CD4^+^ T-cell-enriched IFN-1 signature was identified. (2) Preprocessed scRNA-seq data from 10 studies were downloaded from GEO or from EMBL-EBI (www.ebi.ac.uk/arrayexpress). The data were imported into R (version 4.1.2) using the *‘Seurat’* package (version 4.0.0). Datasets of CD4^+^ T cells and CD8^+^ T cells from primary tumors were first selected using the *Subset* function based on the annotations provided by the authors of those studies and were then integrated using the *Merge* function. Next, the integrated data were further filtered based on cells with between 200 and 5000 transcripts and with fewer than 5% mitochondrial gene events. After removing unwanted cells from the dataset, a standard workflow for integrated data from the ‘*Seurat’* package was employed. Final cluster annotation was manually performed based on the top 50 upregulated DEGs that were identified after cluster identification using the *FindCluster* function and differential expression analysis using the *FindAllMarkers* function. The nonlinear dimensionality technique UMAP was used to visualize the cells in two-dimensional space. The average expression of the IFN-I signature in tumor-infiltrating CD4^+^ T cells (Supplementary Table 3), the tumor-infiltrating CD4^+^ T-cell signature in CE9 [19] and the CXCL13^+^ Tht signature [40] (Supplementary Table 5) in tumor-infiltrating conventional CD4^+^ T cells were determined with the *AverageExpression* function. The ‘*ggplot2*’ package was subsequently used to generate a heatmap. The heatmap depicting the expression of the tumor-reactive T-cell signature [39] (Supplementary Table 5) in tumor-infiltrating CD4^+^ and CD8^+^ T cells was generated using the *DoHeatmap* function. The frequency of each cluster among tumor-infiltrating CD4^+^ or CD8^+^ T cells in each patient was determined in R. Spearman rank correlation coefficients were calculated using GraphPad Prism (version 9.0). (3) Trajectory inference and pseudotime calculations were performed using the ‘*Monocle 3*’ package [74] as part of the Seurat Wrappers workflow on the integrated scRNA-seq Seurat objects. First, the *as.cell_data_set* function was used to convert the Seurat object to a *Monocle 3* object; next, the *cluster_cells* function was used to group the cells into clusters/partitions; then, the *learn_graph* and *order_cells* functions were used to model the relationships between the clusters as a trajectory of gene expression changes. The CD4^+^_CCR7 cluster (white circle) was used as the *root_cells* node (beginning of the biological process). The black circles indicate branch nodes in which cells can travel to one of several outcomes, and the end of each black line end corresponds to a different outcome (i.e., cell fate) of the trajectory. (4) Preprocessed scRNA-seq data of immune cells from HPV^+^ oropharyngeal squamous cell carcinoma (OPSCC) patients were acquired in our previous study [43]. Patients were stratified into the immune response-positive (IR^+^, *n* = 6) and immune response-negative (IR^−^, *n* = 4) groups based on the presence or absence, respectively, of tumor-specific T-cell infiltration. The data were imported into R (version 4.1.2) using the *‘Seurat’* package (version 4.0.0). Datasets of tumor-infiltrating FOXP3^−^CD4^+^ T cells or CD8^+^ T cells were first selected using the *Subset* function based on the annotations in our previous study. Subsequently, variable features that exhibited high cell-to-cell variation in FOXP3^−^CD4^+^ T cells or CD8^+^ T cells were identified using the *FindVariableFeatures* function. Eight subclusters were identified within FOXP3^-^CD4^+^ T cells, and seven subclusters were identified within CD8^+^ T cells using the *FindCluster* function in *Seurat*, and the nonlinear dimensionality technique UMAP was used to visualize the cells. The average expression of the tumor-infiltrating CD4^+^_CXCL13 and CD4^+^_MKI67 signatures that we identified (Supplementary Table 4), as well as the tumor-infiltrating CD4^+^ T-cell signature in CE9 [19] and the CXCL13^+^ Tht signature [40] (Supplementary Table 5), in the FOXP3^−^CD4^+^ T-cell subclusters were obtained using the *AverageExpression* function. A heatmap depicting the expression of the IFN-I signature in tumor-infiltrating CD4^+^ T cells (Supplementary Table 3) in clusters 3 and 4 of FOXP3^-^CD4^+^ T cells and a heatmap depicting the expression of the tumor-reactive T-cell signature [39] (Supplementary Table 5) in CD8^+^ T cells were generated using the *DoHeatmap* function. The cell numbers per cluster in each patient were determined in R. The proportion of each cluster in each patient was then calculated, and statistical analysis was performed using GraphPad Prism (version 9.0).

### Gene set enrichment analysis (GSEA)

The Log_2_FC values of the 577 DEGs between cDC1s cultured with activated CD4^+^ T cells and cDC1s cultured with naive CD4^+^ T cells identified by our scRNA-seq analysis or the log_2_FC values of the 542 DEGs between tumor-infiltrating CD4^+^ effector-memory T cells and naive CD4^+^ T cells identified in the scRNA-seq data of 8 primary breast tumors [37] were used for GSEA. GSEA software (version 4.2.2) (http://broadinstitute.org/gsea) and the Reactome pathway database (https://www.gsea-msigdb.org/gsea/msigdb/index.jsp) were used with default parameters to calculate enrichment and generate GSEA plots.

### Spatial transcriptome analysis

Preprocessed spatial transcriptomics datasets of breast carcinoma specimens were downloaded from 10x Genomics (https://www.10xgenomics.com/spatial-transcriptomics/). After the data files were uploaded into Cell Loupe browser software 5.0, the Gene/Feature Expression mode was chosen, and the data were scaled according to the average log_2_Feature values. The IFN-I signature from tumor-infiltrating CD4^+^ T cells (Supplementary Table 3), the tumor-infiltrating CD4^+^ T_CCR7, CD4^+^ T_LMNA, CD4^+^ T_ISG15, CD4^+^ T_CXCL13 and CD4^+^ T_MKI67 signatures (Supplementary Table 4), the CXCL13^+^ Tht signature [40] (Supplementary Table 5), the tumor-infiltrating CD4^+^ T-cell signature in CE9 [19] (Supplementary Table 5), and the cDC1 “help” signature [6] were uploaded, and the distributions of the different signatures were visualized.

### Survival analysis in The Cancer Genome Atlas (TCGA) datasets

Survival analysis was carried out using the top 20 upregulated DEGs in the CD4^+^ T_CCR7, CD4^+^ T_LMNA, CD4^+^ T_ISG15, CD4^+^ T_CXCL13 and CD4^+^ T_MKI67 clusters (Supplementary Table 4). We assessed patient OS times and tumor gene expression profiles for combined datasets of BRCA, COAD, LIHC, LUAD, OV, READ and SKCM patients (*n* = 3156) in the TCGA database using the GEPIA2 computational workflow [75] based on the UCSC Xena platform (http://xena.ucsc.edu). Briefly, OS analysis was based on the log-rank hypothesis test (the Mantel–Cox statistical test), with estimation of Cox proportional hazard ratios (HRs) and 95% confidence intervals, accompanied by a Kaplan‒Meier (KM) plot. Herein, a threshold of the quartile signature expression level was used to divide the patients into the high-expression and low-expression subcohorts.

### Bulk mRNA analysis

The preprocessed RNA-seq TPM matrix was downloaded from the supplementary information of Liu et al. (https://www.nature.com/articles/s41591-019-0654-5#Sec31) and imported into R (version 4.1.2). Genes with a TPM of 0 in more than 25% of the samples were removed. After filtration, the ‘*GSVA*’ package [76] (version 4.2) was used to perform GSEA with the defined gene sets/signatures (Supplementary Table 4) in responders and nonresponders. Responders were defined as patients who achieved complete response (CR) or partial response (PR). The enrichment score for each patient (*n* = 120) was then used to calculate p values using the Wilcoxon signed-rank test. A *P* value of <0.05 was considered to indicate statistical significance.

### Statistical analysis

The data, excluding mRNA sequencing data obtained from public datasets and IncuCyte cytotoxicity assays, were analyzed using GraphPad Prism (version 9.0), and the Mann‒Whitney *U*-test or one-way ANOVA was used to determine the significance of differences among samples or groups. The data are presented as the means ±SEMs. A *P* value < 0.05 was considered to indicate statistical significance.

### Materials and data availability

This study did not generate new unique reagents. This paper analyzed existing, publicly available data. The accession numbers of the datasets are listed in the key resources table. The TCGA cohorts used in the survival analysis can be accessed via http://gepia2.cancer-pku.cn/#index. The Reactome database can be accessed via https://reactome.org/. All packages used to analyze sequencing data are publicly available and are listed in Supplementary Table 9. All the other primary data and materials that support the findings of this study are available from the corresponding author upon request.

### Supplementary information

revised suppl. material

revised suppl. table 1

revised suppl. table 2

revised suppl. table 3

revised suppl. table 4

revised suppl. table 5

revised suppl. table 6

revised suppl. table 7

revised suppl. table 8

revised suppl. table 9

source file for immunoblotting related to supplementary Figure 4D
