# Supplementary material for: CD4+ T cells produce IFN-I to license cDC1s for induction of cytotoxic T-cell activity in human tumors
Source: Cell Mol Immunol. 2024 Feb 21;21(4):374–92. doi: 10.1038/s41423-024-01133-1 (PMC10978876; doi:10.1038/s41423-024-01133-1)
Supplement: Supplementary file 1 — revised suppl. material [file 41423_2024_1133_MOESM1_ESM.pdf]

CD4+ T cells produce IFN-I to license cDC1s for induction of cytotoxic T-cell activity in human tumors

Xin Lei[1,2], Daniël C. de Groot[3], Marij J. P. Welters[4], Tom de Wit[1,2], Ellen Schrama[1,2], Hans van Eenennaam[5], Saskia J. Santegoets[4], Timo Oosenbrug[1], Annemarthe van der Veen[1], Joris L. Vos[6], Charlotte L. Zuur[6,7], Noel F. C. C. de Miranda[8], Heinz Jacobs[3], Sjoerd H. van der Burg[2,4], Jannie Borst[1,2]*, Yanling Xiao[1,2,9]*

[1]Department of Immunology, [2]Oncode Institute, Leiden University Medical Center, Leiden, The Netherlands. [3]Department of Tumor Biology and Immunology, The Netherlands Cancer Institute, Amsterdam, The Netherlands. [4]Department of Medical Oncology, Oncode Institute, Leiden University Medical Center, Leiden, The Netherlands. [5]IMMIOS B.V., Oss, The Netherlands. [6]Division of Medical Oncology, The Netherlands Cancer Institute, Amsterdam, The Netherlands. [7]Department of Otorhinolaryngology Leiden University Medical Center, Leiden, The Netherlands. [8]Department of Pathology, Leiden University Medical Center, Leiden, The Netherlands.

Running title: CD4+ T cells enable anti-tumor immunity via IFN-I

[9]Lead contact

*Correspondence: Yanling Xiao, email y.xiao@lumc.nl; Jannie Borst, email j.g.borst@lumc.nl; Phone: +31-71-5261970; postal address: Albinusdreef 2, 2333 ZA, Leiden, The Netherlands

Conflict of Interest

The authors have declared that no conflict of interest exists.

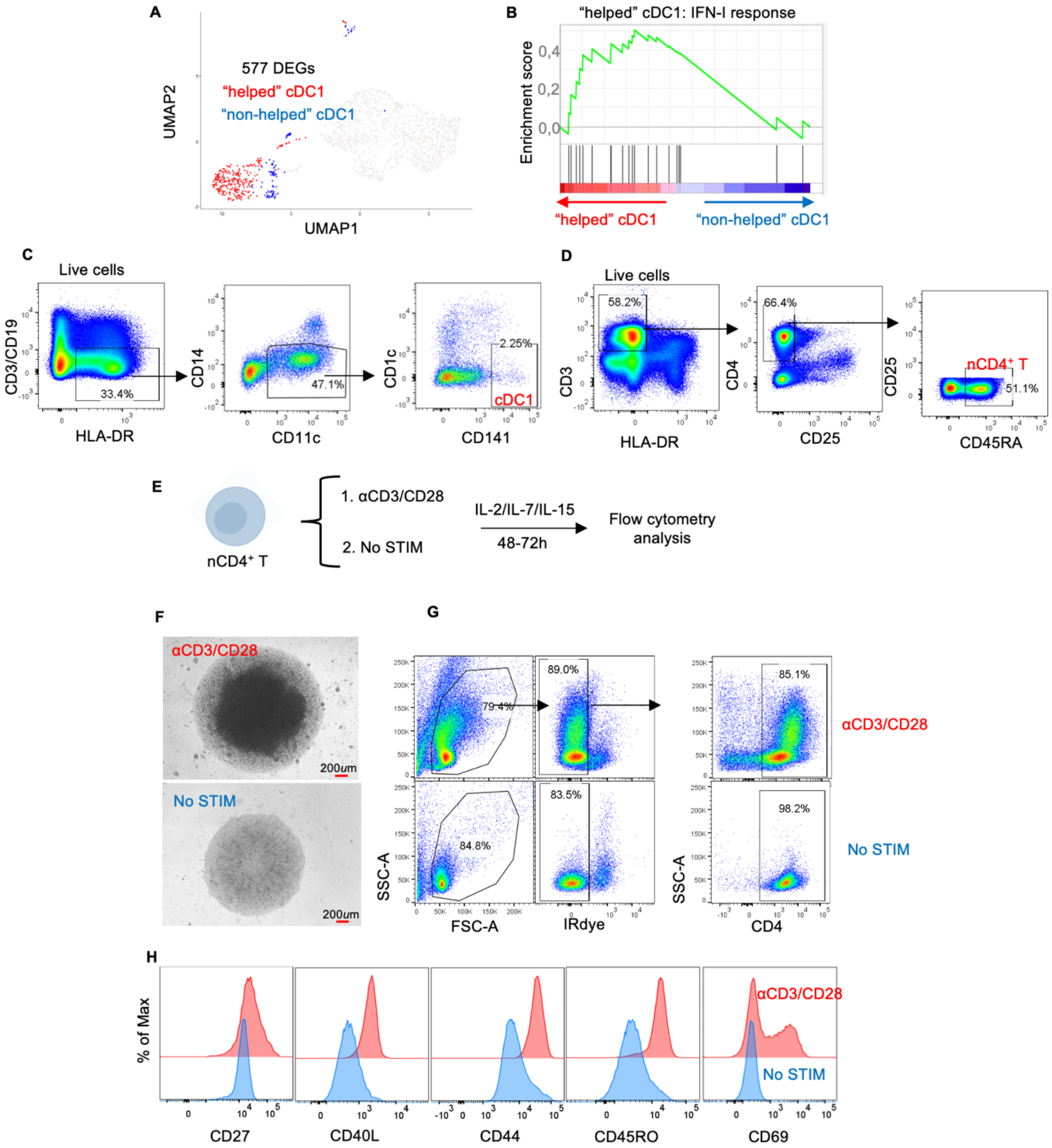

Supplementary Fig. 1. IFN-I signature is enriched in "helped" cDC1s and generation of activated CD4$^+$ T cells and cDC1s. (**A**) UMAP depicting the mRNA expression profiles of cDC1 co-cultured with activated CD4$^+$ T cells (red) and naïve CD4$^+$ T cells (blue) (6). (**B**) GSEA of the Reactome gene set "IFN-I response" in "helped" or "non-helped" cDC1s. (**C**) Gating strategies for CD11c$^+$CD141$^+$ cDC1 flow cytometrical sorting from PBMCs. (**D**) Gating strategies for CD45RA$^+$CD25$^{-/low}$ naïve CD4$^+$ T cells flow cytometrical sorting from PBMCs. (**E**) Schematic illustration of the procedure used for activating naïve CD4$^+$ T cells. (**F**) Representative light microscopic images depicting CD4$^+$ T cell culture at day 2 under indicated conditions. (**G**) Gating strategies for CD4$^+$ T cell phenotype analysis, including viability assessment. (**H**) Histogram depicting the expression of indicated T-cell activation markers on CD4$^+$ T cells under indicated conditions.

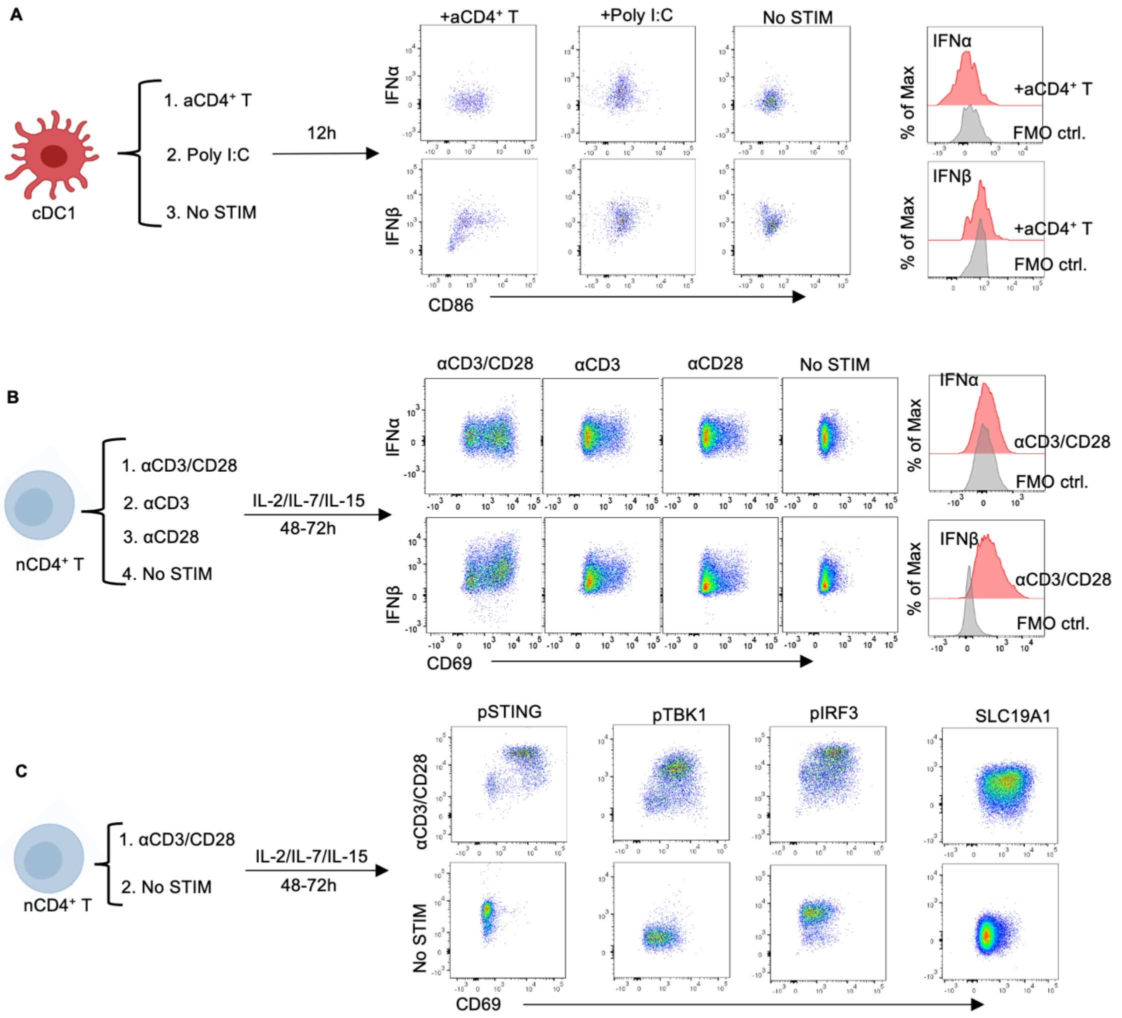

Supplementary Fig. 2. CD3/CD28-ctivated CD4⁺ T cells produce IFN-I via STING pathway activation. Purified cDC1s were stimulated with anti-CD3/CD28 activated CD4⁺ T cells or poly I:C (20 ml/ml). Purified naïve CD4⁺ T cells were stimulated with anti-CD3/CD28 or anti-CD3 only. Then cells were analyzed by flow cytometry. (**A** and **B**) Schematic illustration of the experiments, flow cytometric plots and histogram of intracellular IFNα and IFNβ expression by (**A**) cDC1s and (**B**) CD4⁺ T cells. (**C**) Schematic illustration of the experiments and flow cytometric plots depicting intracellular phospho-STING, phospho-TBK1, phospho-IRF3 and SLC19A1 expression by CD4⁺ T cells.

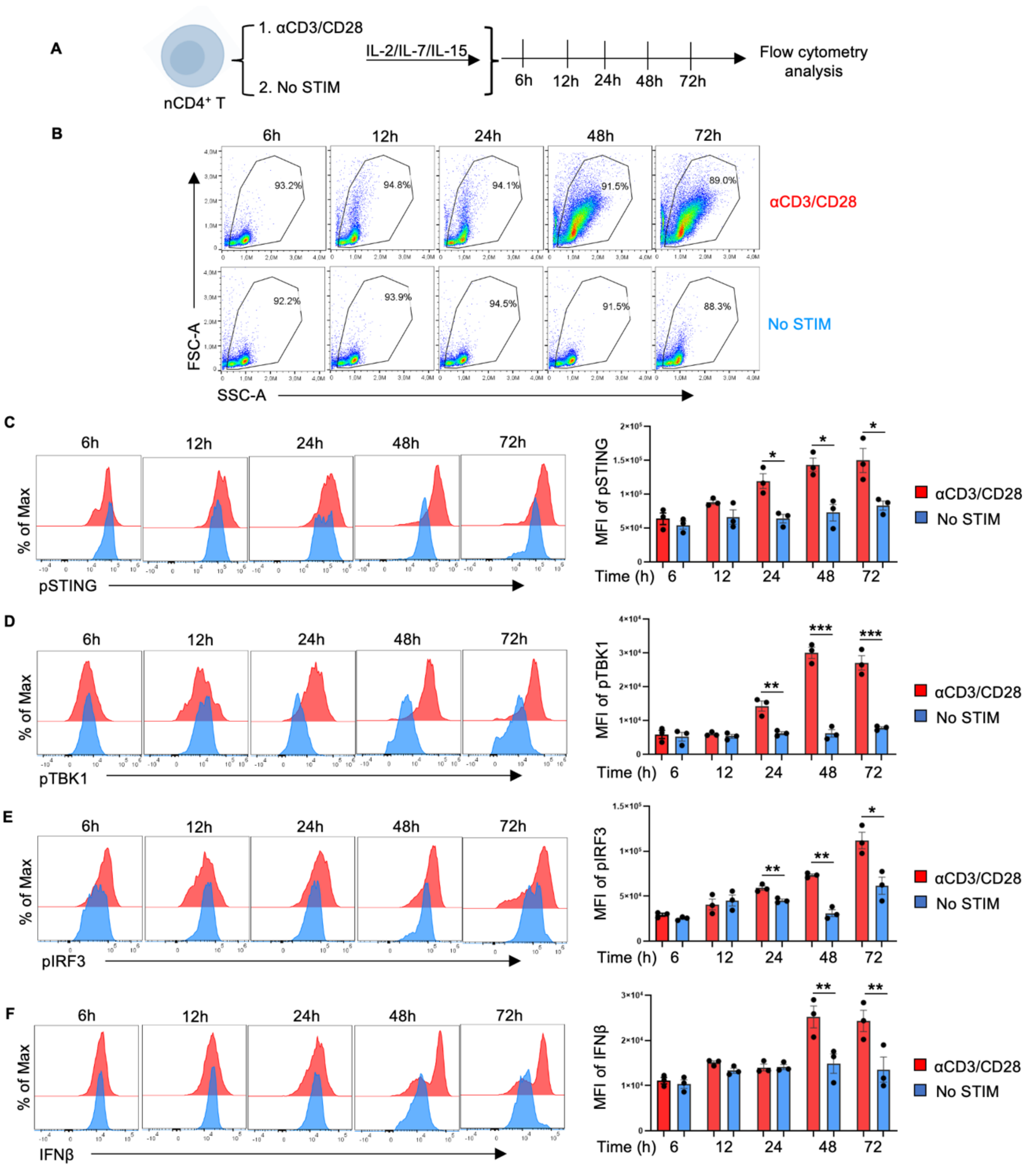

Supplementary Fig. 3. Time course of IFN-I production by CD4⁺ T cells after CD3/CD28 stimulation. Purified naïve CD4⁺ T cells were treated with or without anti-CD3/CD28 for 6h, 12h, 24h, 48h and 72h. Then cells were analyzed by flow cytometry. (**A**) Schematic illustration of the experiments. (**B**) Flow cytometric plots depicting the forward and side scatters of CD4⁺ T cells at different timepoints after CD3/CD28 stimulation. (**C-F**) Histograms (left panel) and MFI quantification bar charts (right panel) depicting phospho-STING (**C**), phospho-TBK1 (**D**), phospho-IRF3 (**E**), and intracellular IFNβ (**F**) expression by CD4⁺ T cells at indicated timepoints after anti-CD3/CD28 treatment. Data were pooled from three (n=3) independent experiments, each with technical duplicates. $P<0.05$*, $p<0.01$**, $p<0.001$***(two-tailed Mann-Whitney test). Data are shown as means ± SEMs.

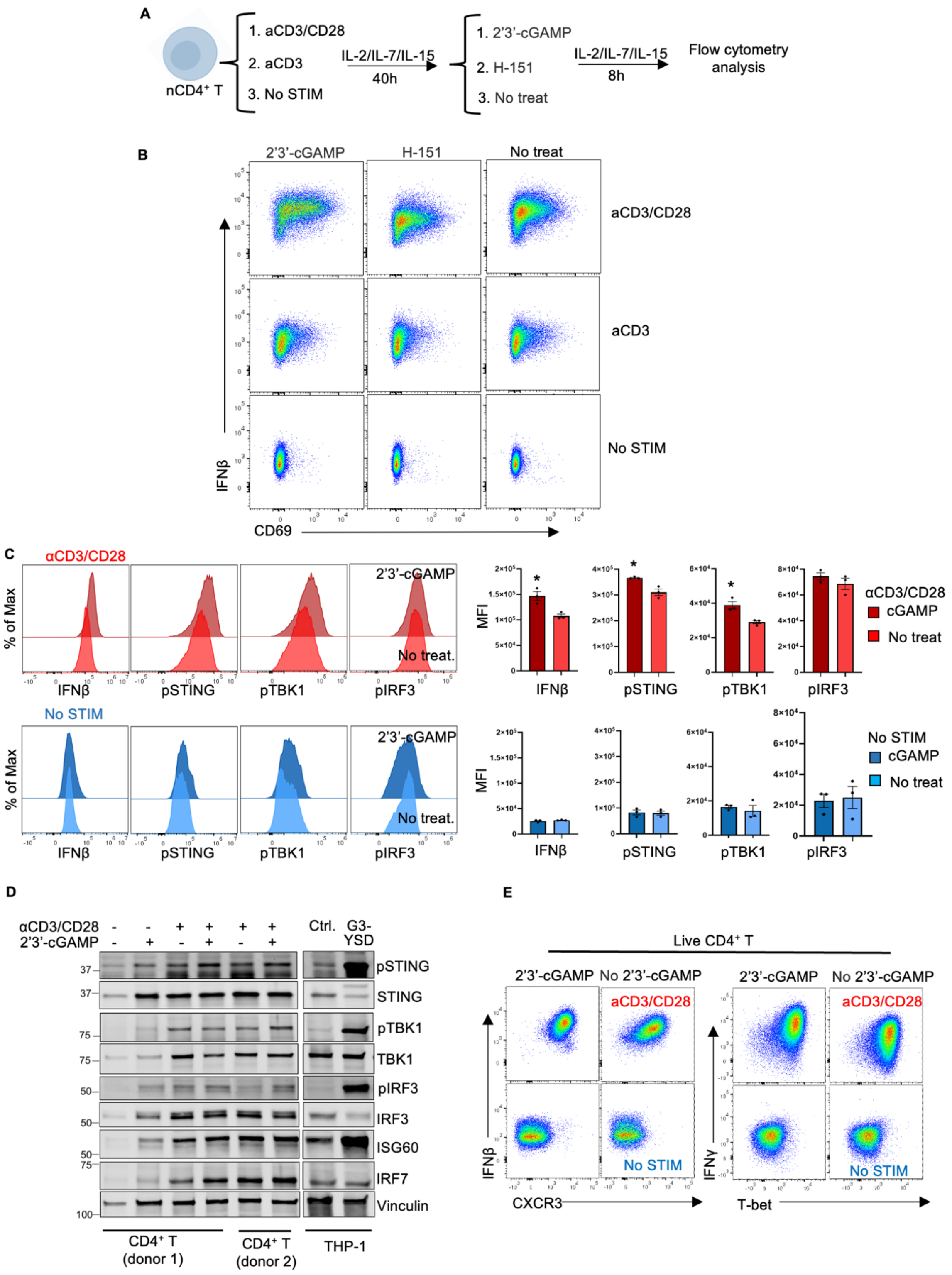

Supplementary Fig. 4. STING activation in CD4⁺ T cells induces a T-helper 1 (Th1) phenotype. Purified naïve CD4⁺ T cells were stimulated with anti-CD3/CD28 or anti-CD3 only. Additional STING inhibitor (H-151), or STING agonist (2'3'-cGAMP) was added where indicated. (**A**) Schematic illustration of the experiments. (**B**) Flow cytometry plots depicting intracellular IFNβ and cell surface CD69 expression by CD4⁺ T cells under indicated conditions. (**C**) Histograms (left panel) and MFI quantification bar charts (right panel) depicting intracellular IFNβ, phospho-STING, phospho-TBK1, and phospho-IRF3 expression by CD4⁺ T-cells under indicated conditions. (**D**) CD4⁺ T cells cultured under the indicated conditions were lysed at 48h after CD3/CD28 stimulation. STING agonist cGAMP was added in the last 8h of culture. Lysates were analyzed by SDS-PAGE and subjected to immunoblotting with the indicated antibodies. PMA-differentiated THP-1 cells that were transfected with control or cGAS agonist G3-YSD were used as control for STING pathway activation. (**E**) Flow cytometry plots depicting CXCR3, IFNγ and T-bet expression by CD4⁺ T cells under indicated conditions.

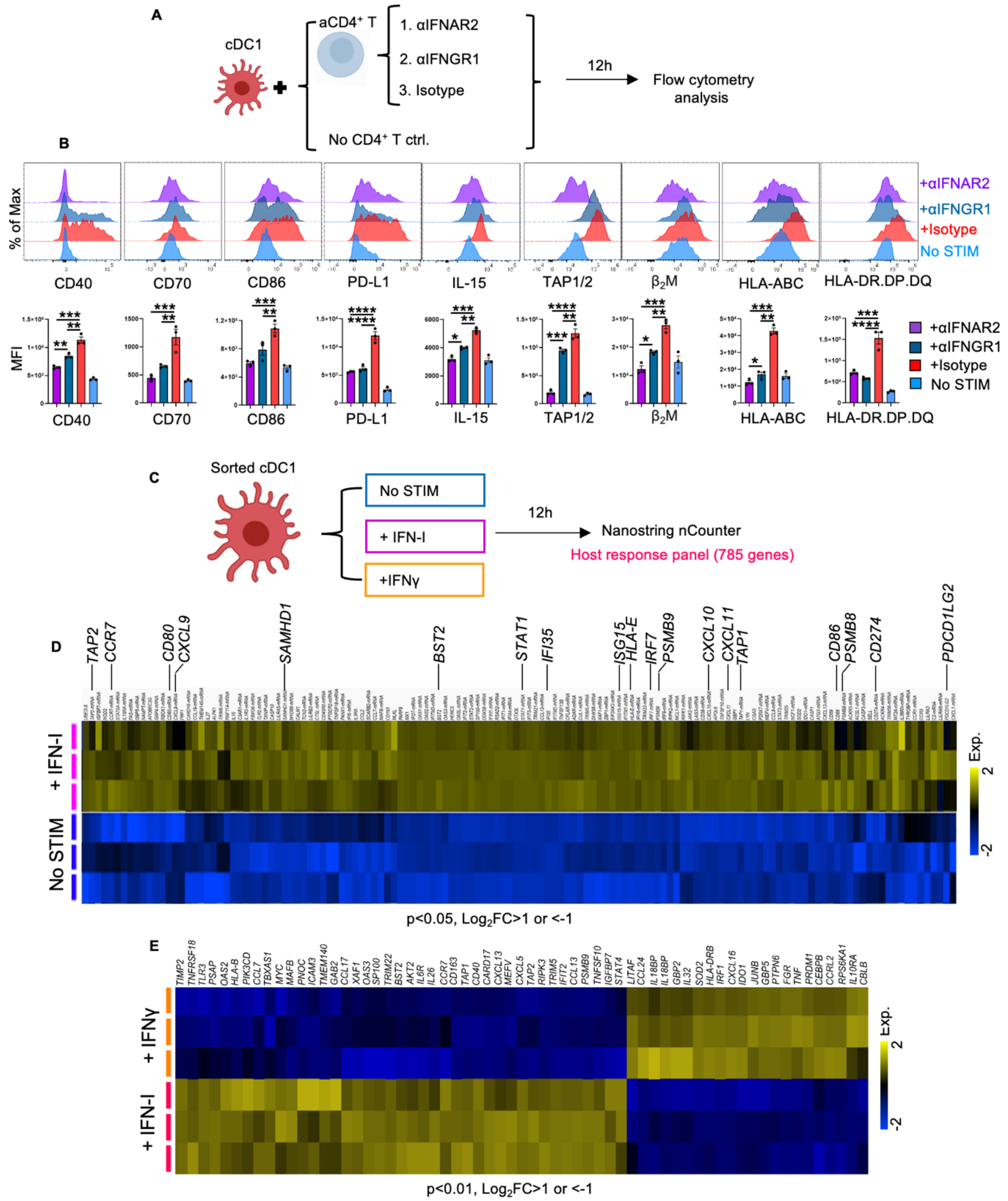

Supplementary Fig. 5. IFN-I and IFNγ signaling install different transcriptional imprints in cDC1s. (**A and B**) Purified cDC1s were cocultured with CD3/CD28-activated $CD4^+$ T cells. IFNAR2 or IFNGR1 blocking antibody (5 μg/ml) or IgG_2 isotype control were added as indicated. Key molecules of the cDC1 "help" signature (6) were analyzed by flow cytometry. (**A**) Schematic illustration of the experimental design. (**B**) Histograms (upper panel) depicting expression of indicated cDC1 "help" signature markers and bar charts (lower panel) depicting MFI quantifications of these markers under indicated conditions. (**C-E**) Purified cDC1 cultured with IFN-I (IFNα 100 U/ml + IFNβ 150 pg/ml), or IFNγ (10 ng/ml) were subjected to Nanostring nCounter analysis. cDC1s without stimulation (No STIM) were used as control. (**C**) Schematic illustration of the experimental design. (**D**) Heatmap depicting 145 DEGs between cDC1s with and without IFN-I stimulation (p-value <0.05; log_2FC >1 or <-1). (**E**) Heatmap depicting 65 DEGs between IFN-I and IFNγ stimulated cDC1s (p-value <0.01; log_2FC >1 or <-1). Data were pooled from three independent experiments in (**B**). Data are from three (n=3) independent biological samples in (**D** and **E**). P<0.05*, p<0.01**, p<0.001***(One way ANOVA test in **B**). Data are shown as means ± SEMs.

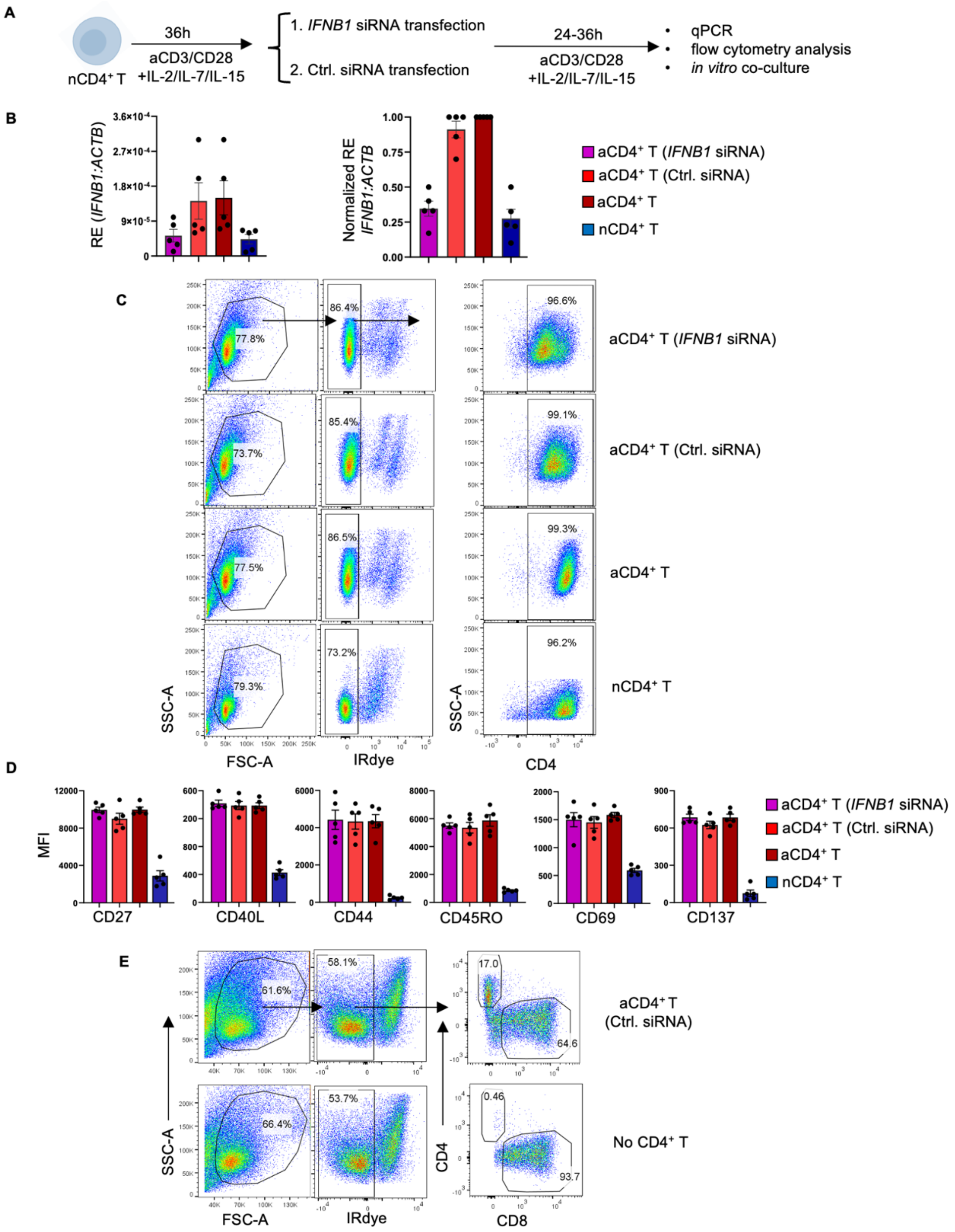

Supplementary Fig. 6. Methodology for investigating the impact of IFN-I produced by activated CD4⁺ T cells on cDC1 mediated anti-tumor CTL response. Naïve CD4⁺ T cells were activated with anti-CD3/CD28, then transfected with control siRNA or *IFNB1* siRNA. (**A**) Illustration of the experimental design for transfecting CD4⁺ T cells. (**B**) Bar charts depicting the results of quantitative (q) PCR for validating the efficiency of *IFNB1* gene knockdown. *ACTB* was used as endogenous control. (**C**) Gating strategies for analyzing *IFNB1* gene knockdown efficiency. (**D**) MFI quantifications for the expression of indicated T cell activation markers under indicated conditions. (**E**) Gating strategies for flow cytometric analysis of CD8⁺ T cell response from the tumor antigen-specific CTL priming system (6). Data were pooled from five (n=5) independent experiments in (**B** and **D**). Data are shown as means ± SEMs.

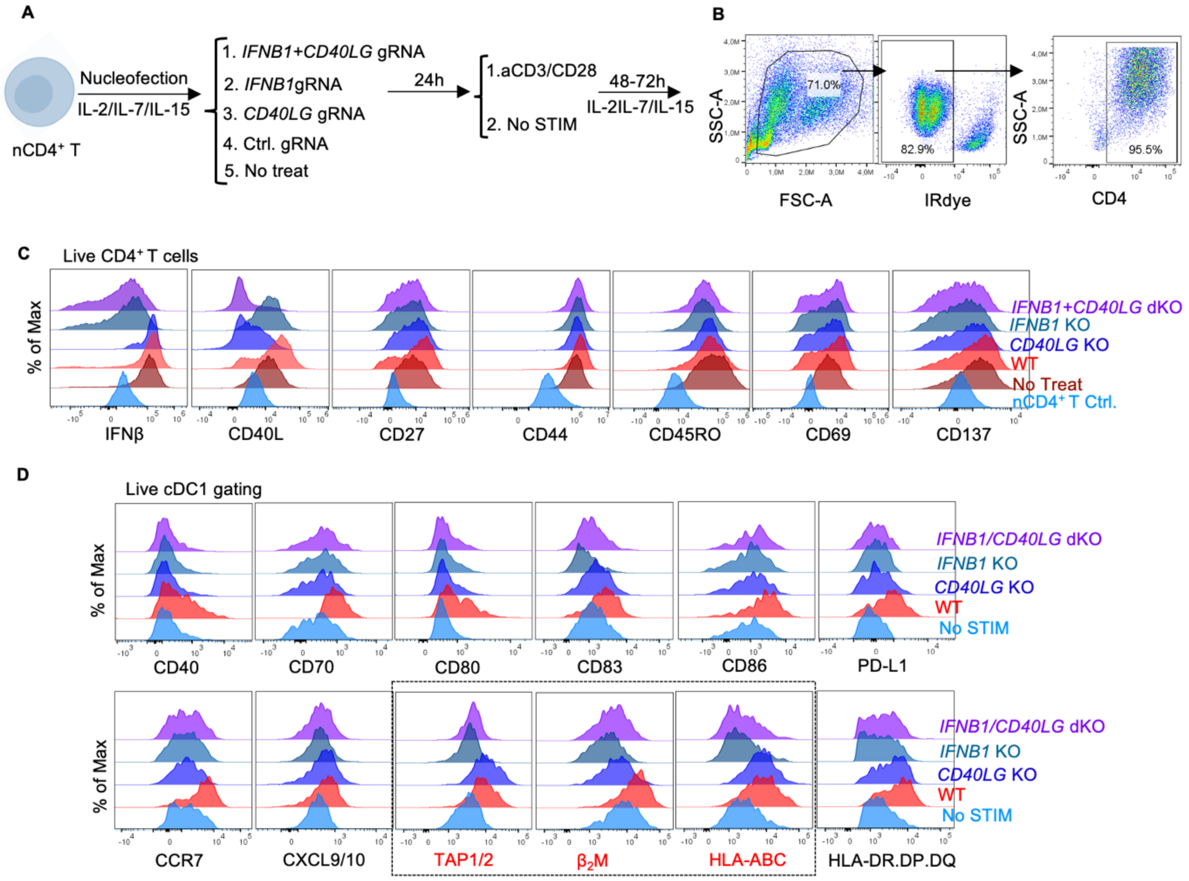

Supplementary Fig. 7. Investigating the role of CD4⁺ T cell help via IFN-I and CD40 signaling in cDC1s for optimal T cell priming. (**A**) Illustration of CRISPR-Cas9 ribonucleoprotein (RNP) procedure for CD4⁺ T cells. (**B**) Gating strategy for analyzing CD4⁺ T cell phenotype. (**C**) Histograms depict the intracellular IFNβ expression, surface expression of CD40L and other indicated markers identifying effector-(memory) T cells under indicated conditions. (**D**) Purified cDC1s were stimulated with indicated CD4⁺ T cells. Histograms depict the expression of indicated molecules by cDC1s. Black box highlights the molecules involved in MHC-I antigen presentation.

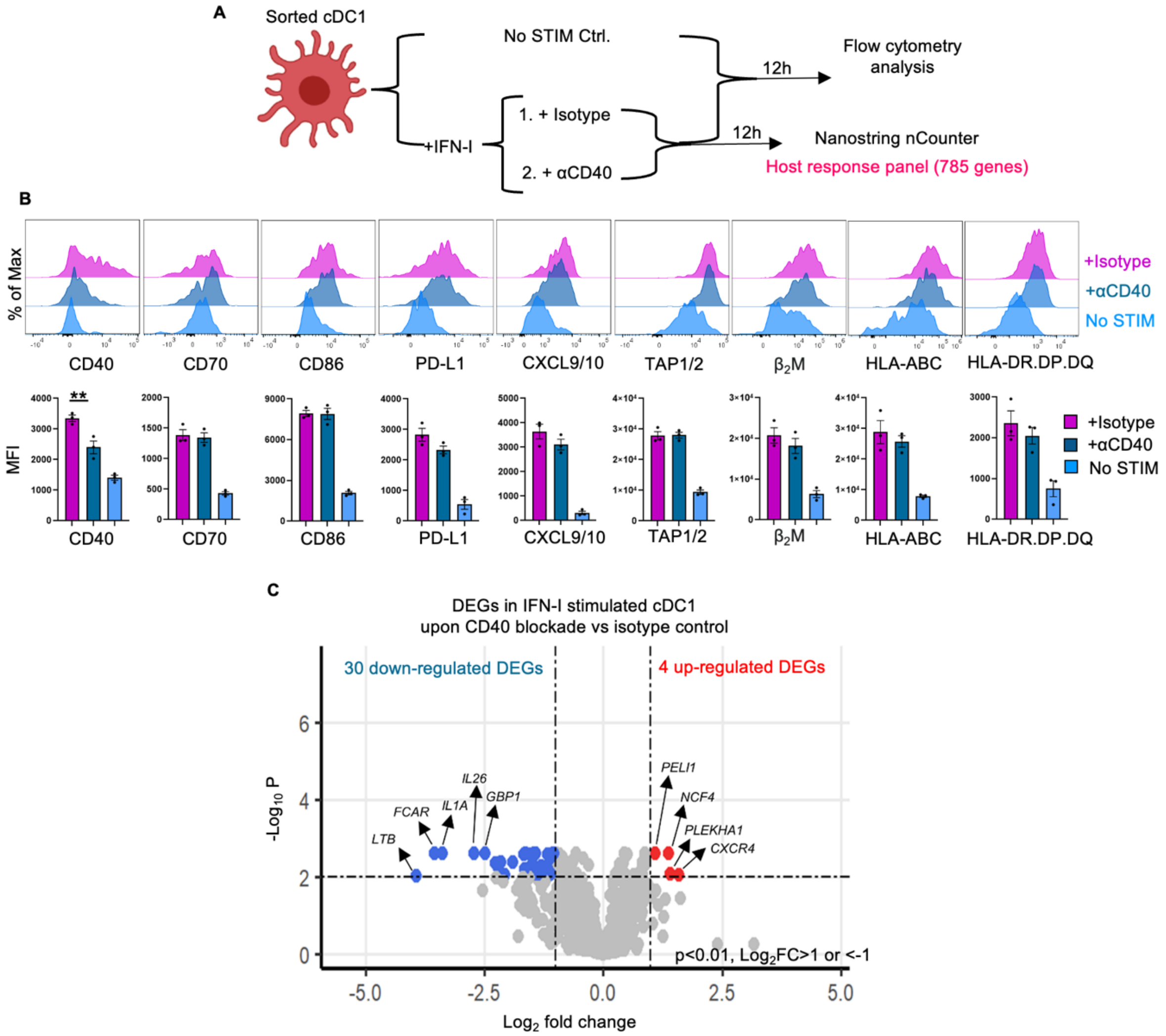

Supplementary Fig. 8. Human cDC1-licensing via IFN-I is CD40 signaling independent. Purified cDC1s were stimulated with IFN-I (IFNα 100 U/ml + IFNβ 150 pg/ml), then CD40 blocking antibody or isotype control was added as indicated. Key molecules of cDC1 "help" signature (6) were analyzed by flow cytometry. Additionally, cells were lysed and subjected to Nanostring nCounter analysis. (**A**) Schematic illustration of the experimental design. (**B**) Histograms (upper panel) depicting expression of indicated cDC1 "help" signature markers and bar charts (lower panel) depicting MFI quantifications of these markers measured by flow cytometry under indicated conditions. (**C**) Volcano plot depicting DEGs between CD40 blockade versus isotype control conditions in IFN-I stimulated cDC1s. Genes indicated in red are significant with p-values<0.01 and $log_2FC>1$, genes indicated in blue are significant with p-values<0.01 and $log_2FC<-1$ in CD40 blockade condition. Data are pooled from three (n=3) independent experiments in (**B**). Data are from three (n=3) independent biological samples in (**C**). p<0.01** (One-way ANOVA in **B**). Data are shown as means ± SEMs.

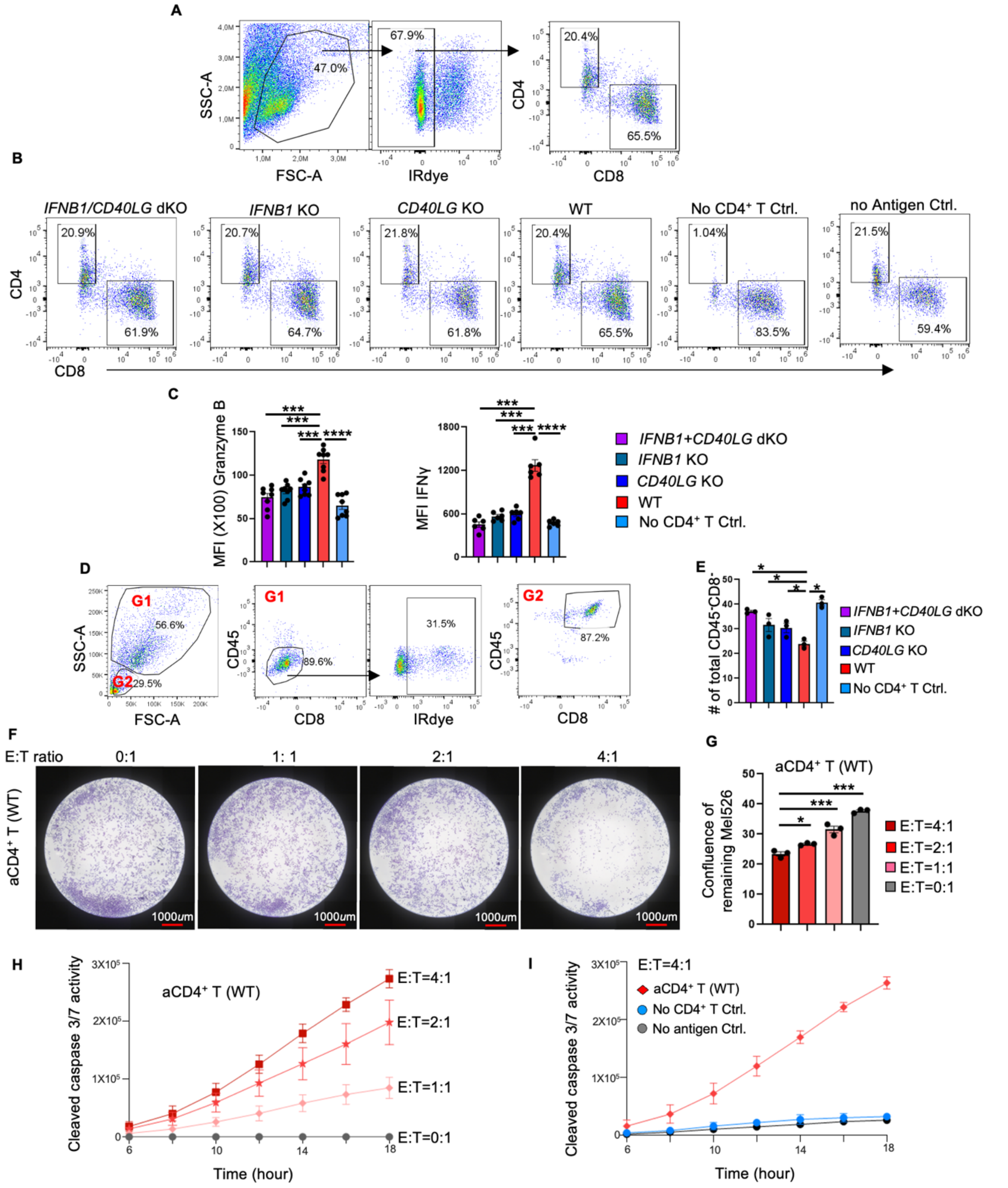

Supplementary Fig. 9. Investigating the role of CD4⁺ T cell help via IFN-I and CD40 signaling in cDC1s for CTL killing capacity. (**A-C**) Our tumor antigen-specific CTL priming system (6) was used to investigate the impact of IFN-I and CD40L produced by CD4⁺ T cells on cDC1-mediated CTL response. (**A**) Gating strategies for detecting CD8⁺ T cell responses. (**B**) Plots depict the gating of CD8⁺ Tce lls from co-cultures under indicated conditions. (**C**) MFI quantifications of Granzyme B and IFNγ expressed by CTV$^{(-)}$CD8⁺ T cells. (**D-I**) Live MART-1-specific CD8⁺ T cells were purified 5 days after priming, plated onto live Mel526 cells at different E:T ratios and analyzed by IncuCyte during 18h At the end of the assay, cell suspension from each condition (regardless of E:T ratio) was pooled and analyzed by flow cytometry. Surviving Mel526 cells in each well were fixed and stained with crystal violet. (**D**) Gating strategies of cells in suspension collected at the end of killing assay. (**E**) Number (#) of total Mel526 cells in suspension under indicated conditions. (**F**) Representative CCD microscopic images depicting survived Mel526 cells remained in the plate at the end of killing assay under help condition with different E:T ratios. (**G**) Quantified confluence of surviving Mel526 cells in the plate at the end of killing assay under help condition with different E:T ratios. (**H**) Cleaved caspase 3/7 activity in tumor cells confronted with "helped" CTL at different E:T ratios. (**I**) Caspase 3/7 activity in tumor cells confronted with "non-helped" CTL at E:T=4:1 ratio. P<0.05*, p<0.01**, p<0.001*** p<0.0001**** (One-way ANOVA in **C, E** and **G**). Data were pooled from eight (n=8 in **C**), and three (n=3 in **E** and **G**) independent experiments, each with technical duplicates. Data are shown as means ± SEMs.

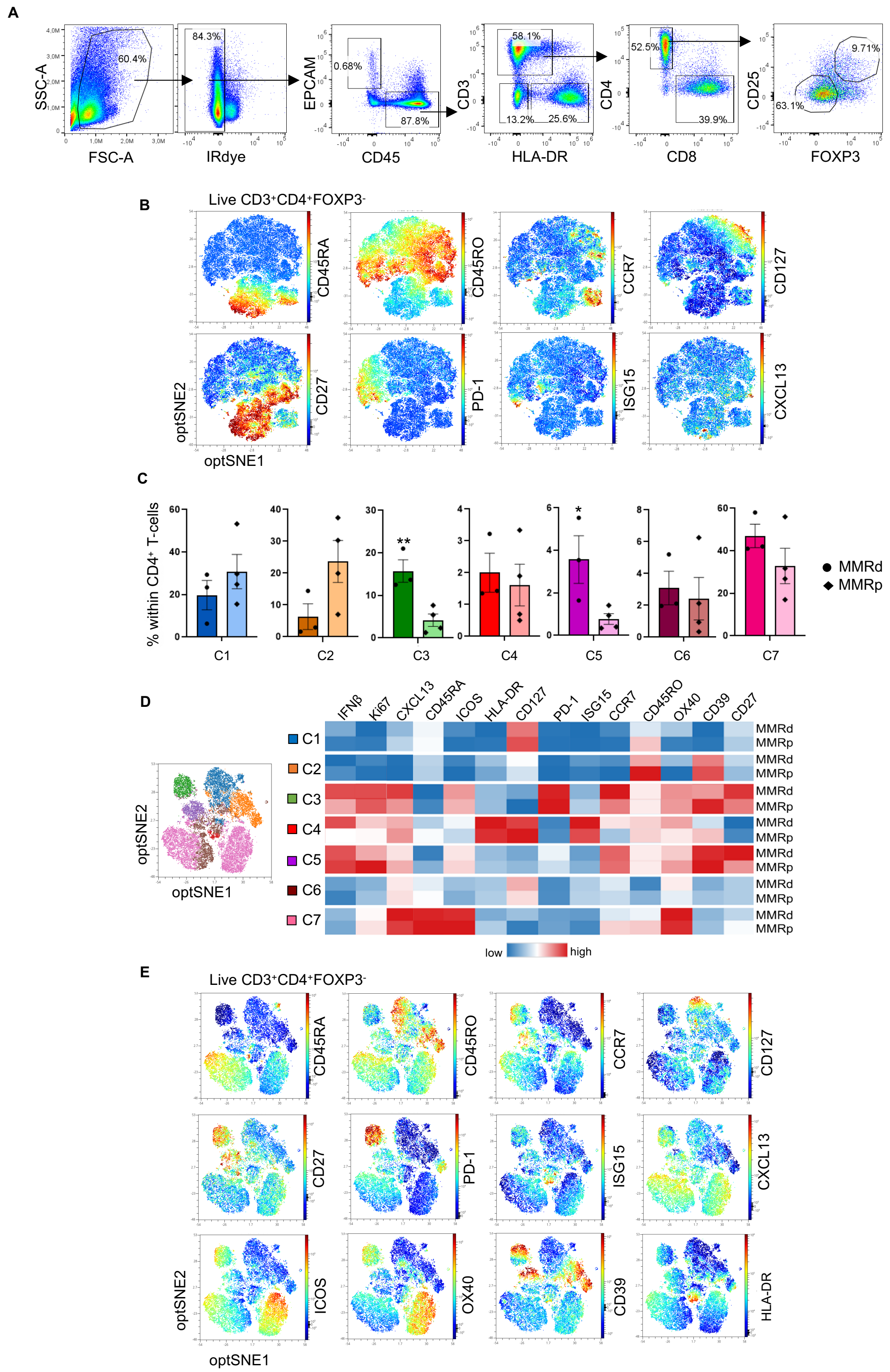

Supplementary Fig. 10. IFNβ is detected in tumor-infiltrating Ki67$^+$CXCL13$^+$CD4$^+$ T cells at the protein level. Tumor-infiltrating FOXP3$^-$CD4$^+$ T cells from HNSC (n=7) and CRC (n=7) patients were analyzed by flow cytometry. (**A**) Gating strategy for tumor-infiltrating FOXP3$^-$CD4$^+$ T cells. (**B**) OptSNE plots depicting indicated markers expressed by CD4$^+$ T cells from HNSC patients. (**C**) Bar charts depicting proportion of each cluster identified in CD4$^+$ T cells from CRC patients. (**D**) Heatmap depicting median expression values of indicated molecules in seven CD4$^+$ T cell clusters indicated in optSNE plot (left) from MMRd and MMRp CRC patients. (**E**) OptSNE plots of indicated molecules expressed by CD4$^+$ T cells derived from CRC patients.

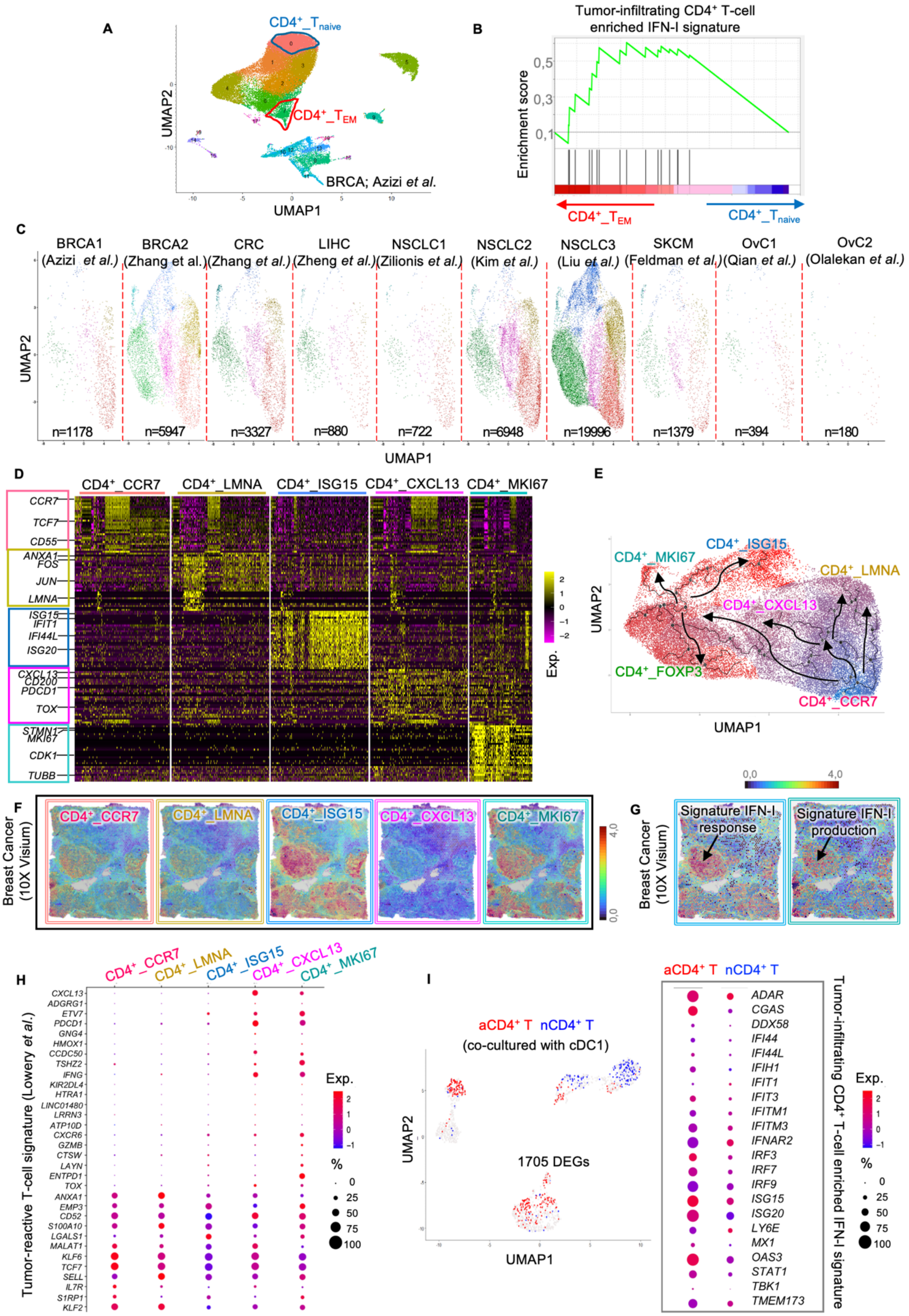

Supplementary Fig. 11. Tumor-infiltrating $CD4^+$ T cells enriched in IFN-I signaling are conserved across multiple tumor types. (**A**) UMAP depicting scRNAseq of immune cells (colored by clusters) from resection specimens of eight primary breast tumors (37). After clustering, effector-(memory) $CD4^+$ T cells ($CD4^+$ $T_{\_EM}$) and naïve $CD4^+$ T-cells ($CD4^+$ $T_{\_naive}$) were selected for further analysis. (**B**) GSEA of IFN-I signature from Reactome gene set in tumor-infiltrating $CD4^+$ $T_{\_E(M)}$ vs. $CD4^+$ $T_{\_naive}$. (**C-E**) 10 scRNA-Seq studies were collected and

integrated, 5 FOXP3$^-$CD4$^+$ T cell clusters were identified. (**C**) UMAP of tumor-infiltrating CD4$^+$ T cell clusters identified based on scRNAseq dataset from indicated studies. (**D**) Heatmap depicting the scaled expression values of top 30 up-regulated DEGs in tumor-infiltrating CD4$^+$ T cell clusters. (**E**) Pseudo-temporal ordering of cells colored from blue to red according to maturation state. Lines represent differentiation trajectories of tumor-infiltrating CD4$^+$ T cells. (**F** and **G**) Distribution of tumor-infiltrating CD4$^+$ T cell signatures and IFN-I signature from tumor-infiltrating CD4$^+$ T cells in breast tumor section profiled by spatial transcriptomics (10x Visium). (**H**) scRNAseq plot depicting the scaled expression of tumor-(neo)antigen specific T cell signature (39) in tumor-infiltrating CD4$^+$ T-cell clusters we identified. (**I**) Activated- or naive CD4$^+$ T cells co-cultured with cDC1s (6) were subjected to 10X Genomics scRNAseq. UMAP depicting the mRNA expression profiles of activated CD4$^+$ T cells (red) and naïve CD4$^+$ T cells (blue) (left); Dot plot depicting the scaled expression values of IFN-I signature from tumor-infiltrating CD4$^+$ T cells in activated- or naive CD4$^+$ T cells (right).

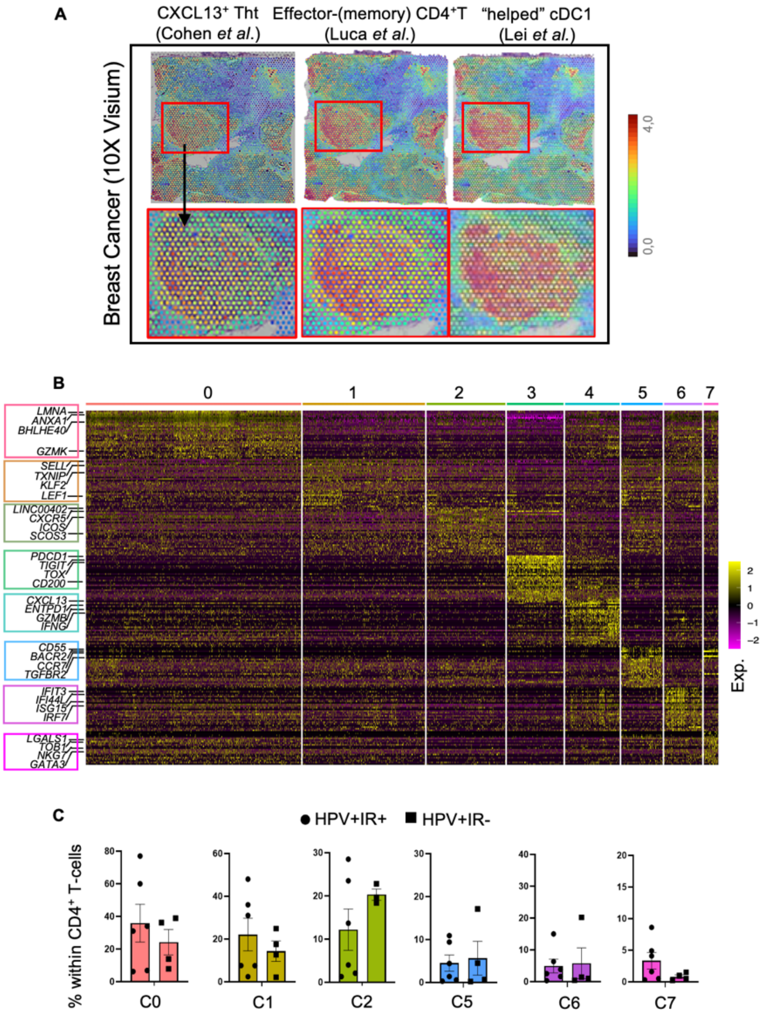

Supplementary Fig. 12. Tumor-infiltrating effector-(memory) CD4$^+$ T cells that are enriched in IFN-I signature co-localize with "helped" cDC1s. (**A**) Distribution of CXCL13$^+$ Tht signature (40), tumor-infiltrating CD4$^+$ T cell signature in CE9 (19) and "helped" cDC1 signature (6) in breast tumor section profiled by spatial transcriptomics (10x Visium). (**B**) Heatmap depicting the scaled expression values of top 30 up-regulated DEGs in tumor-infiltrating FOXP3$^-$CD4$^+$ T cell clusters of HPV$^+$ OPSCC patients (43). (**C**) Distribution of tumor-infiltrating conventional CD4$^+$ T cells from IR$^+$ and IR$^-$ groups of HPV$^+$ OPSCC patients among clusters 0-2 and clusters 5-7.

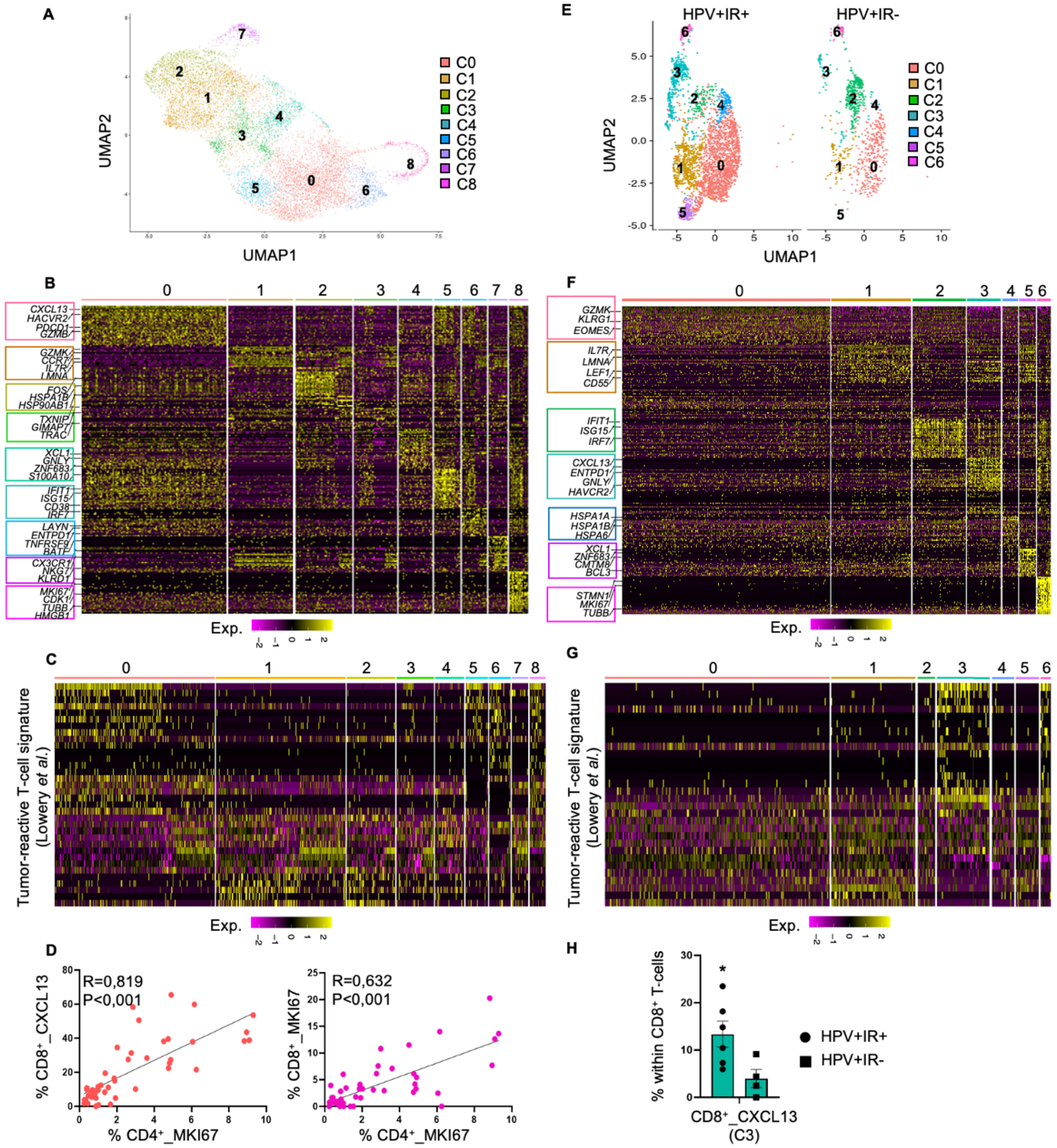

Supplementary Fig. 13. Tumor-infiltrating Ki67$^+$(CXCL13$^+$) CD4$^+$ T cells are positively associated with tumor-reactive CD8$^+$ T cells. (**A-D**) The 10 scRNAseq datasets related to Figure 7 were collected and integrated and 9 CD8$^+$ T cell clusters were identified. (**A**) UMAP of tumor-infiltrating CD8$^+$ T cell clusters identified. (**B**) Heatmap depicting the scaled expression values of top 30 up-regulated DEGs in tumor-infiltrating CD8$^+$ T cell clusters. (**C**) Heatmap depicting the scaled expression of the tumor (neo)antigen specific-T cell signature (39) in tumor-infiltrating CD8$^+$ T cell clusters. (**D**) Spearman correlation between the frequencies of the CD4$^+$_MKI67 population and the frequencies of the CD8$^+$_CXCL13 (cluster 0) or the CD8$^+$_MKI67 (cluster 8) population. R, correlation coefficient. (**E-H**) scRNAseq of tumor-infiltrating CD8$^+$ T cells from HPV$^+$ OPSCC patients (43) was analyzed. (**E**) UMAP of seven clusters identified in IR$^+$ and IR$^-$ groups respectively. (**F**) Heatmap depicting the scaled expression values of top 30 up-regulated DEGs in tumor-infiltrating CD8$^+$ T cell clusters. (**G**) Heatmap depicting the scaled expression of tumor (neo)antigen specific T cell signature (39) in tumor-infiltrating CD8$^+$ T cell clusters. (**H**) Frequency of CD8$^+$_CXCL13 (cluster 3) cells among the tumor-infiltrating CD8$^+$ T cells from the IR$^+$ and IR$^-$ groups. Correlation analysis as shown in D for the OPSCC cohort was not performed due to an insufficient sample size.

Supplemental table 1. (Excel file)
Genes of IFN-I signaling and IFNγ signaling enriched in "helped" cDC1s.

Supplemental table 2. (Excel file)
Supplementary table 2.1. lists the normalized log2 count data of genes detected in IFN-I, IFN-I+CD40 blockade, or IFNγ stimulated cDC1s (the columns '+IFN-I', "IFN-I+αCD40", "IFNγ") and non-treated cDC1s (the column 'No STIM) from 3 independent biological donors using NanoString nCounter. Supplementary table 2.2. lists DEGs in IFN-I stimulated cDC1s comparing to non-treated cDC1s. Supplementary table 2.3. lists DEGs in IFN-I stimulated cDC1s comparing to IFNγ stimulated cDC1s. Supplementary table 2.4. lists DEGs in IFN-I stimulated cDC1s comparing to IFN-I+CD40 blockade stimulated cDC1s. Supplementary data 2.5. lists genes of IFN-I signaling and pathways of adaptive immune responses. P-values and adjusted p-values were calculated using unpaired two-tailed student t test.

Supplemental table 3. (Excel file)
Genes of IFN-I signaling enriched in tumor-infiltrating effector-(memory) $CD4^+$ T cells.

Supplemental table 4. (Excel file)
Supplementary table 6. lists top 100 DEGs in tumor-infiltrating $CD4^+$ T cell clusters derived from 10 single cell studies indicated in Supplementary Figure 11. P-values and adjusted p-values were calculated using non-parametric Wilcoxon rank sum test. The column 'avg_log2FC' represents the average log (normalized expression of the current group of cells/normalized expression of the rest of the cells). The column 'pct.1' represents the percentage of cells expressing a certain gene in the current group of cells. The column pct.2 represents the percentage of cells expressing the same gene in the rest of the cells.

Supplemental table 5. (Excel file)
Supplementary table 5.1. lists genes of $CXCL13^+$ T helper tumor-specific cell (Tht) signature from Cohen *et al*.. Supplementary table 5.2. lists genes of tumor-infiltrating $CD4^+$ T cell signature in carcinoma ecotype 9 (CE9) from Luca *et al*.. Supplementary table 5.3. lists genes of tumor-reactive T cell signature in from Lowery *et al*..

Supplementary table 6. (Excel file)
Supplementary table 6. lists top 100 DEGs in tumor-infiltrating $CD8^+$ T cell clusters derived from 10 single cell studies indicated in Supplementary Figure 11. P-values and adjusted p-values were calculated using non-parametric Wilcoxon rank sum test. The column 'avg_log2FC' represents the average log (normalized expression of the current group of cells/normalized expression of the rest of the cells). The column 'pct.1' represents the percentage of cells expressing a certain gene in the current group of cells. The column pct.2 represents the percentage of cells expressing the same gene in the rest of the cells.

Supplemental table 7. (Excel file)

Supplementary table 7. lists top 100 DEGs in tumor-infiltrating $FOXP3^-CD4^+$ T cell and $CD8^+$ T cell clusters derived from HPV^+ OPSCC patients. P-values and adjusted p-values were calculated using non-parametric Wilcoxon rank sum test. The column 'avg_log2FC' represents the average log (normalized expression of the current group of cells/normalized expression of the rest of the cells). The column 'pct.1' represents the percentage of cells expressing a certain gene in the current group of cells. The column pct.2 represents the percentage of cells expressing the same gene in the rest of the cells.

Supplemental table 8. (Excel file)

Supplementary table 8.1. provides an overview of clinical characteristics of head and neck squamous carcinoma patients. Supplementary table 8.2. provides an overview of clinical characteristics of colorectal carcinoma patients.

Supplemental table 9. (Excel file)

Overview of antibodies, related reagents, publicly available datasets, software and algorithms used in the current study.
