# Supplementary material for: CD4+ T cells produce IFN-I to license cDC1s for induction of cytotoxic T-cell activity in human tumors
Source: Cell Mol Immunol. 2024 Feb 21;21(4):374–92. doi: 10.1038/s41423-024-01133-1 (PMC10978876; doi:10.1038/s41423-024-01133-1)
Supplement: Supplementary file 11 — source file for immunoblotting related to supplementary Figure 4D [file 41423_2024_1133_MOESM11_ESM.pdf]

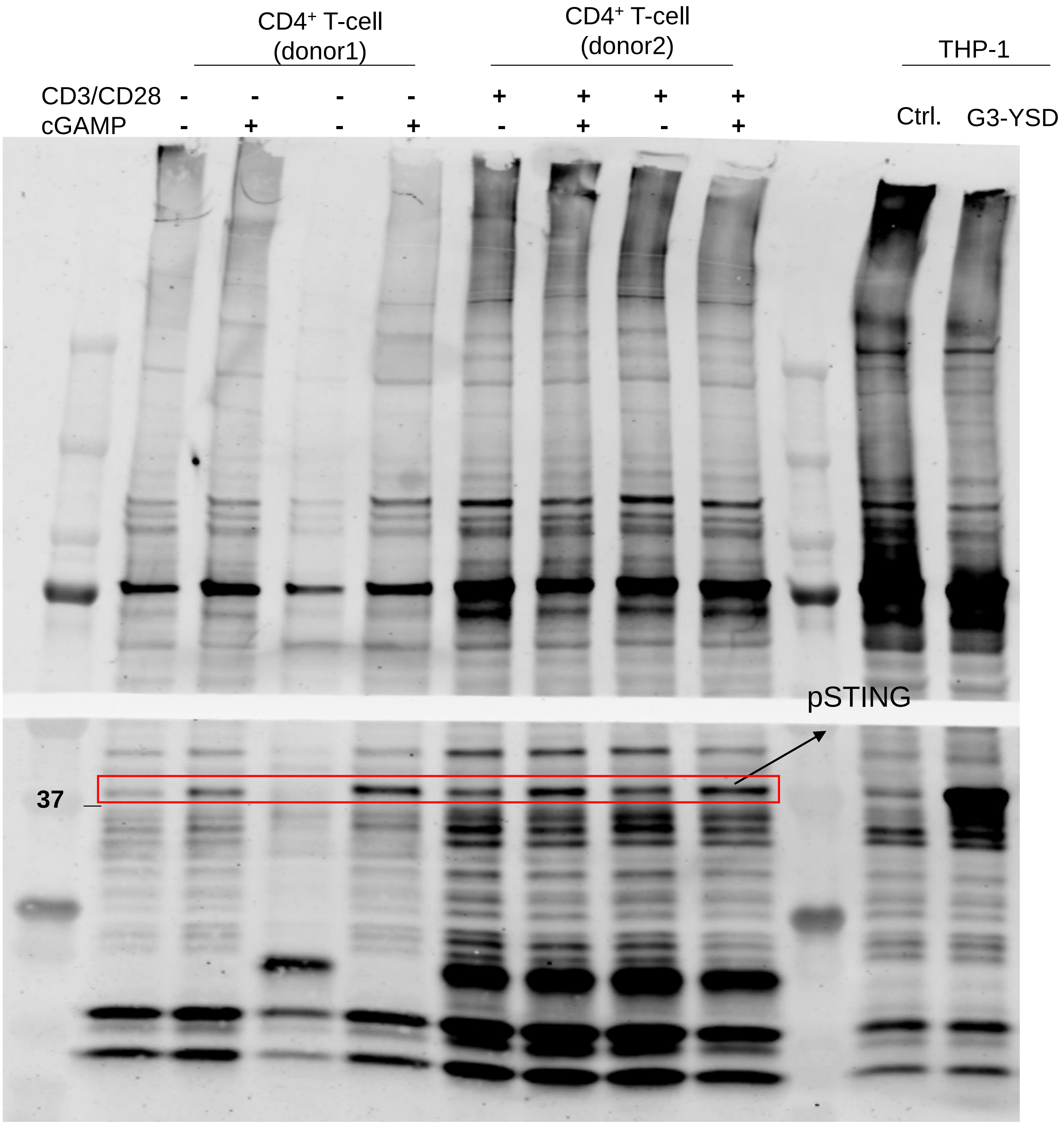
CD4+ T-cell (donor1)
CD4+ T-cell (donor2)
THP-1
CD3/CD28 - - - - + + + +
cGAMP - + - + - + - +
Ctrl.
G3-YSD
pSTING
37

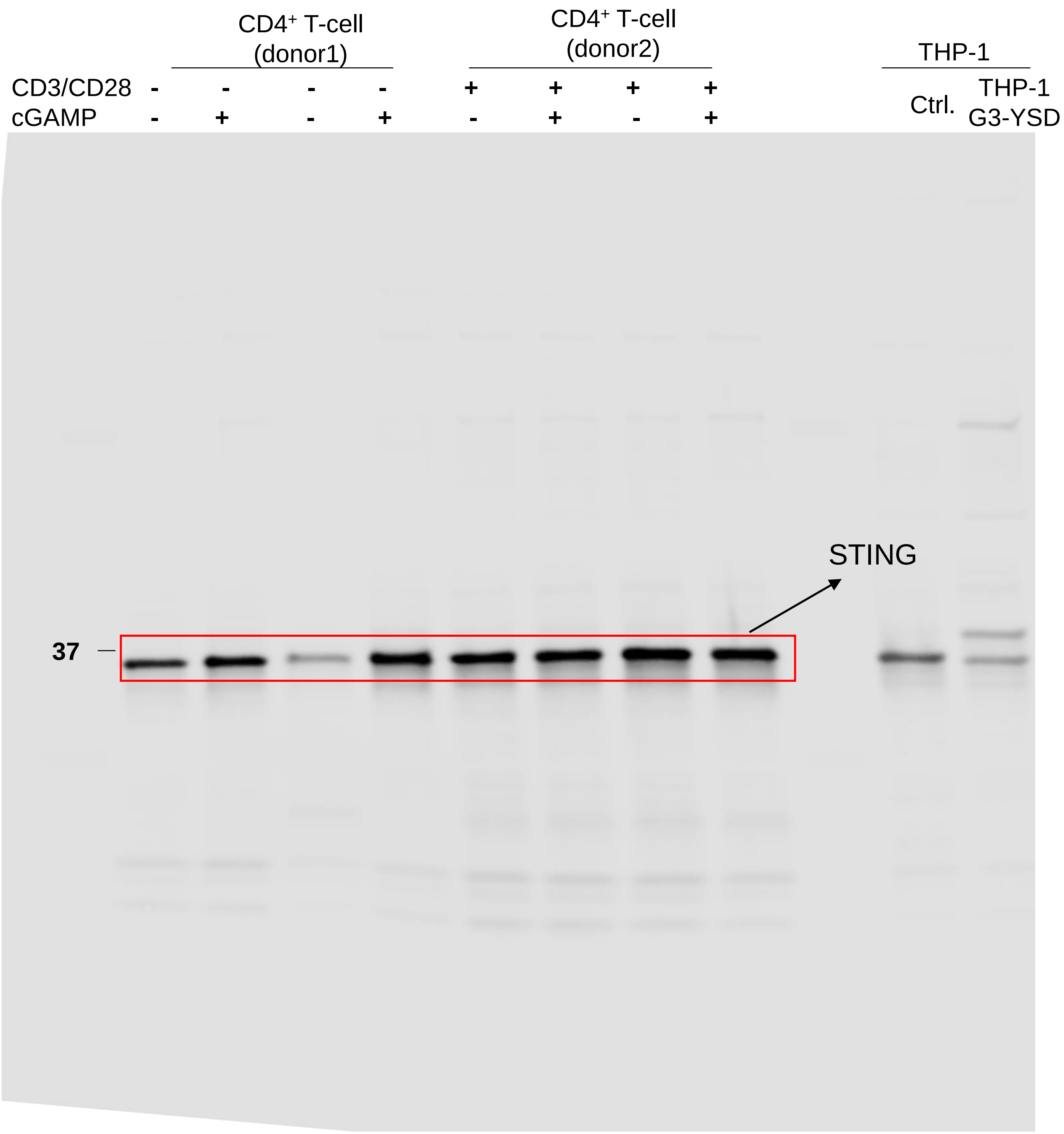
CD4+ T-cell (donor1)
CD4+ T-cell (donor2)
THP-1
CD3/CD28 - - - - + + + +
cGAMP - + - + - + - +
Ctrl.
THP-1 G3-YSD
STING
37

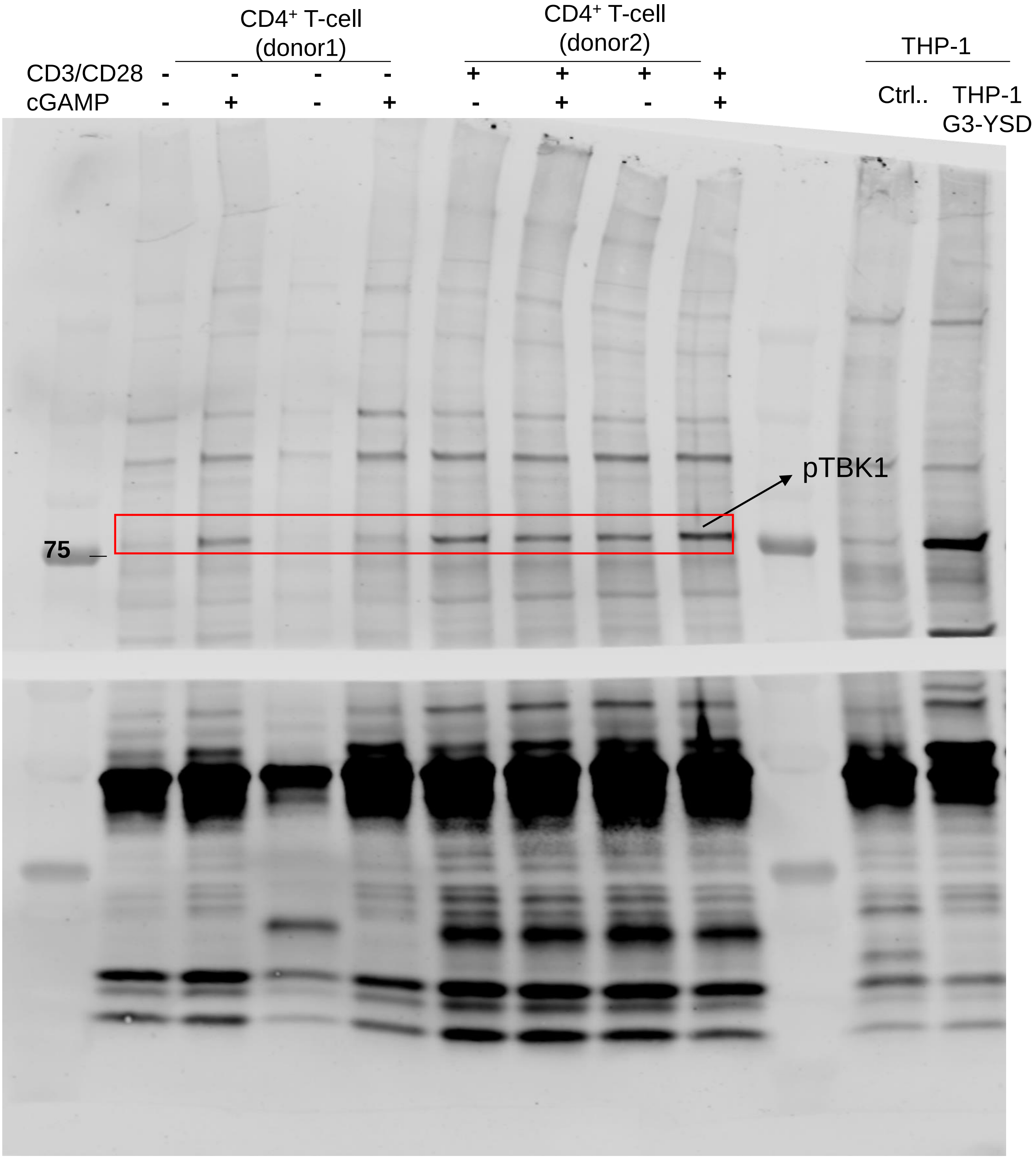
CD4+ T-cell (donor1)
CD4+ T-cell (donor2)
THP-1
CD3/CD28 - - - - + + + +
cGAMP - + - + - + - +
Ctrl..
THP-1 G3-YSD
pTBK1
75

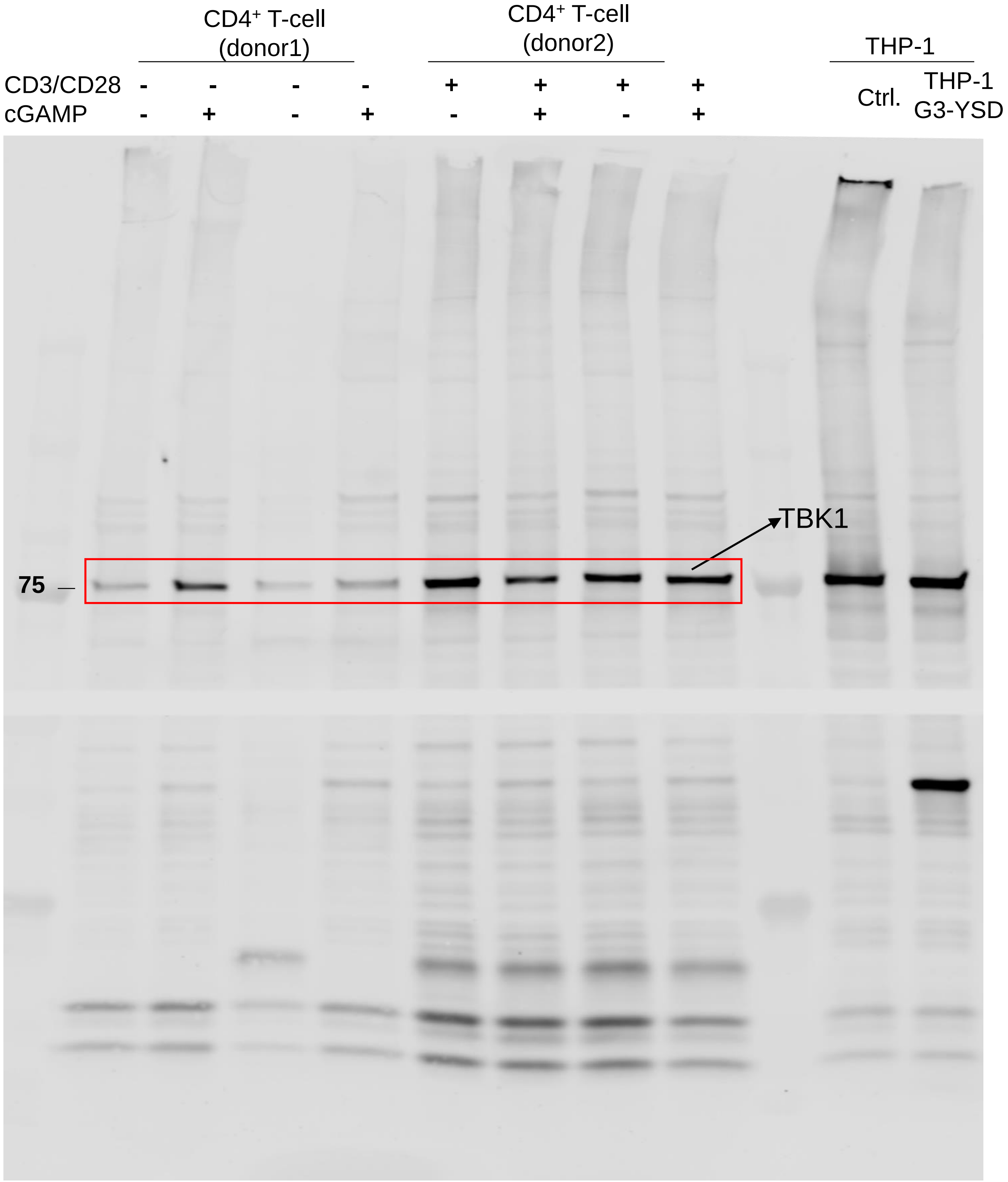

CD4+ T-cell (donor1)
CD4+ T-cell (donor2)
THP-1
CD3/CD28 - - - - + + + +
cGAMP - + - + - + - +
Ctrl.
THP-1 G3-YSD
TBK1
75

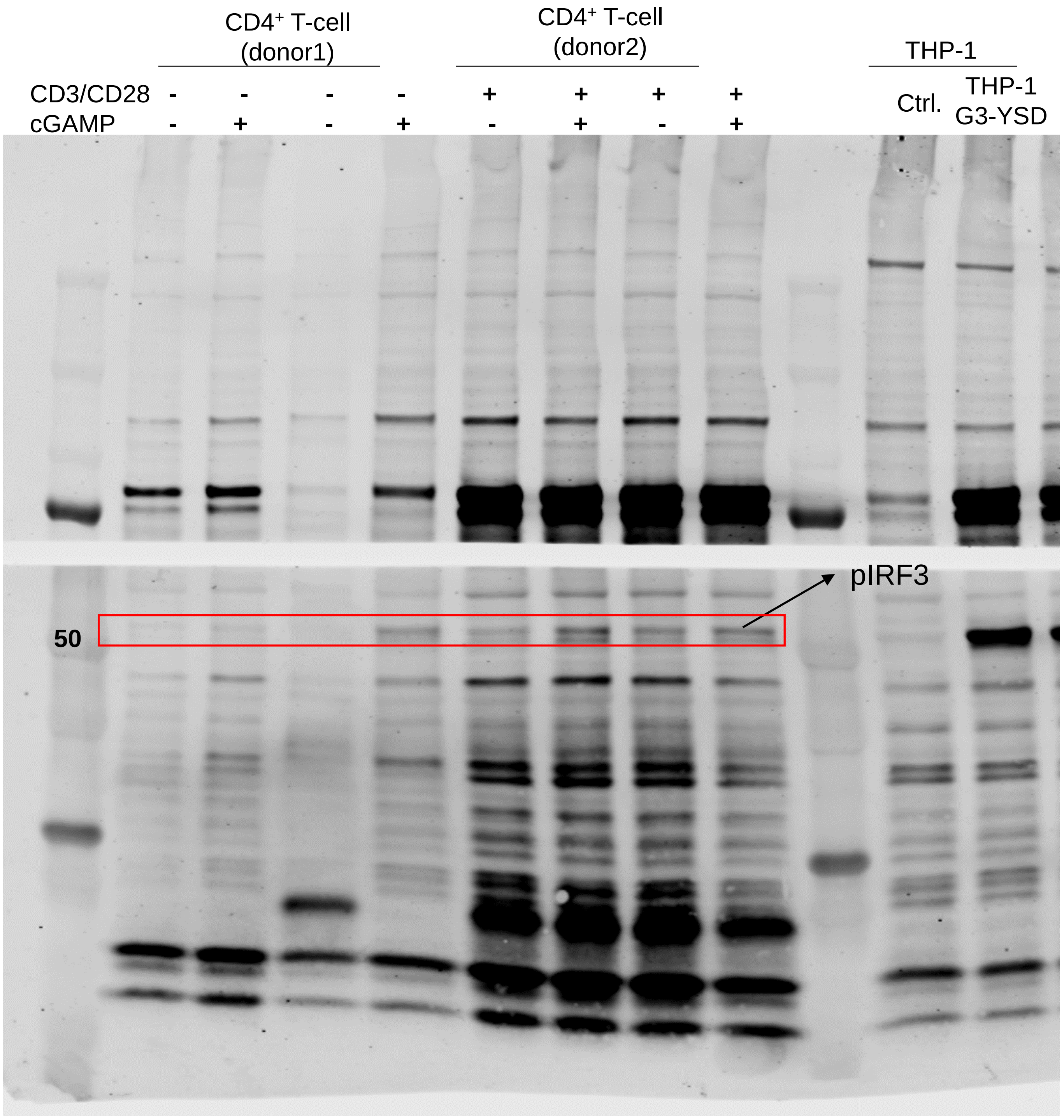
CD4+ T-cell (donor1)
CD4+ T-cell (donor2)
THP-1
CD3/CD28 - - - - + + + +
cGAMP - + - + - + - +
Ctrl.
THP-1 G3-YSD
pIRF3
50

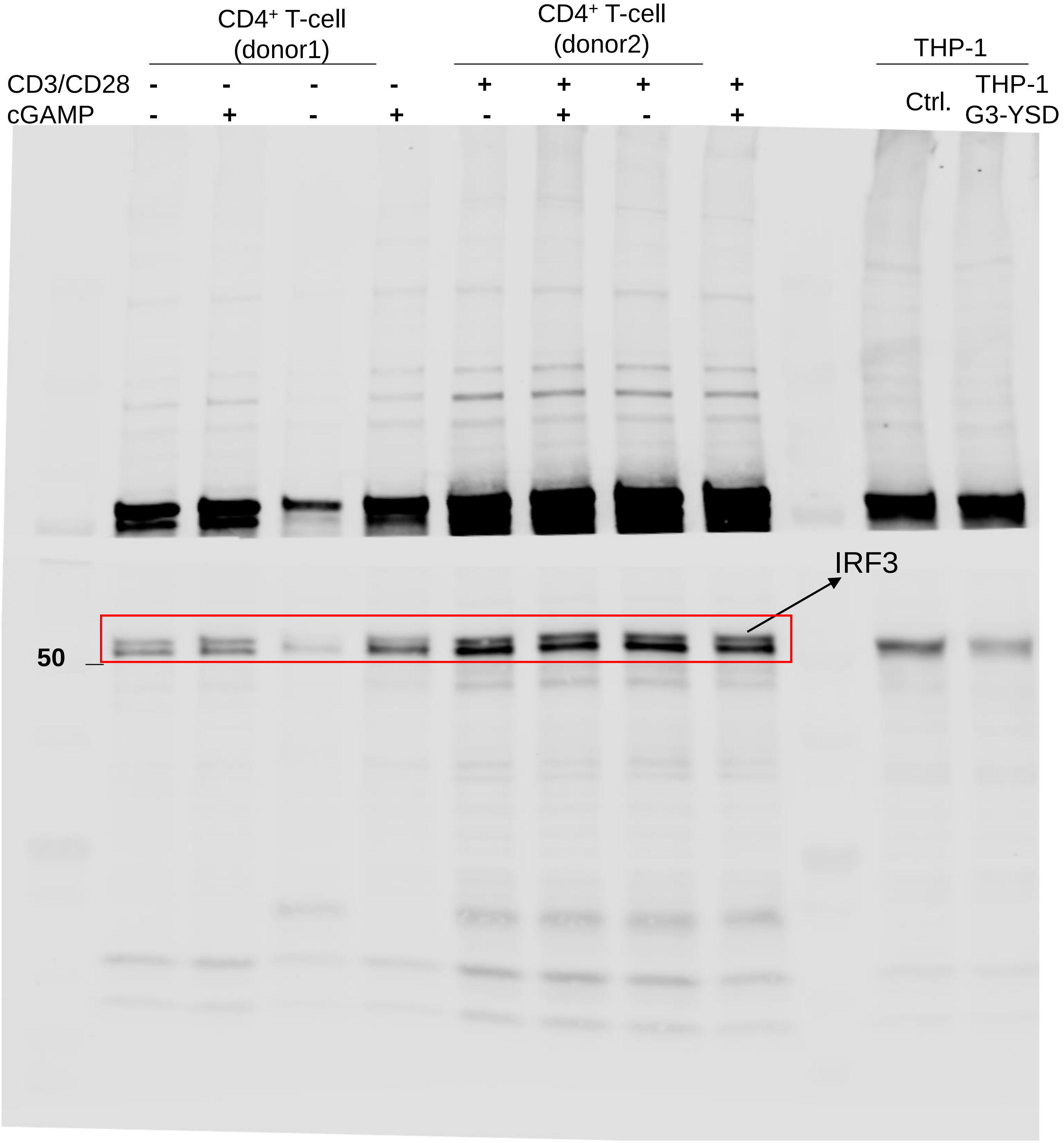

CD4+ T-cell (donor1)
CD4+ T-cell (donor2)
THP-1
CD3/CD28 - - - - + + + +
cGAMP - + - + - + - +
Ctrl.
THP-1 G3-YSD
IRF3
50

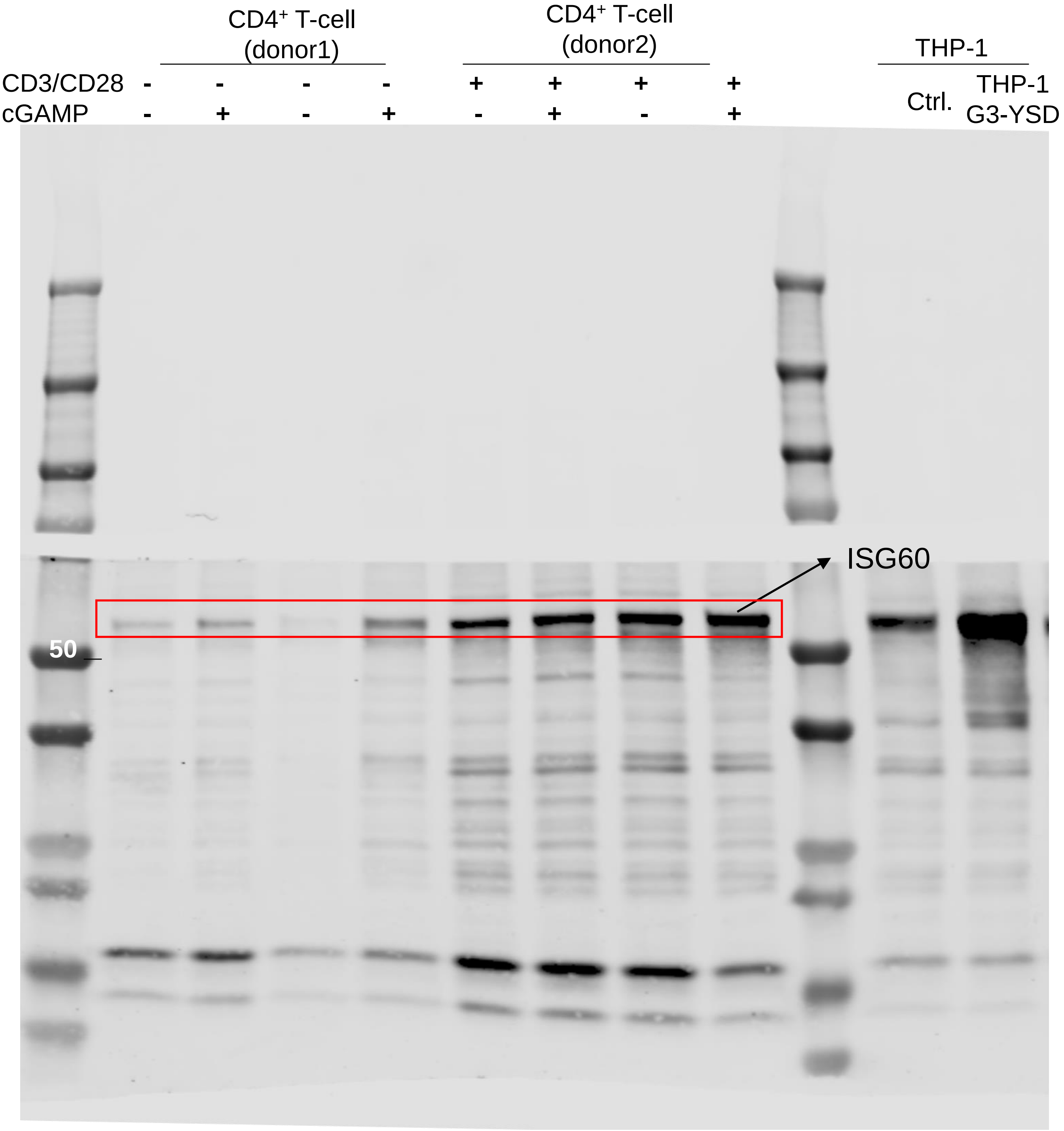

CD4+ T-cell (donor1)
CD4+ T-cell (donor2)
THP-1
CD3/CD28 - - - - + + + +
cGAMP - + - + - + - +
Ctrl.
THP-1 G3-YSD
ISG60
50

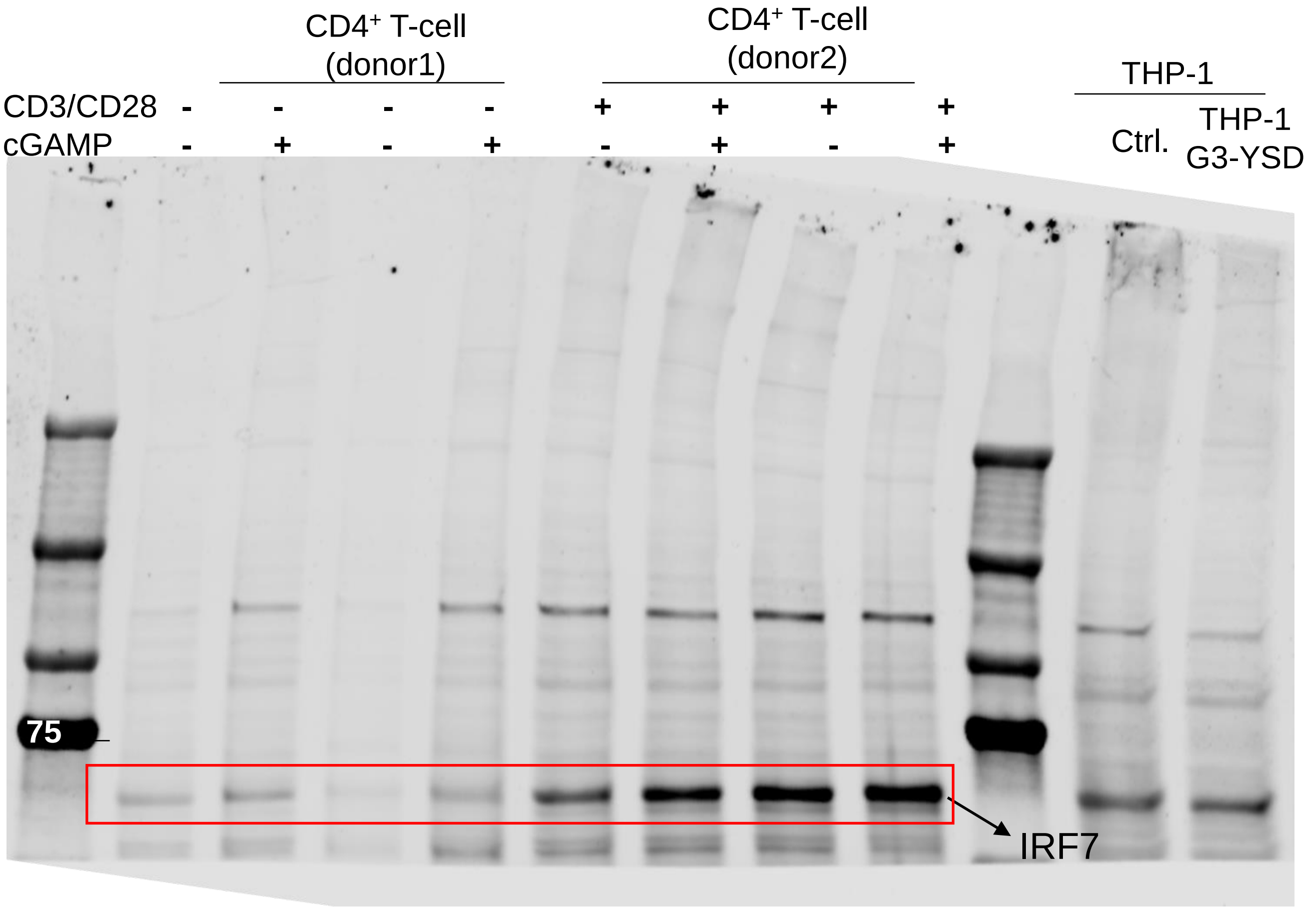
CD4+ T-cell (donor1)
CD4+ T-cell (donor2)
THP-1
CD3/CD28 - - - - + + + +
cGAMP - + - + - + - +
Ctrl.
THP-1 G3-YSD
75
IRF7

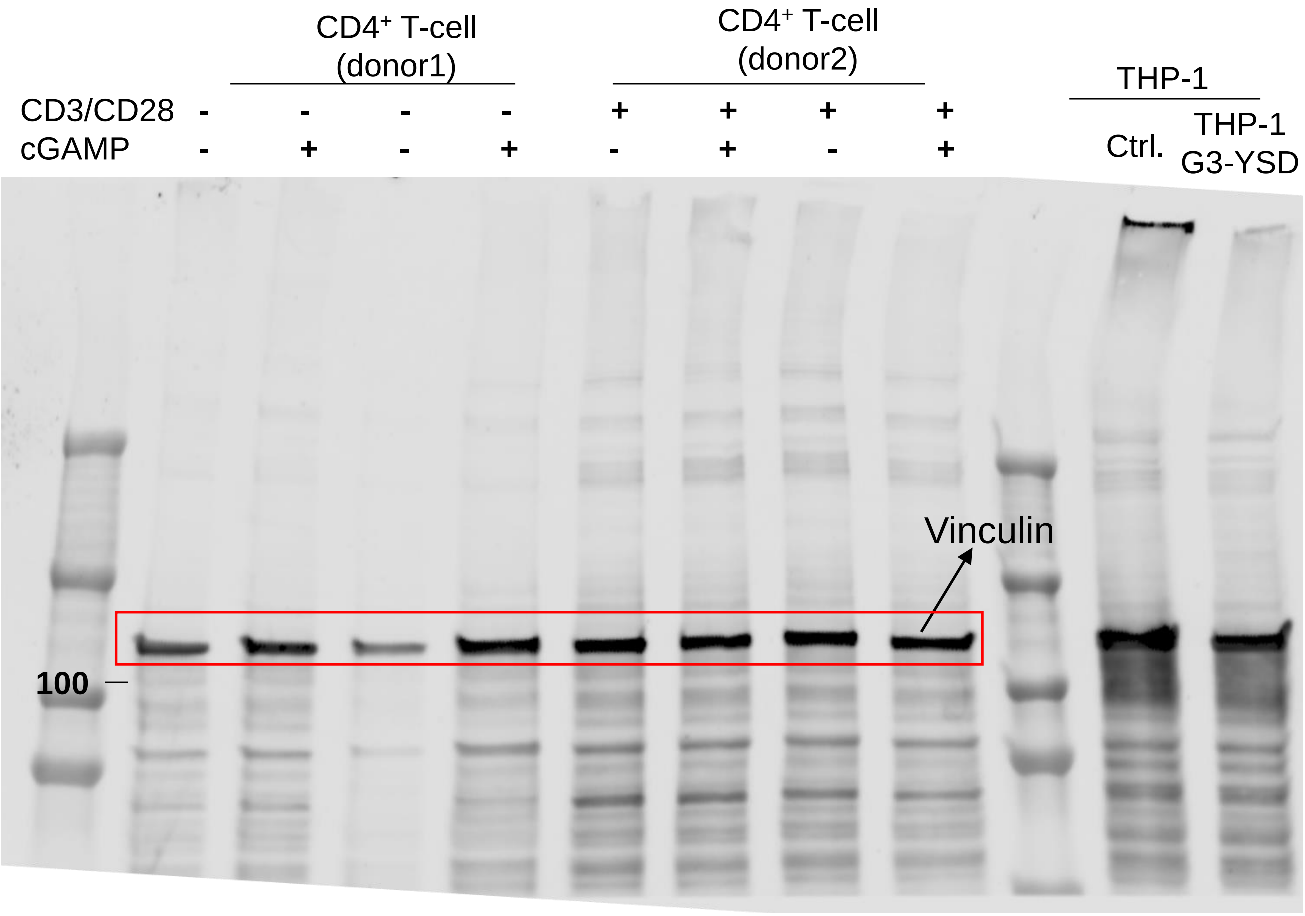
CD4+ T-cell (donor1)
CD4+ T-cell (donor2)
THP-1
CD3/CD28 - - - - + + + +
cGAMP - + - + - + - +
Ctrl.
THP-1 G3-YSD
Vinculin
100
